# Iridium-catalyzed asymmetric hydrogenation of racemic α-substituted lactones to chiral diols†
†Electronic supplementary information (ESI) available. See DOI: 10.1039/c6sc04609f



**DOI:** 10.1039/c6sc04609f

**Published:** 2016-11-15

**Authors:** Xiao-Hui Yang, Hai-Tao Yue, Na Yu, Yi-Pan Li, Jian-Hua Xie, Qi-Lin Zhou

**Affiliations:** a State Key Laboratory and Institute of Elemento-Organic Chemistry , Nankai University , Tianjin 300071 , China . Email: jhxie@nankai.edu.cn ; Email: qlzhou@nankai.edu.cn; b Collaborative Innovation Center of Chemical Science and Engineering (Tianjin) , Nankai University , Tianjin 300071 , China

## Abstract

We report a protocol for the highly efficient iridium-catalyzed asymmetric hydrogenation of racemic α-substituted lactones *via* dynamic kinetic resolution. Using Ir-SpiroPAP (*R*)-**1d** as a catalyst, a wide range of chiral diols were prepared in a high yield (80–95%) with a high enantioselectivity (up to 95% ee) under mild reaction conditions. This protocol was used for enantioselective syntheses of (–)-preclamol and a chiral 2,5-disubstituted tetrahydropyran.

## Abstract 2

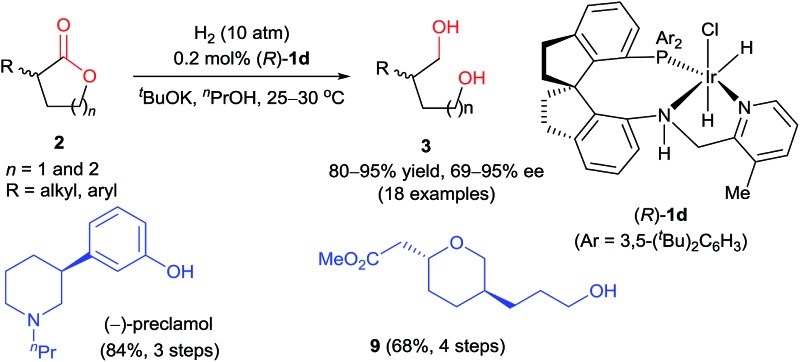
A protocol for the highly efficient iridium-catalyzed asymmetric hydrogenation of racemic α-substituted lactones *via* dynamic kinetic resolution is described.

## Introduction

**Scheme 1:**
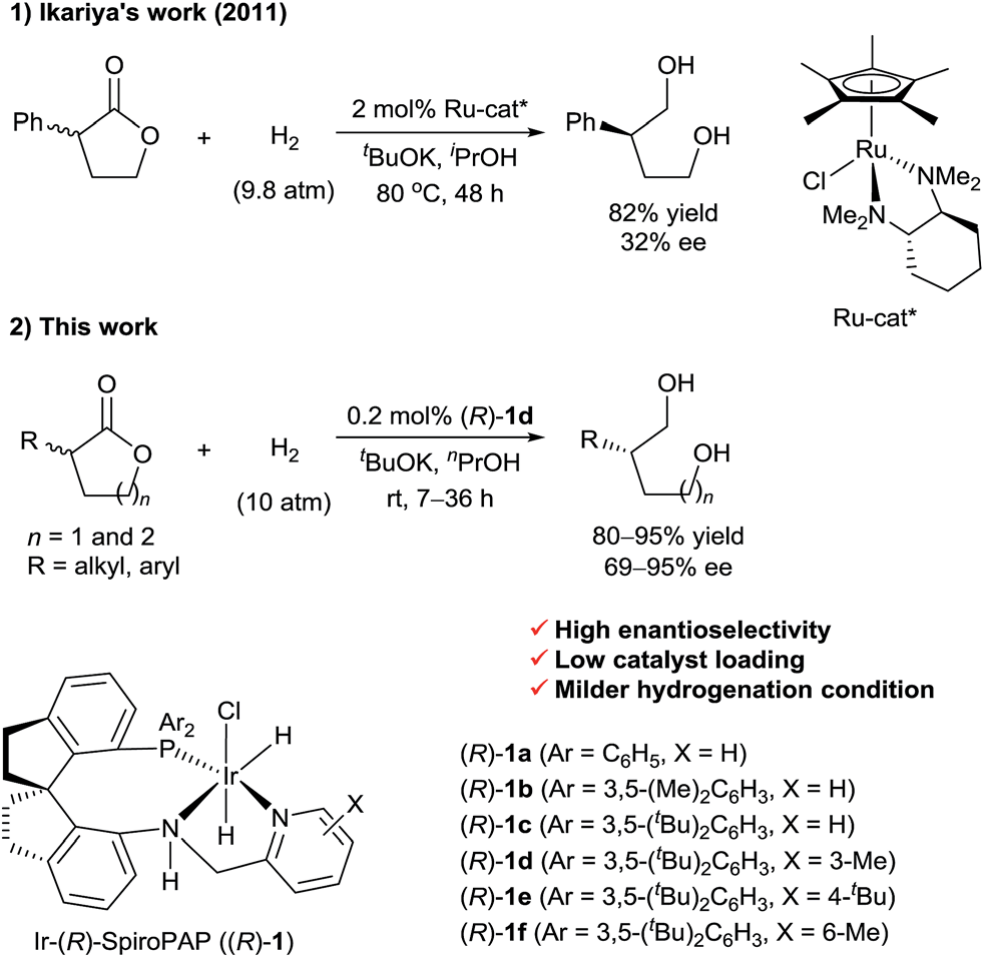
Asymmetric hydrogenation of racemic α-substituted lactones *via* DKR.

Transition-metal-catalyzed asymmetric hydrogenation of ketones is an efficient and reliable method for the synthesis of optically active chiral secondary alcohols.^1^ In contrast, the enantioselective synthesis of chiral primary alcohols by catalytic asymmetric hydrogenation of their corresponding aldehydes or esters is difficult, and work on the development of practical methods is underway. In 2007, we reported the first example of the catalytic asymmetric hydrogenation of racemic α-branched aldehydes, *via* dynamic kinetic resolution (DKR), for the synthesis of chiral primary alcohols.^2^ Subsequently, List^3^ and Lin^4^ *et al.* also reported the synthesis of chiral primary alcohols, by means of ruthenium-catalyzed asymmetric hydrogenation of racemic α-substituted aldehydes. Although a wide range of catalysts have been developed for the hydrogenation of esters,^5^ efficient chiral catalysts for the asymmetric hydrogenation of racemic α-substituted esters *via* DKR are rare. The biggest challenge for the direct asymmetric hydrogenation of racemic esters to form optically active primary alcohols is to find a catalyst that can discriminate between the enantiomers of chiral esters and then hydrogenate them to alcohols selectively. In 2011, Ikariya *et al.* ^6^ described the enantioselective hydrogenation of a racemic mixture of an α-substituted γ-lactone to a chiral 1,4-diol *via* DKR using chiral ruthenium catalysts bearing chiral 1,2-diamine ligands (Scheme 1). However, the enantioselectivity of the reaction was low (up to 32% ee). Recently, as part of our work on the asymmetric hydrogenation of ketones, we found that chiral Ru-SDPs/diamine catalysts and chiral Ir-SpiroPAP catalysts can also mediate the hydrogenation of ester groups.^7^

In this communication, we report a protocol for the Ir-SpiroPAP-catalyzed asymmetric hydrogenation of racemic α-substituted lactones to afford chiral diols in a high yield (80–95%) with a high enantioselectivity (up to 95% ee, Scheme 1).

## Results and discussion

We initially performed the hydrogenation of racemic α-phenyl δ-valerolactone (**2a**) to evaluate the activity and enantioselectivity of various catalysts (Table 1). Under the previously reported reaction conditions^7*b*^ (catalyst loading = 0.2 mol% (S/C = 500), [**2a**] = 0.25 M, [^*t*^BuOK] = 0.04 M, EtOH, 10 atm H_2_ and 25–30 °C), no reaction occurred in the presence of the catalyst (*R*)-**1d** (entry 1). However, when the concentration of ^*t*^BuOK was increased to 0.06 M, the hydrogenation reaction occurred and provided (*R*)-**3a** in 15% yield with 91% ee (entry 2); further increasing the concentration of ^*t*^BuOK increased the reaction rate and the yield of **3a** substantially. For example, when 0.25 M ^*t*^BuOK (**2a**/^*t*^BuOK/(*R*)-**1d** = 500 : 500 : 1) was used, the reaction was complete within 10 h, providing (*R*)-**3a** in 91% yield with 92% ee (entry 3). The absolute configuration of (*R*)-**3a** was determined by comparing the sign of its optical rotation with the literature data.^8^ Evaluation of various chiral Ir-SpiroPAP catalysts (*R*)-**1** revealed that the substituents on the pyridine and phenyl groups of the catalysts had little effect on the yield or enantioselectivity (entries 4–8), with (*R*)-**1d** giving the best results. Experiments with various solvents showed that ^*n*^PrOH was suitable (entries 9–11); the reaction was complete within 10 h, affording (*R*)-**3a** in 92% yield with 93% ee. In addition to ^*t*^BuOK, ^*t*^BuONa also gave a high yield with a high enantioselectivity, but the use of KOH, NaOH, or K_2_CO_3_ resulted in low yields (entries 12–15).

**Table 1:** Asymmetric hydrogenation of **2a**. Optimizing reaction conditions^*a*^

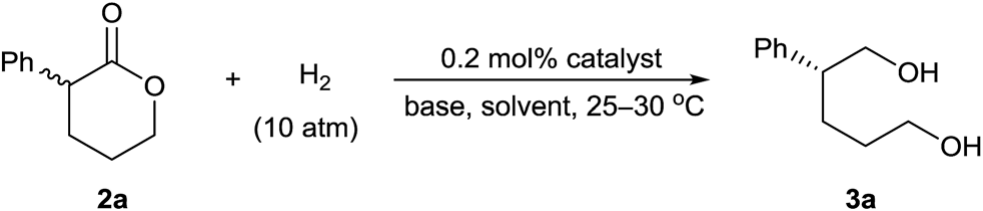							
Entry	Cat.	Base	[Base]	Solvent	Time (h)	Yield^*b*^ (%)	ee^*c*^ (%)
1	(*R*)-**1d**	^*t*^BuOK	0.04	EtOH	24	ND	ND
2	(*R*)-**1d**	^*t*^BuOK	0.06	EtOH	24	15	91
3	(*R*)-**1d**	^*t*^BuOK	0.25	EtOH	10	91	92
4	(*R*)-**1a**	^*t*^BuOK	0.25	EtOH	10	93	90
5	(*R*)-**1b**	^*t*^BuOK	0.25	EtOH	10	92	90
6	(*R*)-**1c**	^*t*^BuOK	0.25	EtOH	10	92	91
7	(*R*)-**1e**	^*t*^BuOK	0.25	EtOH	17	91	90
8	(*R*)-**1f**	^*t*^BuOK	0.25	EtOH	10	92	90
9	(*R*)-**1d**	^*t*^BuOK	0.25	MeOH	7	10	86
10	(*R*)-**1d**	^*t*^BuOK	0.25	^*n*^PrOH	10	92	93
11	(*R*)-**1d**	^*t*^BuOK	0.25	*^i^* PrOH	8	89	74
12	(*R*)-**1d**	^*t*^BuONa	0.25	^*n*^PrOH	10	91	93
13	(*R*)-**1d**	KOH	0.25	^*n*^PrOH	20	48	93
14	(*R*)-**1d**	NaOH	0.25	^*n*^PrOH	20	36	93
15	(*R*)-**1d**	K_2_CO_3_	0.25	^*n*^PrOH	20	21	92

^*a*^Reaction conditions: 1.0 mmol scale, [**2a**] = 0.25 M, 0.2 mol% of catalyst, solvent (4.0 mL) and room temperature (25–30 °C).

^*b*^Isolated yield.

^*c*^Determined by HPLC using a chiral column. The absolute configuration of **3a** is *R* determined by comparing its optical rotation with the literature data (see ESI).

To evaluate the substrate scope of the reaction, we investigated a wide range of racemic α-substituted δ-valerolactones under the established reaction conditions (Table 2). For racemic α-aryl-substituted δ-valerolactones **2a–i** (entries 1–9), neither electron-donating nor electron-withdrawing groups on the phenyl ring of the substrates had much effect on the enantioselectivity of the reaction, but substrates with an electron-withdrawing group (entries 2, 5, and 8) showed a higher reaction rate than those with an electron-donating group.

**Table 2:** Asymmetric hydrogenation of racemic α-substituted lactones to chiral diols with (*R*)-**1d** ^*a*^

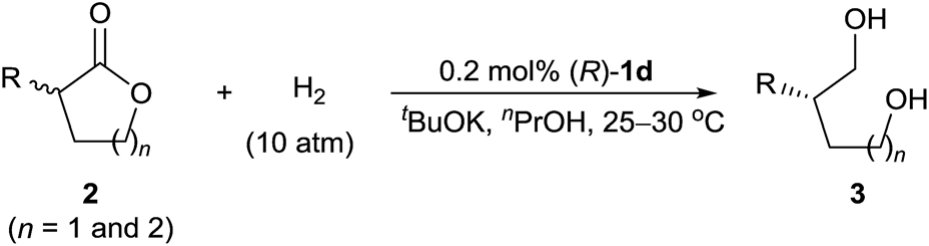						
Entry	R	*n*	**3**	Time (h)	Yield^*b*^ (%)	ee^*c*^ (%)
1^*d*^	C_6_H_5_	2	**3a**	10	92	93 (*R*)
2	4-ClC_6_H_4_	2	**3b**	7	93	93
3	4-MeC_6_H_4_	2	**3c**	9	92	93
4	4-MeOC_6_H_4_	2	**3d**	10	93	93
5	3-ClC_6_H_4_	2	**3e**	7	95	92
6	3-MeC_6_H_4_	2	**3f**	10	91	93
7	3-MeOC_6_H_4_	2	**3g**	10	93	92 (*R*)
8^*d*^	3,4-Cl_2_C_6_H_3_	2	**3h**	7	94	92
9	3,4-(MeO)_2_C_6_H_3_	2	**3i**	10	91	91
10	2-ClC_6_H_4_	2	**3j**	13	89	78
11	2-MeC_6_H_4_	2	**3k**	36	84	77
12	2-MeOC_6_H_4_	2	**3l**	20	88	86
13^*d*^	Me	2	**3m**	8	92	91 (*S*)
14	Et	2	**3n**	12	90	87
15^*d*^	*^i^* Pr	2	**3o**	12	90	95 (*R*)
16^*d*^	CH_2_ =CHCH_2_CH_2_	2	**3p**	12	91	88 (*S*)
17	C_6_H_5_	1	**3q**	10	80	80 (*R*)
18	Me	1	**3r**	10	82	69 (*S*)

^*a*^Reaction conditions: 1.0 mmol scale, [substrate] = 0.25 M, [^*t*^BuOK] = 0.25 M, 0.2 mol% of (*R*)-**1d**, ^*n*^PrOH (4.0 mL) and room temperature (25–30 °C).

^*b*^Isolated yield.

^*c*^Determined by HPLC using a chiral column.

^*d*^The absolute configuration of the product is determined by comparing its optical rotation with the literature data (see ESI).

In addition, owing to steric effects, substrates with an *ortho*-substituent on the phenyl ring gave lower reaction rates, yields, and enantioselectivities (entries 10–12). The hydrogenation of α-alkyl-substituted δ-valerolactones **2m–p** also worked well, affording the corresponding 1,5-diols **3m–p** in high yields and high enantioselectivities (entries 13–16). Catalyst (*R*)-**1d** also catalyzed the hydrogenation of racemic α-substituted γ-butyrolactones **2q** and **2r**, providing the 1,4-diols **3q** (80% ee) and **3r** (69% ee), respectively (entries 17 and 18).

**Fig. 1:**
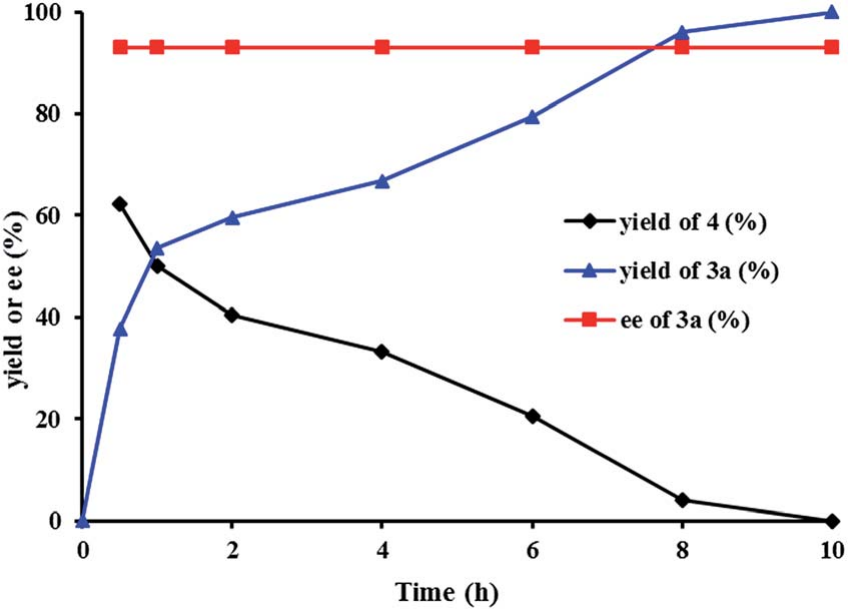
The plots of the hydrogenation of *rac*-**2a** with (*R*)-**1d**.

We investigated the pathway of the hydrogenation of racemic α-substituted lactones **2** by ^1^H NMR. As shown in Fig. 1, after reaction for 0.5 h under the optimal reaction conditions, *rac*-**2a** was converted to the hydroxyl ester **4**, propyl 5-hydroxy-2-phenylpentanoate, in 62% yield with no ee and the diol **3a** in 38% yield with 93% ee. Over the following 10 h, the amount of the hydroxyl ester **4** gradually decreased, and the amount of the diol **3a** increased. Only a trace amount of lactone **2a** was detected from 2 min after the reaction started.

**Scheme 2:**
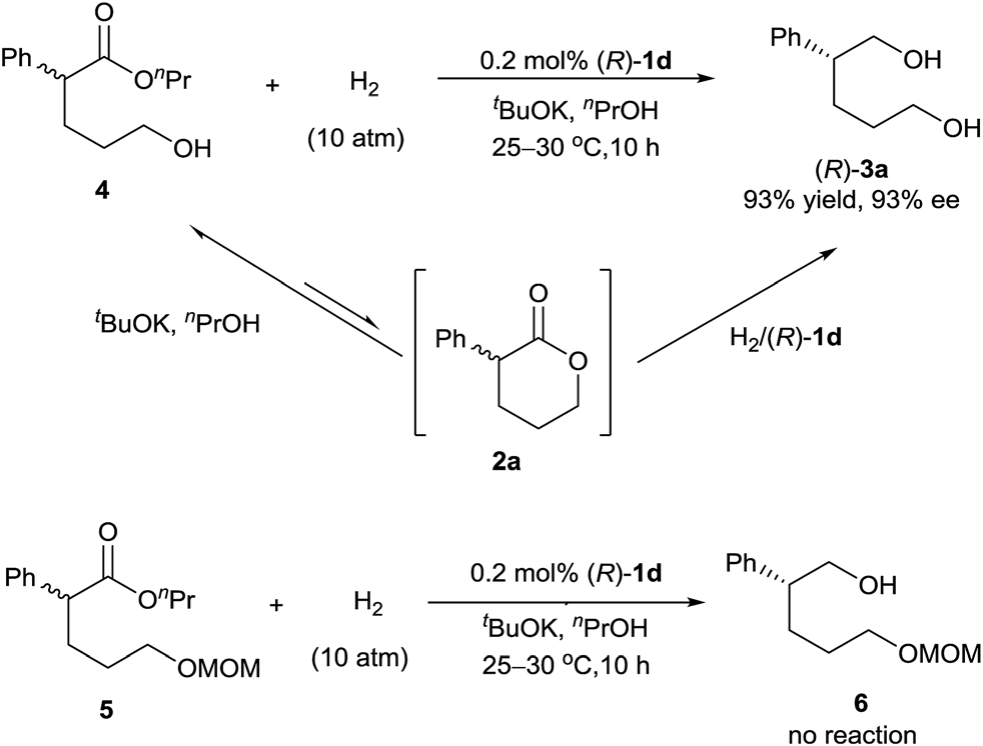
Asymmetric hydrogenation of esters **4** and **5** with (*R*)-**1d**.

Direct hydrogenation of the hydroxyl ester **4** with catalyst (*R*)-**1d** provided the diol **3a** in 93% yield with 93% ee, which is the same as the result obtained from the hydrogenation of lactone **2a** (Scheme 2). We also conducted the hydrogenation of the ester **5**, which has a δ-OCH_2_OMe group instead of a δ-OH group as in the hydroxyl ester **4**, and observed no reaction. These results indicated that the hydroxyl group of ester **4** was crucial for the hydrogenation. Thus, although the lactone **2a** was readily alcoholized to the hydroxyl ester **4** under the reaction conditions, the hydrogenation of **2a** occurred inevitably *via* its lactone form (Scheme 2).^7b,c^

**Scheme 3:**
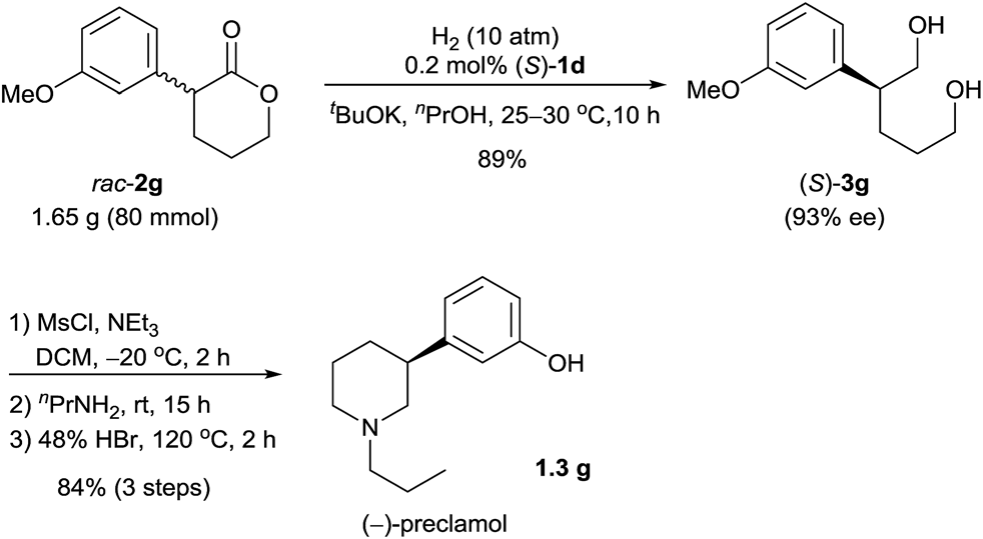
Enantioselective synthesis of (–)-preclamol.

Chiral 3-aryl/alkyl substituted piperidines are part of an important class of bioactive heterocyclic compounds,^9^ but they are difficult to synthesize in their optically active forms.^10^ By using the catalyst (*S*)-**1d**, we synthesized (–)-preclamol,^11^ which is a candidate drug for the treatment of neurological disorders such as Parkinson's disease.^12^ The hydrogenation of *rac*-**2g** (1.65 g) catalyzed by (*S*)-**1d** afforded the diol (*S*)-**3g** (89% yield and 93% ee), which was subsequently transformed to (–)-preclamol by activation with methanesulfonyl chloride, substitution/cyclization with *n*-propylamine, and demethylation with hydrobromic acid (84% yield over three steps, Scheme 3).

**Scheme 4:**
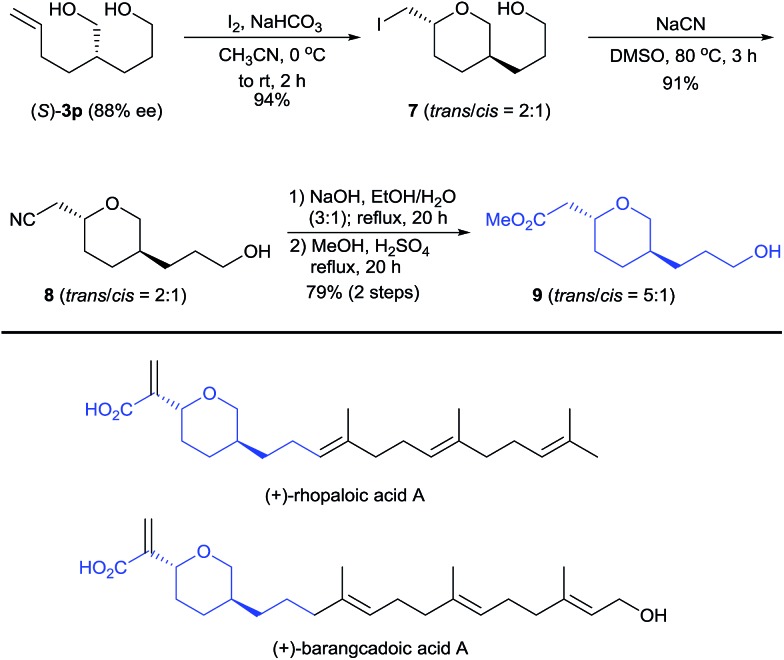
Enantioselective synthesis of a chiral 2,5-disubstituted tetrahydropyran.

Diol (*S*)-**3p** is a useful building block for the synthesis of chiral 2,5-disubstituted tetrahydropyrans, which occur in many biologically active natural products such as the terpenoids rhopaloic acid A^13^ and barangcadoic acid A^14^ (Scheme 4), isolated from marine sponges. Iodoetherification of (*S*)-**3p** with iodine^15^ produced the tetrahydropyran **7** in 94% yield as a 2 : 1 *trans*/*cis* mixture. Nucleophilic substitution of tetrahydropyran **7** with NaCN afforded the nitrile **8** (*trans*/*cis* = 2 : 1). Hydrolysis of the nitrile **8** and its subsequent esterification with MeOH afforded the tetrahydropyran **9** in a 72% yield with a higher *trans*/*cis* ratio (5 : 1). Thus, this protocol represents a potential method for the construction of the chiral core structures of rhopaloic acid A and barangcadoic acid A.^16^

## Conclusions

In conclusion, we have developed a protocol for the highly efficient iridium-catalyzed asymmetric hydrogenation of racemic α-substituted lactones *via* DKR. Using an Ir-SpiroPAP catalyst, a series of racemic α-substituted lactones were hydrogenated to chiral diols in high yield with high enantioselectivity under mild reaction conditions. The protocol was used for the enantioselective syntheses of (–)-preclamol and a chiral 2,5-disubstituted tetrahydropyran.

## Experimental

### General procedure for the asymmetric hydrogenation of **2**

Into a 20 mL hydrogenation vessel in an autoclave was added racemic α-substituted δ-valerolactone **2** (1.0 mmol), a solution of iridium catalyst (*R*)-**1d** in ^*n*^PrOH (dried with MS 4 Å for 12 h, 0.002 mmol mL^–1^, 1.0 mL, 0.002 mmol), a solution of ^*t*^BuOK in ^*n*^PrOH (0.5 mmol mL^–1^, 2.0 mL, 1.0 mmol) and ^*n*^PrOH (1.0 mL). The autoclave was purged with hydrogen by pressurizing to 5 atm and releasing the pressure. This procedure was repeated three times and then the autoclave was pressurized to 10 atm of H_2_. The reaction mixture was stirred at room temperature (25–30 °C) until no obvious hydrogen pressure drop was observed. The reaction mixture was then quenched with saturated NH_4_Cl (5 mL) and extracted with EtOAc (5 mL × 3). The combined extracts were washed with brine, dried over anhydrous MgSO_4_ and concentrated *in vacuo*. The residue was purified by flash column chromatography on a silica gel using petroleum ether/ethyl acetate as the eluent to afford the chiral diols **3**. The ee values of the chiral diols **3** were determined by HPLC using a chiral column.
