# Supplementary material for: Iridium-catalyzed asymmetric hydrogenation of racemic α-substituted lactones to chiral diols
Source: Chem Sci. 2016 Nov 15;8(3):1811–4. doi: 10.1039/c6sc04609f (PMC5396509; doi:10.1039/c6sc04609f)
Supplement: Supplementary file 1 [file SC-008-C6SC04609F-s001.pdf]

Electronic Supplementary Material (ESI) for Chemical Science.
This journal is © The Royal Society of Chemistry 2016

Supporting Information For

Iridium-Catalyzed Asymmetric Hydrogenation of Racemic α-Substituted Lactones to Chiral Diols

Xiao-Hui Yang,[a] Hai-Tao Yue,[a] Na Yu,[a] Yi-Pan Li,[a] Jian-Hua Xie,*[a] and Qi-Lin Zhou*[ab]

[a] *State Key Laboratory and Institute of Elemento-organic Chemistry, Nankai University, Tianjin 300071, China*

[b] *Collaborative Innovation Center of Chemical Science and Engineering (Tianjin), Nankai University, Tianjin 300071, China*

Contents

General..**S1**

(A) Preparation of racemic α-substituted lactones..**S2**

(B) Hydrogenation of racemic α-substituted lactones..**S5**

(C) Investigation the pathway of hydrogenation of racemic α-substituted lactones......................**S9**

(D) Asymmetric synthesis of (–)-preclamol and compound 8..**S11**

(E) NMR spectra of new compounds..**S14**

(F) HPLC charts for hydrogenation products..**S40**

General: All reactions and manipulations which are sensitive to moisture or air were performed under inert atmosphere of nitrogen. All chemicals were purchased from J & K, Acros and Aldrich, and were used as received. Hydrogen gas (99.999%) was purchased from Boc Gas Inc., Tianjin. Anhydrous THF was distilled from sodium benzophenone ketyl. Anhydrous CH_2Cl_2, ^{n}PrOH and Et_3N were freshly distilled from calcium hydride. Anhydrous EtOH was freshly distilled from magnesium. Melting points were measured on a RY-I apparatus and uncorrected. ^{1}H NMR spectra were recorded at 400 MHz on Bruker AV 400 spectrometer. ^{13}C NMR spectra were recorded at 100 MHz on Bruker AV 400 spectrometer. NMR spectra were recorded in deuterated chloroform ($CDCl_3$) as a solvent, with residual chloroform (δ 7.26 ppm. for ^{1}H NMR and δ 77.00 ppm. for ^{13}C NMR) or tetramethylsilane (TMS, δ 0.00 ppm. for ^{1}H NMR) taken as the inert standard, and were reported in ppm. Abbreviations for signal coupling are as follows: s, singlet; d, doublet; t, triplet; q, quartet; m, multiplet. Coupling constants were taken from the spectra directly and are uncorrected. Optical rotations were determined using a Perkin Elmer 341 polarimeter. HRMS were recorded on APEXII and ZAB-HS spectrometer. HPLC analyses were performed using Hewlett Packard Model HP1100 instruments with Chiralcel OJ-H, OD-H, AD-H, AS-H column.

(A) Preparation of racemic α-substituted δ-valerolactones

The racemic α-aryl substituted δ-valerolactones were prepared according to the literature method illustrated below.[1]

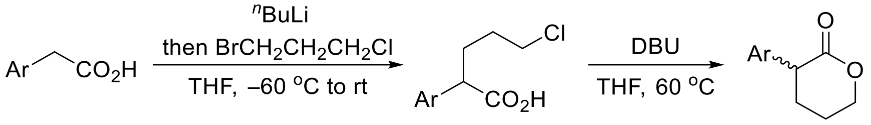

2a-l

To a solution of arylacetic acids (25.0 mmol) in dry THF (100 mL) was added *n*BuLi (20.8 mL, 50.0 mmol, 2.4 M in hexane) dropwise at –60 ℃ under N_2 atmosphere and the temperature was maintained below −40 ℃. After complete addition, the result mixture was slowly warmed to 0 ℃ and stirred at that temperature for 2 h. 1-Bromo-3-chloropropane (4.71 g, 30.0 mmol) was then added and the reaction allowed to warm to room temperature and stirred for 18 h. The reaction was quenched with 1N NaOH (50 mL) and transferred to a separatory funnel. The aqueous layer was collected and the organic extracted again with 1N NaOH (50 mL). The combined aqueous were re-acidified with 2N HCl (70 mL) and extracted with EtOAc (75 mL). The organic layer was washed with water (50 mL) and then concentrated in vacuo to yield an oil. The oil was redissolved in THF (50 mL), treated with DBU (3.74 mL, 25.0 mmol) and heated to 60 ℃ for 18 h. The resultant slurry was cooled to room temperature and filtered through a suction funnel. The solvent was concentrated in vacuo and the residue was purified by flash chromatography to offer the corresponding racemic α-aryl δ-valerolactones.

3-Phenyltetrahydro-2*H*-pyran-2-one (2a)[1]

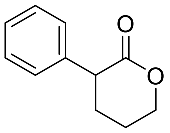

Colorless oil, 82% yield, ^{1}H NMR (400 MHz, $CDCl_3$) δ 7.38–7.32 (m, 2H), 7.30–7.21 (m, 3H), 4.52–4.38 (m, 2H), 3.78 (dd, *J* = 10.0, 7.2 Hz, 1H), 2.34–2.23 (m, 1H), 2.14–1.92 (m, 3H). ^{13}C NMR (100 MHz, $CDCl_3$) δ 172.5, 138.8, 128.6, 128.2, 127.3, 69.19, 47.0, 28.2, 21.9.

3-(4-Chlorophenyl)tetrahydro-2*H*-pyran-2-one (2b)

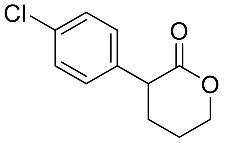

White solid, mp 50–52 °C, 78% yield. ^{1}H NMR (400 MHz, $CDCl_3$) δ 7.34–7.30 (m, 2H), 7.20–7.15 (m, 2H), 4.52–4.39 (m, 2H), 3.74 (dd, *J* = 10.4, 6.8 Hz, 1H), 2.34–2.21 (m, 1H), 2.11–1.95 (m, 3H). ^{13}C NMR (100 MHz, $CDCl_3$) δ 172.1, 137.2, 133.2, 129.6, 128.8, 69.2, 46.4, 28.1, 21.9. HRMS (ESI) Calcd for $C_{11}H_{11}ClO_2Na$ ($[M + Na]^+$): 233.0340, Found: 233.0339.

3-(4-Tolyl)tetrahydro-2*H*-pyran-2-one (2c)

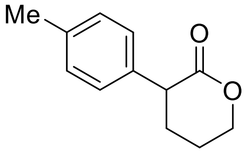

White solid, mp 42–44 °C, 77% yield. ^{1}H NMR (400 MHz, $CDCl_3$) δ 7.19–7.10 (m, 4H), 4.52–4.37 (m, 2H), 3.74 (dd, *J* = 10.0, 7.2 Hz, 1H), 2.34 (s, 3H), 2.31–2.22 (m, 1H), 2.13–1.94 (m, 3H). ^{13}C NMR (100 MHz, $CDCl_3$) δ 172.7, 136.80, 135.8, 129.3, 128.0, 69.1, 46.5, 28.0, 21.8, 20.9. IR (KBr): 2955, 1731, 1513, 1451, 1254, 1219, 1161, 1039, 817, 734 cm^{-1}. HRMS (ESI) Calcd for $C_{12}H_{14}O_2Na$ ($[M + Na]^+$): 213.0886, Found: 213.0892.

3-(4-Methoxyphenyl)tetrahydro-2*H*-pyran-2-one (2d)[2]

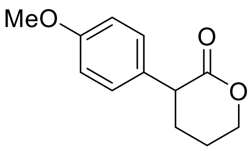

Colorless oil, 34% yield. ^{1}H NMR (400 MHz, $CDCl_3$) δ 7.19–7.13 (m, 2H), 6.92–6.85 (m, 2H), 4.50–4.38 (m, 2H), 3.80 (s, 3H), 3.73 (dd, *J* = 10.0, 6.8 Hz, 1H), 2.32–2.21 (m, 1H), 2.12–1.95 (m, 3H). ^{13}C NMR (100 MHz, $CDCl_3$) δ 172.9, 158.7, 130.8, 129.2, 114.1, 69.1, 55.2, 46.2, 28.1, 22.0.

3-(3-Chlorophenyl)tetrahydro-2*H*-pyran-2-one (2e)

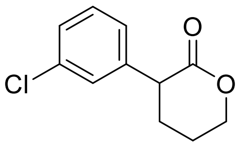

Colorless oil, 81% yield. ^{1}H NMR (400 MHz, $CDCl_3$) δ 7.32–7.22 (m, 3H), 7.13 (dt, *J* = 6.4, 2.0 Hz, 1H), 4.52–4.41 (m, 2H), 3.75 (dd, *J* = 10.4, 6.8 Hz, 1H), 2.34–2.23 (m, 1H), 2.11–1.97 (m, 3H). ^{13}C NMR (100 MHz, $CDCl_3$) δ 171. 8, 140.7, 134.4, 129.9, 128.5, 127.5, 126.5, 69.2, 46.7, 28.1, 21.9. HRMS (ESI) Calcd for $C_{11}H_{11}ClO_2Na$ ([M + Na]$^+$): 233.0340, Found: 233.0345.

3-(3-Tolyl)tetrahydro-2*H*-pyran-2-one (2f)[3]

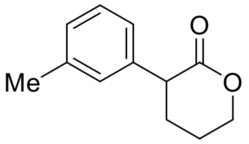

Colorless oil, 72% yield. ^{1}H NMR (400 MHz, $CDCl_3$) δ 7.22 (t, *J* = 7.2 Hz, 1H), 7.10–6.99 (m, 3H), 4.47–4.36 (m, 2H), 3.72 (dd, *J* = 9.6, 6.8 Hz, 1H), 2.33 (s, 3H), 2.29–2.20 (m, 1H), 2.11–1.91 (m, 3H). ^{13}C NMR (100 MHz, $CDCl_3$) δ 172.5, 138.8, 138.1, 128.8, 128.4, 127.9, 125.1, 69.1, 46.9, 28.1, 21.8, 21.3.

3-(3-Methoxyphenyl)tetrahydro-2*H*-pyran-2-one (2g)[4]

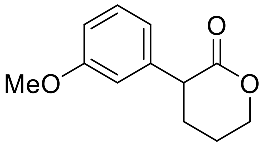

Colorless oil, 77% yield. ^{1}H NMR (400 MHz, $CDCl_3$) δ 7.26 (t, *J* = 8.0, 1H), 6.86–6.75 (m, 3H), 4.50–4.38 (m, 2H), 3.79 (s, 3H), 3.75 (dd, *J* = 10.0, 7.2 Hz, 1H), 2.34–2.22 (m, 1H), 2.14–1.91 (m, 3H). ^{13}C NMR (100 MHz, $CDCl_3$) δ 172.3, 159.7, 140.3, 129.6, 120.5, 114.2, 112.5, 69.2, 55.2, 47.1, 28.1, 21.8. HRMS (ESI) Calcd for $C_{12}H_{14}O_3Na$ ([M + Na]$^+$): 229.0836, Found: 229.0839.

3-(3,4-Dichlorophenyl)tetrahydro-2*H*-pyran-2-one (2h)

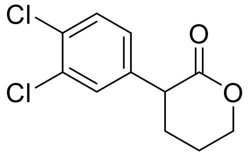

White solid, mp 96–98 °C, 43% yield. ^{1}H NMR (400 MHz, $CDCl_3$) δ 7.42 (d, *J* = 8.4 Hz, 1H), 7.34 (d, *J* = 2.0 Hz, 1H), 7.09 (dd, *J* = 8.4, 2.0 Hz, 1H), 4.52–4.40 (m, 2H), 3.72 (dd, *J* = 10.4, 7.2 Hz, 1H), 2.36–2.22 (m, 1H), 2.11–1.96 (m, 3H). ^{13}C NMR (100 MHz, $CDCl_3$) δ 171.5, 138.8, 132.6, 131.5, 130.6, 130.4, 127.8, 69.2, 46.2, 27.9, 22.0. IR (KBr): 2956, 1732, 1473, 1400, 1258, 1225, 1158, 1133, 1078, 1031, 961, 823 cm^{-1}. HRMS (ESI) Calcd for $C_{11}H_{10}ClO_2Na$ ([M + Na]$^+$): 266.9951, Found:266.9954.

3-(3,4-Dimethoxyphenyl)tetrahydro-2*H*-pyran-2-one (2i)

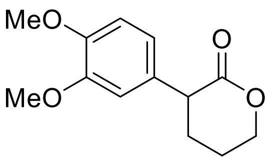

White solid, mp 98–100 °C, 47% yield. ^{1}H NMR (400 MHz, $CDCl_3$) δ 6.86–6.80 (m, 1H), 6.79–6.72 (m, 2H), 4.49–4.38 (m, 2H), 3.89–3.82 (m, 6H), 3.75–3.67 (m, 1H), 2.35–2.21 (m, 1H), 2.12–1.92 (m, 3H). ^{13}C NMR (100 MHz, $CDCl_3$) δ 172.7, 148.9, 148.2, 131.2, 120.2, 111.4, 111.2, 69.1, 55.8, 46.6, 28.0, 21.9. IR (KBr): 2940, 1731, 1592, 1518, 1464, 1420, 1259, 1143, 1078, 1026, 961, 810, 759 cm^{-1}. HRMS (ESI) Calcd for $C_{13}H_{16}O_4Na$ ([M + Na]$^+$): 259.0941, Found: 259.0945.

3-(2-Chlorophenyl)tetrahydro-2*H*-pyran-2-one (2j)

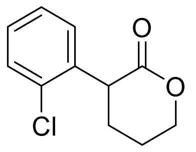

Colorless oil, 76% yield. ^{1}H NMR (400 MHz, $CDCl_3$) δ 7.56–7.34 (m, 1H), 7.27–7.18 (m, 3H), 4.56–4.43 (m, 2H), 4.07 (dd, J = 10.4, 7.2 Hz, 1H), 2.28–2.18 (m, 1H), 2.14–1.96 (m, 3H). ^{13}C NMR (100 MHz, $CDCl_3$) δ 171.2, 137.2, 133.2, 130.4, 129.94, 128.7, 127.2, 69.7, 45.6, 27.3, 22. 6. IR (KBr): 2957, 1732, 1475, 1443, 1215, 1168, 1036, 751 cm^{-1}. HRMS (ESI) Calcd for $C_{11}H_{11}ClO_2Na$ ([M + Na]$^+$): 233.0340, Found: 233.0344.

3-(2-Tolyl)tetrahydro-2*H*-pyran-2-one (2k)

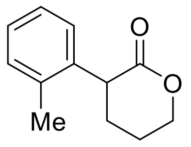

Colorless oil, 82% yield. ^{1}H NMR (400 MHz, $CDCl_3$) δ 7.22–7.17 (m, 3H), 7.17–7.13 (m, 1H), 4.57–4.44 (m, 2H), 3.94 (dd, J = 9.6, 7.2 Hz, 1H), 2.33 (s, 3H), 2.29–2.17 (m, 1H), 2.10–1.96 (m, 3H). ^{13}C NMR (100 MHz, $CDCl_3$) δ 172.2, 137.8, 135.6, 130.78, 128.1, 127.3, 126.3, 69.7, 44.5, 27.6, 22.3, 19.6. IR (KBr): 2957, 1731, 1491, 1462, 1208, 1160, 759, 733 cm^{-1}. HRMS (ESI) Calcd for $C_{12}H_{14}O_2Na$ ([M + Na]$^+$): 213.0886, Found: 213.0890.

3-(2-Methoxyphenyl)tetrahydro-2*H*-pyran-2-one (2l)

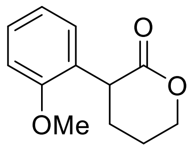

White solid, mp 86–88 °C, 55% yield. ^{1}H NMR (400 MHz, $CDCl_3$) δ 7.29–7.23 (m, 1H), 7.16 (dd, J = 7.2, 1.6 Hz, 1H), 6.96–6.88 (m, 2H), 4.54–4.40 (m, 2H), 3.83 (s, 3H), 3.77 (dd, J = 10.4, 7.6 Hz, 1H), 2.18–2.09 (m, 1H), 2.08–1.91 (m, 3H). ^{13}C NMR (100 MHz, $CDCl_3$) δ 172.2, 156.2, 129.9, 128.9, 128.6, 120.8, 111.2, 69.8, 55.4, 43.9, 27.8, 23.0. IR (KBr): 2945, 1729, 1495, 1463, 1246, 1157, 1082, 1025, 960, 755 cm^{-1}. HRMS (ESI) Calcd for $C_{12}H_{14}O_3Na$ ([M + Na]$^+$): 229.0836, Found: 229.0840.

The racemic α-alkyl substituted δ-valerolactones were prepared via α-alkylation of δ-valerolactones according to the literature method illustrated below.[5]

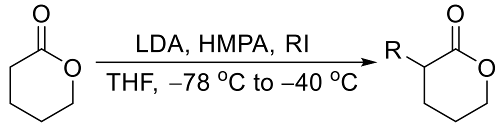

To a solution of diisopropylamine (5.05 g, 50 mmol) in dry THF (80 mL) was added *n*-BuLi (20 mL, 48 mmol, 2.4 M in hexane) dropwise at 0 ℃ under N_2 atmosphere and the result mixture was stirred at that temperature for 10 min. Then HMPA (8.96 g, 50 mmol) was added to the solution with stirring over a period of 10 min. The reaction mixture was cooled to −78 °C and δ-valerolactone (4.00 g, 40 mmol) in dry THF (160 mL) was added dropwise to the solution. After stirring 20 min, alkyl iodides (RI, 44 mmol) was added dropwise to the result solution and the result mixture was stirred at that temperature for 1 h. Then, the reaction solution was allowed to warm to −78 °C and stirred for further1 h. The reaction mixture was quenched with saturated NH_4Cl (50 mL). The residue was extracted with EtOAc (50 mL × 3). The combined organic layers were washed with brine, dried over anhydrous $MgSO_4$ and concentrated in vacuo. The residue was chromatographed on silica gel column with EtOAc/petroleum ether as an eluent to offer the corresponding racemic α-alkyl δ-valerolactones.

3-Methyltetrahydro-2*H*-pyran-2-one (2m)[4]

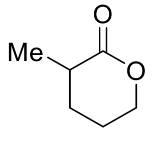

Colorless oil, 86% yield, ^{1}H NMR (400 MHz, $CDCl_3$) δ 4.28–4.16 (m, 2H), 2.55–2.42 (m, 1H), 2.07–1.95 (m, 1H), 1.87–1.76 (m, 2H), 1.51–1.35 (m, 1H), 1.14 (d, J = 6.8 Hz, 3H).

3-Ethyltetrahydro-2*H*-pyran-2-one (2n)[6]

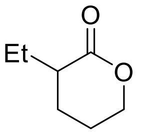

Colorless oil, 40% yield, ^{1}H NMR (400 MHz, $CDCl_3$) δ 4.35–4.17 (m, 2H), 2.43–2.28 (m, 1H), 2.12–1.99 (m, 1H), 1.94–1.77 (m, 3H), 1.58–1.44 (m, 2H), 0.94 (t, *J* = 7.6 Hz, 3H).

3-Isopropyltetrahydro-2*H*-pyran-2-one (2o)[7]

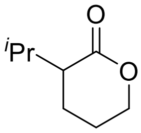

Colorless oil, 11% yield, ^{1}H NMR (400 MHz, $CDCl_3$) δ 4.40–4.17 (m, 2H), 2.49–2.32 (m, 2H), 2.01–1.81 (m, 3H), 1.71–1.52 (m, 1H), 0.99 (d, *J* = 5.6 Hz, 3H), 0.94 (d, *J* = 5.6 Hz, 3H).

3-(But-3-en-1-yl)tetrahydro-2H-pyran-2-one (2p)[8]

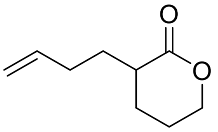

Colorless oil, 51% yield, ^{1}H NMR (400 MHz, $CDCl_3$) δ 5.81–5.68 (m, 1H), 5.04–4.90 (m, 2H), 4.31–4.19 (m, 2H), 2.49–2.37 (m, 1H), 2.19–1.93 (m, 4H), 1.92–1.79 (m, 2H), 1.57–1.43 (m, 2H).

(B) Asymmetric hydrogenation of racemic α-substituted lactones

General procedure: To a 20 mL hydrogenation vessel in an autoclave was added racemic α-substituted δ-valerolactone **2** (1.0 mmol), a solution of iridium catalyst (*R*)-**1d** in ^{n}PrOH (dried with MS 4Å for 12 h, 0.002 mmol/mL, 1.0 mL, 0.002 mmol), a solution of ^{t}BuOK in ^{n}PrOH (0.5 mmol/mL, 2.0 mL, 1.0 mmol) and ^{n}PrOH (1.0 mL). The autoclave was purged with hydrogen by pressurizing to 5 atm and releasing the pressure. This procedure was repeated three times and then pressurized to 10 atm of H_2. The reaction mixture was stirred at room temperature (25–30 °C) until no obvious hydrogen pressure drop was observed. The reaction mixture was then quenched with saturated NH_4Cl (5 mL) and extracted with EtOAc (5 mL × 3). The combined extracts were washed with brine, dried over anhydrous $MgSO_4$ and concentrated in vacuo. The residue was purified by flash column chromatography on silical gel with petroleum ether/ethyl acetate as an eluent to afford the chiral diols **3**.

The ee values of the chiral diols **3m, 3n, 3o, 3p** and **3r** were determined by transformation of them into the corresponding benzoyl esters. The general procedure was shown as follows: The chiral diols **3** (0.2 mmol) was reacted with benzoyl chloride (70 mg, 0.5 mmol) in the presence of pyridine (47 mg, 0.6 mmol) in DCM (5 mL) for 2 h. The reaction mixture was then quenched with saturated NH_4Cl (5 mL) and extracted with DCM (5 mL × 3). The combined extracts were washed with brine, dried over anhydrous $MgSO_4$ and concentrated in vacuo. After a flash chromatography on silica gel (petroleum ether/ethyl acetate = 10:1), the desired benzoyl esters were obtained in nearly quantitative yields.

(*R*)-2-Phenylpentane-1,5-diol (3a)[9]

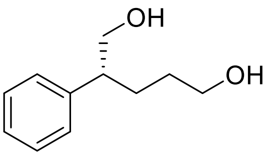

Colorless oil, 10 h, 92% yield, 93% ee, $[\alpha]_D^{20}$ −20.0 (*c* 1.0, EtOH) [lit.[9] $[\alpha]_D^{20}$ +55.0 (*c* 0.1, EtOH) for *S*-isomer]. ^{1}H NMR (400 MHz, $CDCl_3$) δ 7.36–7.29 (m, 2H), 7.27–7.17 (m, 3H), 3.73 (dd, *J* = 6.4, 2.4 Hz, 2H), 3.58 (t, *J* = 6.4 Hz, 2H), 2.84–2.72 (m, 1H), 1.89–1.73 (m, 3H), 1.69–1.56 (m, 1H), 1.54–

1.39 (m, 2H). ^{13}C NMR (100 MHz, $CDCl_3$) δ 142.1, 128.7, 128.0, 126. 8, 67.4, 62.6, 48.3, 30.3, 28.1. HPLC conditions: Chiralcel AS-H column (25 cm × 0.46 cm ID); *n*-hexane/2-propanol = 80:20; temp, rt; flow rate = 1.0 mL/min; 220 nm UV detector; t_R (*S*) = 5.63 min; t_R (*R*) = 6.03 min

(*R*)-2-(4-Chlorophenyl)pentane-1,5-diol (3b)

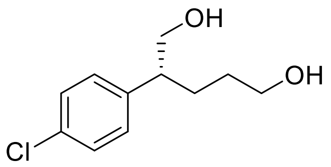

White solid, mp 66–68 ºC, 7 h, 93% yield, 93% ee, $[\alpha]_D^{20}$ −15.2 (*c* 1.0, EtOH). ^{1}H NMR (400 MHz, $CDCl_3$) δ 7.32–7.27 (m, 2H), 7.17–7.11 (m, 2H), 3.75–3.66 (m, 2H), 3.58 (t, *J* = 6.4 Hz, 2H), 2.83–2.69 (m, 1H), 1.88–1.66 (m, 3H), 1.64–1.52 (m, 1H), 1.50–1.37 (m, 2H). ^{13}C NMR (100 MHz, $CDCl_3$) δ 140.7, 132.4, 129.3, 128.8, 67.2, 62.6, 47.7, 30.2, 28.1. IR (KBr): 3284, 2929, 2879, 1491, 1091, 1055, 1040, 1013, 824 cm^{-1}. HPLC conditions: Chiralcel AS-H column (25 cm × 0.46 cm ID); *n*-hexane/2-propanol = 94:6; temp, rt; flow rate = 1.0 mL/min; 220 nm UV detector; t_R (*S*) = 21.16 min and t_R (*R*) = 22.82 min. HRMS (ESI) Calcd for $C_{11}H_{15}ClO_2Na$ ([M + Na]$^+$): 237.0653, Found: 237.0658.

(*R*)-2-(4-Tolyl)pentane-1,5-diol (3c)

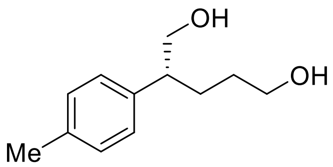

Colorless oil, 9 h, 92% yield, 93% ee, $[\alpha]_D^{20}$ −20.6 (*c* 1.0, EtOH). ^{1}H NMR (400 MHz, $CDCl_3$) δ 7.17–7.07 (m, 4H), 3.78–3.65 (m, 2H), 3.57 (t, *J* = 6.4 Hz, 2H), 2.81–2.69 (m, 1H), 2.32 (s, 3H), 1.87–1.76 (m, 3H), 1.65–1.54 (m, 1H), 1.52–1.39 (m, 2H). ^{13}C NMR (100 MHz, $CDCl_3$) δ 138.9, 136.3, 129.4, 127.9, 67.5, 62.7, 47.9, 30.4, 28.1, 21.0. IR (KBr): 3331, 2936, 2868, 1514, 1453, 1056, 1036, 815 cm^{-1}. HPLC conditions: Chiralcel OJ-H column (25 cm × 0.46 cm ID); *n*-hexane/2-propanol = 92:8; temp, rt; flow rate = 1.0 mL/min; 220 nm UV detector; t_R (*S*) = 13.81 min and t_R (*R*) = 15.47 min. HRMS (ESI) Calcd for $C_{12}H_{18}O_2Na$ ([M + Na]$^+$): 217.1199, Found: 217.1202.

(*R*)-2-(4-Methoxyphenyl)pentane-1,5-diol (3d)[2]

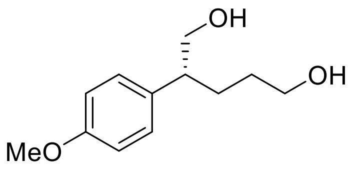

White solid, m.p. 48–50 ºC, 10 h, 93% yield, 93% ee, $[\alpha]_D^{20}$ −19.6 (*c* 1.0, EtOH). ^{1}H NMR (400 MHz, $CDCl_3$) δ 7.18–7.08 (m, 2H), 6.91–6.81 (m, 2H), 3.80 (s, 3H), 3.75–3.63 (m, 2H), 3.59 (t, *J* = 6.4 Hz, 2H), 2.81–2.67 (m, 1H), 1.88–1.71 (m, 3H), 1.69–1.55 (m, 1H), 1.53–1.41 (m, 2H). ^{13}C NMR (100 MHz, $CDCl_3$) δ 158.4, 143.4, 128.9, 114.1, 67.6, 62.8, 55.2, 47.5, 30.5, 28.2. HPLC conditions: Chiralcel AS-H column (25 cm ×0.46 cm ID); *n*-hexane/2-propanol = 80:20; temp, rt; flow rate = 1.0 mL/min; 220 nm UV detector; t_R (*S*) = 7.78 min; t_R (*R*) = 8.64 min.

(*R*)-2-(3-Chlorophenyl)pentane-1,5-diol (3e)

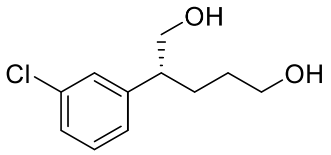

Colorless oil, 7 h, 95% yield, 92% ee, $[\alpha]_D^{20}$ −14.2 (*c* 1.0, EtOH). ^{1}H NMR (400 MHz, $CDCl_3$) δ 7.33–7.22 (m, 3H), 7.16–7.11 (m, 1H), 3.81–3.72 (m, 2H), 3.63 (t, *J* = 6.4 Hz, 2H), 2.86–2.75 (m, 1H), 2,00–1.82 (m, 3H), 1.69–1.57 (m, 1H), 1.56–1.42 (m, 2H). ^{13}C NMR (100 MHz, $CDCl_3$) δ 144.5, 134.4, 129.9, 128.1, 126.9, 126.3, 67.1, 62.5, 48.1, 30.21, 28.0. IR (KBr): 3319, 2839, 2871, 1596, 1571, 1474, 1430, 1055, 1037, 785, 746, 698 cm^{-1}. HPLC conditions: Chiralcel OJ-H column (25 cm × 0.46 cm ID); *n*-hexane/2-propanol = 92:8; temp, rt; flow rate = 1.0 mL/min; 220 nm

UV detector; t_R (*S*) = 11.84 min and t_R (*R*) = 12.42 min. HRMS (ESI) Calcd for $C_{11}H_{15}ClO_2Na$ ([M + Na]$^+$): 237.0653, Found: 237.0658.

(*R*)-2-(3-Tolyl)pentane-1,5-diol (3f)

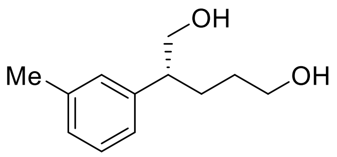

Colorless oil, 10 h, 91% yield, 93% ee, $[\alpha]_D^{20}$ –21.0 (*c* 1.0, EtOH). ^1H NMR (400 MHz, $CDCl_3$) δ 7.25–7.18 (m, 1H), 7.08–6.97 (m, 3H), 3.77–3.68 (m, 2H), 3.59 (t, *J* = 6.4 Hz, 2H), 2.80–2.70 (m, 1H), 2.34 (s, 3H), 1.87–1.76 (m, 1H), 1.71 (s, 2H), 1.67–1.57 (m, 1H), 1.53–1.41 (m, 2H). ^{13}C NMR (100 MHz, $CDCl_3$) δ 142.0, 138.3, 128.8, 128.6, 127.6, 125.0, 67.5, 62.7, 48.3, 30.4, 28.1, 21.5. IR (KBr): 3331, 2937, 2868, 1607, 1454, 1056, 1038, 785, 704 cm^{-1}. HPLC conditions: Chiralcel AS-H column (25 cm × 0.46 cm ID); *n*-hexane/2-propanol = 90:10; temp, rt; flow rate = 1.0 mL/min; 220 nm UV detector; t_R (*S*) = 10.03 min; t_R (*R*) = 11.44 min. HRMS (ESI) Calcd for $C_{12}H_{18}O_2Na$ ([M + Na]$^+$): 217.1199, Found: 217.1201.

(*R*)-2-(3-Methoxyphenyl)pentane-1,5-diol (3g)[10]

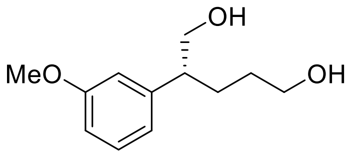

Colorless oil, 10 h, 93% yield, 92% ee, $[\alpha]_D^{20}$ –10.2 (*c* 1.0, $CHCl_3$). [lit.[10] $[\alpha]_D^{20}$ +10.7 (*c* 1.0, $CHCl_3$) for *S*-isomer]. ^1H NMR (400 MHz, $CDCl_3$) δ 7.27–7.22 (m, 1H), 6.83–6.74 (m, 3H), 3.80 (s, 3H), 3.73 (dd, *J* = 6.8, 2.7 Hz, 2H), 3.59 (t, *J* = 6.4 Hz, 2H), 2.83–2.71 (m, 1H), 1.88–1.72 (m, 3H), 1.68–1.56 (m, 1H), 1.53–1.41 (m, 2H). ^{13}C NMR (100 MHz, $CDCl_3$) δ 159.8, 143.8, 129. 7, 120.3, 114.0, 111.7, 67.4, 62.7, 55.1, 48.4, 30.4, 28.1. HPLC conditions: Chiralcel OJ-H column (25 cm × 0.46 cm ID); *n*-hexane/2-propanol = 90:10; temp, rt; flow rate = 1.0 mL/min; 220 nm UV detector; t_R (*S*) = 17.07 min and t_R (*R*) = 19.76 min.

(*R*)-2-(3,4-Dichlorophenyl)pentane-1,5-diol (3h)

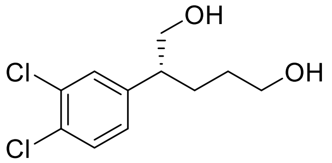

Colorless oil, 7 h, 94% yield, 92% ee, $[\alpha]_D^{20}$ –8.8 (*c* 1.0, EtOH). ^1H NMR (400 MHz, $CDCl_3$) δ 7.40 (d, *J* = 8.0 Hz, 1H), 7.32 (d, *J* = 2.0 Hz, 1H), 7.07 (dd, *J* = 8.0, 2.0 Hz, 1H), 3.81–3.68 (m, 2H), 3.62 (t, *J* = 6.4 Hz, 2H), 2.82–2.71 (m, 1H), 1.89–1.78 (m, 1H), 1.68–1.55 (m, 1H), 1.53–1.38 (m, 3H), 1.32 (s, 1H). ^{13}C NMR (100 MHz, $CDCl_3$) δ 142.9, 132.5, 130.5, 130. 5, 129. 9, 127.4, 66.7, 62.3, 47.4, 30.0, 27.9. IR (KBr): 3319, 2937, 2872, 1470, 1403, 1132, 1057, 1029, 821, 618 cm^{-1}. HPLC conditions: Chiralcel OD-H column (25 cm × 0.46 cm ID); *n*-hexane/2-propanol = 97:3; temp, rt; flow rate = 1.0 mL/min; 220 nm UV detector; t_R (*S*) = 54.51 min and t_R (*R*) = 57.04 min. HRMS (ESI) Calcd for $C_{11}H_{14}Cl_2O_2Na$ ([M + Na]$^+$): 271.0264, Found: 271.0265.

(*R*)-2-(3,4-Dimethoxyphenyl)pentane-1,5-diol (3i)

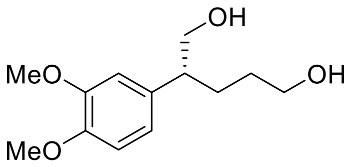

Colorless oil, 10 h, 91% yield, 91% ee, $[\alpha]_D^{20}$ –17.6 (*c* 1.0, EtOH). ^1H NMR (400 MHz, $CDCl_3$) δ 6.82 (d, *J* = 8.4 Hz, 1H), 6.78–6.70 (m, 2H), 3.87 (s, 3H), 3.85 (s, 3H), 3.76–3.65 (m, 2H), 3.59 (t, *J* = 6.4 Hz, 2H), 2.79–2.66 (m, 1H), 1.85–1.75 (m, 1H), 1.72–1.54 (m, 3H), 1.52–1.42 (m, 2H). ^{13}C NMR (100 MHz, $CDCl_3$) δ 149.1, 147.8, 134.5, 119.9, 111.4, 111.0, 67.5, 62.7, 55.9, 48.0, 30.4, 28.2. IR (KBr): 3347, 2936, 2871, 1517, 1461, 1261, 1234, 1141, 1027, 810, 763 cm^{-1}. HPLC conditions: Chiralcel OJ-H column (25 cm × 0.46 cm ID);

n-hexane/2-propanol = 90:10; temp, rt; flow rate = 1.0 mL/min; 220 nm UV detector; t_R (*R*) = 45.61 min and t_R (*S*) = 52.24 min. HRMS (ESI) Calcd for $C_{13}H_{20}O_4Na$ ($[M + Na]^+$): 263.1254, Found: 263.1258.

(*R*)-2-(2-Chlorophenyl)pentane-1,5-diol (3j)

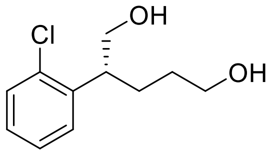

Colorless oil, 13 h, 89% yield, 78% ee, $[\alpha]^{20}_{D}$ −20.0 (*c* 1.0, EtOH). ^1H NMR (400 MHz, $CDCl_3$) δ 7.41–7.36 (m, 1H), 7.30–7.23 (m, 2H), 7.21–7.13 (m, 1H), 3.83–3.73 (m, 2H), 3.62 (t, *J* = 6.4, 2H), 3.53–3.43 (m, 1H), 1.98–1.87 (m, 1H), 1.84–1.61 (m, 3H), 1.60–1.41 (m, 2H). ^{13}C NMR (100 MHz, $CDCl_3$) δ 139.6, 134.9, 129.8, 128.0, 127.7, 127.1, 66.2, 62.7, 43.2, 30.1, 27.5. IR (KBr): 3318, 2940, 2869, 1475, 1439, 1043, 754, 687 cm^{-1}. HPLC conditions: Chiralcel OJ-H column (25 cm × 0.46 cm ID); *n*-hexane/2-propanol = 90:10; temp, rt; flow rate = 1.0 mL/min; 220 nm UV detector; t_R (*S*) = 10.00 min and t_R (*R*) = 13.11 min. HRMS (ESI) Calcd for $C_{11}H_{15}ClO_2Na$ ($[M + Na]^+$): 237.0653, Found: 237.0660.

(*R*)-2-(2-Tolyl)pentane-1,5-diol (3k)

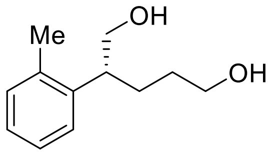

Colorless oil, 36 h, 84% yield, 77% ee, $[\alpha]^{20}_{D}$ −20.9 (*c* 1.0, EtOH). ^1H NMR (400 MHz, $CDCl_3$) δ 7.23–7.08 (m, 4H), 3.69 (d, *J* = 6.8 Hz, 2H), 3.55 (td, *J* = 6.4, 1.6 Hz, 2H), 3.19–3.09 (m, 1H), 2.34 (s, 3H), 2.24 (s, 2H), 1.94–1.81 (m, 1H), 1.69–1.56 (m, 1H), 1.52–1.35 (m, 2H). ^{13}C NMR (100 MHz, $CDCl_3$) δ 140.3, 137.0, 130.5, 126.3, 126.2, 125.8, 67.0, 62.6, 42.5, 30.2, 28.1, 19.9. IR (KBr): 3315, 2941, 2868, 1490, 1461, 1265, 1061, 1035, 756, 733 cm^{-1}. HPLC conditions: Chiralcel AD-H column (25 cm × 0.46 cm ID); *n*-hexane/2-propanol = 90:10; temp, rt; flow rate = 1.0 mL/min; 220 nm UV detector; t_R (*S*) = 15.89 min and t_R (*R*) = 16.77 min. HRMS (ESI) Calcd for $C_{12}H_{18}O_2Na$ ($[M + Na]^+$): 217.1199, Found: 217.1203.

(*R*)-2-(2-Methoxyphenyl)pentane-1,5-diol (3l)

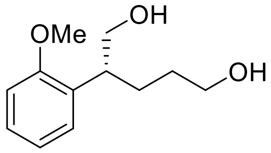

White solid, m.p. 88–90°C, 20 h, 88% yield, 86% ee, $[\alpha]^{20}_{D}$ −21.8 (*c* 1.0, EtOH). ^1H NMR (400 MHz, $CDCl_3$) δ 7.23–7.14 (m, 2H), 6.93 (td, *J* = 7.6, 0.8 Hz, 1H), 6.87 (dd, *J* = 8.4, 0.8 Hz, 1H), 3.80 (s, 3H), 3.73 (dd, *J* = 6.4, 3.2 Hz, 2H), 3.58 (td, *J* = 6.4, 1.6 Hz, 2H), 3.56–3.27 (m, 1H), 2.04 (s, 2H), 1.90–1.79 (m, 1H), 1.71–1.60 (m, 1H), 1.53–1.43 (m, 2H). ^{13}C NMR (100 MHz, $CDCl_3$) δ 157.6, 130.2, 127.9, 127.4, 120.7, 110.7, 66.4, 62.7, 55.4, 40.5, 30.3, 27.1. IR (KBr): 3325, 2938, 2868, 1492, 1462, 1240, 751 cm^{-1}. HPLC conditions: Chiralcel OJ-H column (25 cm × 0.46 cm ID); *n*-hexane/2-propanol = 90:10; temp, rt; flow rate = 1.0 mL/min; 220 nm UV detector; t_R (*S*) = 10.20 min and t_R (*R*) = 12.40 min. HRMS (ESI) Calcd for $C_{12}H_{18}O_3Na$ ($[M + Na]^+$): 233.1149, Found: 233.1152.

(*S*)-2-Methylpentane-1,5-diol (3m)[11]

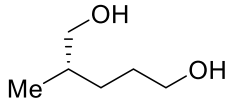

Colorless oil, 8 h, 92% yield, 91% ee, $[\alpha]^{20}_{D}$ −16.7 (*c* 1.0, $CHCl_3$). [lit.[11] $[\alpha]^{29}_{D}$ −18 (*c* 0.8, $CHCl_3$)]. ^1H NMR (400 MHz, $CDCl_3$) δ 3.58 (t, *J* = 6.0 Hz, 2H), 3.46–3.36 (m, 4H), 1.66–1.55 (m, 2H), 1.54–1.44 (m, 2H), 1.18–1.06 (m, 1H), 0.87 (d, *J* = 6.8 Hz, 3H). HPLC conditions: Chiralcel OD-H column (25 cm × 0.46 cm ID); *n*-hexane/2-propanol = 90:10; temp, rt; flow rate = 1.0 mL/min; 220 nm UV detector; t_R (*R*) = 11.54 min; t_R (*S*) = 12.66 min

(*S*)-2-Ethylpentane-1,5-diol (3n)

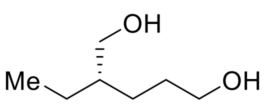

Colorless oil, 12 h, 90% yield, 87% ee, $[\alpha]_D^{20}$ −3.0 (c 1.0, $CHCl_3$). ^1H NMR (400 MHz, $CDCl_3$) δ 3.62–3.53 (m, 3H), 3.49–3.39 (m, 3H), 1.58–1.49 (m, 2H), 1.44–1.35 (m, 2H), 1.35–1.25 (m, 3H), 0.86 (t, J = 7.2 Hz, 3H). ^{13}C NMR (100 MHz, $CDCl_3$) δ 64.5, 62.7, 41.5, 29.3, 26.4, 23.4, 11.2. IR (KBr): 3305, 2930, 2873, 1456, 1379, 1052 cm^{-1}. HPLC conditions: Chiralcel OD-H column (25 cm × 0.46 cm ID); n-hexane/2-propanol = 99:1; temp, rt; flow rate = 1.0 mL/min; 220 nm UV detector; t_R (R) = 18.58 min; t_R (S) = 19.85 min

(*R*)-2-Isopropylpentane-1,5-diol (3o)[11]

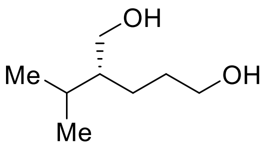

Colorless oil, 12 h, 90% yield, 95% ee, $[\alpha]_D^{20}$ −7.3 (c 1.0, $CHCl_3$). [lit.[11] $[\alpha]_D^{23}$ −7.11 (c 0.6, $CHCl_3$)]. ^1H NMR (400 MHz, $CDCl_3$) δ 3.72–3.60 (m, 3H), 3.58–3.51 (m, 1H), 2.07 (s, 2H), 1.83–1.68 (m, 1H), 1.66–1.54 (m, 2H), 1.50–1.29 (m, 3H), 0.91 (s, 3H), 0.90 (s, 3H). HPLC conditions: Chiralcel OD-H column (25 cm × 0.46 cm ID); n-hexane/2-propanol = 98:2; temp, rt; flow rate = 1.0 mL/min; 220 nm UV detector; t_R (S) = 11.24 min; t_R (R) = 12.02 min.

(*S*)-2-(But-3-en-1-yl)pentane-1,5-diol (3p)

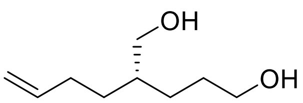

Colorless oil, 12 h, 91% yield, 88% ee, $[\alpha]_D^{20}$ −4.3 (c 1.0, C HCl3). ^1H NMR (400 MHz, $CDCl_3$) δ 5.87–5.68 (m, 1H), 4.96 (dd, J = 29.6, 16.8 Hz, 2H), 3.65–3.44 (m, 6H), 2.10–2.00 (m, 2H), 1.59–1.29 (m, 7H). ^{13}C NMR (100 MHz, $CDCl_3$) δ 138.8, 114.4, 64.7, 62.7, 39.4, 31.1, 30.1, 29.3, 26.7. HPLC conditions: Chiralcel OD-H column (25 cm × 0.46 cm ID); n-hexane/2-propanol = 99:1; temp, rt; flow rate = 1.0 mL/min; 220 nm UV detector; t_R (R) = 23.17 min; t_R (S) = 24.95 min. HRMS (ESI) Calcd for $C_{13}H_{20}O_4Na$ ($[M + Na]^+$): 181.1199, Found: 181.1195.

(*R*)-2-Phenylbutane-1,4-diol (3q)[12]

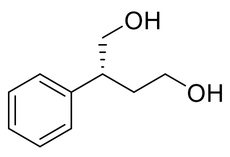

Colorless oil, 10 h, 80% yield, 80% ee, $[\alpha]_D^{20}$ −19.0 (c 1.0, $CHCl_3$). [lit.[12] $[\alpha]_D^{20}$ −39 (c 0.3, $CHCl_3$)]. ^1H NMR (400 MHz, $CDCl_3$) δ 7.34–7.28 (m, 2H), 7.25–7.19 (m, 3H), 3.73 (dd, J = 6.8, 2.8 Hz, 2H), 3.69–3.61 (m, 1H), 3.57–3.49 (m, 1H), 3.27 (brs, 2H), 2.99–2.87 (m, 1H), 2.09–1.95 (m, 1H), 1.93–1.79 (m, 1H). HPLC conditions: Chiralcel AD-3 column (25 cm × 0.46 cm ID); n-hexane/2-propanol = 97:3; temp, rt; flow rate = 1.0 mL/min; 220 nm UV detector; t_R (S) = 50.70 min; t_R (R) = 50.78 min.

(*S*)-2-Methylbutane-1,4-diol (3r)[13]

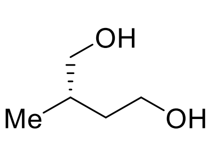

Colorless oil, 10 h, 82% yield, 69.1% ee, $[\alpha]_D^{20}$ −9.4 (c 1.0, $CHCl_3$). [lit.[13] $[\alpha]_D^{24}$ +22.6 (c 0.6, $CHCl_3$) for R-isomer]. ^1H NMR (400 MHz, $CDCl_3$) δ 3.81–3.70 (m, 1H), 3.67–3.58 (m, 2H), 3.56–3.50 (m, 2H), 3.39 (dd, J = 10.8, 7.6 Hz, 1H), 1.85–1.74 (m, 1H), 1.66–1.45 (m, 2H), 0.91 (d, J = 6.8 Hz, 3H). HPLC conditions: Chiralcel OD-H column (25 cm × 0.46 cm ID); n-hexane/2-propanol = 95:5; temp, rt; flow rate = 1.0 mL/min; 220 nm UV detector; t_R (R) = 7.89 min; t_R (S) = 8.65 min.

(C) Investigation the pathway of hydrogenation of racemic α-substituted lactones

Monitoring of the hydrogenation of *rac*-2a by NMR

According to the general procedure for the asymmetric hydrogenation of *rac*-**2**, the hydrogenations

of *rac*-**2a** (1.0 mmol-scale) were performed in parallel with (*R*)-**1d** as a catalyst and stopped after reaction for 0.5, 1, 2, 4, 6, 8, 10 h, respectively. The conversions or yields and ee values were determined by ^{1}H NMR and HPLC. The data and the plot are outlined in Table S1. For the reason of the lactone *rac*-**2a** was readily alcoholized with alcohol under the reaction conditions, we observed the formation of propyl 5-hydroxy-2-phenylpentanoate (**4**) during the hydrogenations.

Propyl 5-hydroxy-2-phenylpentanoate (4):

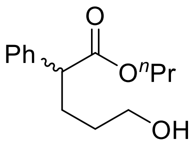

Colorless oil. ^{1}H NMR (400 MHz, $CDCl_3$) δ 7.38–7.20 (m, 5H), 4.20–3.87 (m, 2H), 3.66 (t, 2H), 3.59 (t, *J* = 7.7 Hz, 1H), 2.29–2.07 (m, 1H), 2.00–1.79 (m, 1H), 1.67–1.49 (m, 4H), 0.88 (t, *J* = 7.4 Hz, 3H). ^{13}C NMR (400 MHz, $CDCl_3$) δ 174.2, 139.1, 128.6, 127.9, 127.29, 66.4, 62.4, 51.5, 30.7, 29.7, 22.0, 10.3. IR (KBr): 3310, 2919, 1731, 1455, 1261, 1160, 750 cm^{-1}. HRMS (ESI) Calcd for $C_{14}H_{20}O_3H$ ([M + H]$^+$): 237.1485, Found: 237.1483.

Table S1 The data of asymmetric hydrogenation of *rac*-**2a**.

Entry	Time (h)	**3a**		**4**	
		Yield (%)	ee (%)	Yield (%)	ee (%)
1	0	0	–	–	–
2	0.5	37.7	93	62.3	*rac*
3	1	53.5	93	46.5	*rac*
4	2	59.5	93	40.5	*rac*
5	4	66.7	93	33.3	*rac*
6	6	79.4	93	20.6	*rac*
7	8	96.0	93	4.0	*rac*
8	10	100	93	0	–

Asymmetric hydrogenation of hydroxyl ester *rac*-4 and MOM-protected hydroxyl ester *rac*-5

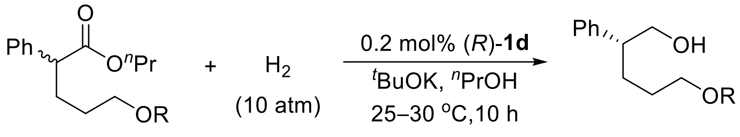

4 (R = H)
5 (R = CH_2OMe)

(*R*)-**3a** (R = H), 93% yield, 93% ee
6 (R = CH_2OMe), no reaction

The hydrogenations of hydroxyl ester *rac*-**4** and MOM-protected hydroxyl ester *rac*-**5** were performed according to the general procedure for the asymmetric hydrogenation of *rac*-**2** (1.0 mmol-scale) with (*R*)-**1d** as a catalyst in ^{n}PrOH.

Hydroxyl ester *rac*-4: 10 h, 93% yield, 93% ee.

MOM-protected hydroxyl ester *rac*-5: 10 h, no reaction.

The MOM-protected hydroxyl ester *rac*-**5** was synthesized as below according to literature method.[14]

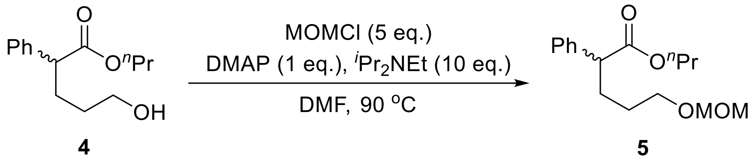

To a stirred solution of DMAP (61 mg, 0.5 mmol) and compound 4 (118 mg, 0.5 mol) in DMF (2 mL) was added $^{i}Pr_2NEt$ (1.1 mL) and MOMCl (190 μL, 2.5 mmol) at room temperature under N_2 atmosphere. The resulting mixture was allowed to warm up to 90 °C. After the reaction mixture stirred for 3 h. DMF was removed in vacuo and the residue was diluted with EtOAc (10 mL) and washed with saturated $NaHCO_3$, brine and dried over $MgSO_4$ and concentrated in vacuo to afford a crude product, which was purified by flash column chromatography on silical gel (petroleum ether/ethyl acetate = 8:1) to afford the compound **5** (123 mg, 88% yield)**.** ^1H NMR (400 MHz, $CDCl_3$) δ 7.36–7.15 (m, 5H), 4.59 (s, 2H), 4.13–3.86 (m, 2H), 3.54 (dt, J = 12.8, 7.1 Hz, 3H), 3.34 (s, 3H), 2.27–2.00 (m, 1H), 1.98–1.75 (m, 1H), 1.64–1.38 (m, 4H), 0.85 (t, J = 7.4 Hz, 3H).^{13}C NMR (101 MHz, $CDCl_3$) δ 173.0, 138.1, 127.6, 126.9, 126.2, 95.3, 66.2, 65.3, 54.1, 50.5, 29.1, 26.7, 20.9. IR (KBr): 2931, 2879, 1731, 1454, 1147, 1110, 1039, 917, 698 cm^{-1}. HRMS (ESI) Calcd for $C_{16}H_{24}O_4H$ ($[M + H]^+$): 281.1747, Found: 281.1743.

(D) Asymmetric synthesis of (–)-preclamol and compound 8

Asymmetric synthesis of (–)-preclamol

The asymmetric synthesis of (–)-preclamol was outlined below.

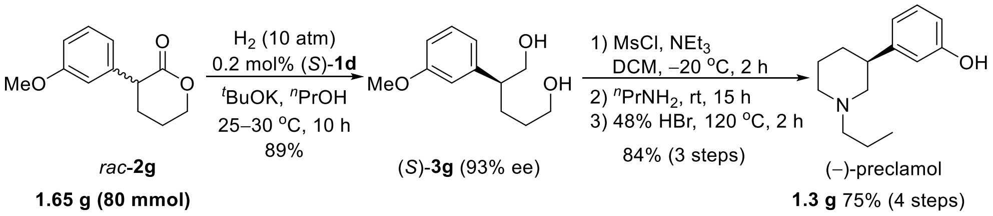

The asymmetric hydrogenation of *rac*-**2g** at 1.65 g (80 mmol) scale was according to the general procedure for the asymmetric hydrogenation of racemic α-substituted δ-valerolactones and under the same reaction conditions (10 atm, 10 h), and yielded (*S*)-**3g** in 89% yield (1.50 g) with 93% ee.

To a solution of (*S*)-**3g** (1.49 g, 7.1 mmol) in DCM (40 mL) was added NEt_3 (3.0 mL, 21.3 mmol) and MsCl (1.4 mL, 17.8 mmol) at –20 °C under N_2 atmosphere. After the reaction mixture was stirred for 2 h, saturated $NaHCO_3$ (20 mL) was added. The layers were separated and the aqueous layer was extracted with DCM (20 mL × 3). The combined organic layers were washed with brine, dried over $MgSO_4$ and concentrated in vacuo to afford a crude residue. Then, $^{n}PrNH_2$ (20 mL) was added to the residue at room temperature and the reaction mixture was stirred for overnight. $^{n}PrNH_2$ was removed in vacuo and the residue was diluted with EtOAc (10 mL) and washed with saturated $NaHCO_3$, brine and dried over $MgSO_4$, concentrated in vacuo afford a crude product. To the crude product was added 48% HBr (20 mL) and the mixture was stirred at 120 °C for 2 h. After cooling to room temperature, saturated $NaHCO_3$ was added to the reaction mixture to pH 7 at 0 °C. The solution was extracted with DCM (20 mL × 3), and the combined extractes were washed with brine, dried over anhydrous $MgSO_4$ and concentrated in vacuo to afford a crude product, which was purified by flash column chromatography on silical gel (petroleum ether/ethyl acetate = 1:1) to afford the (–)-preclamol (1.31 g, 84% yield over three

steps) as a yellow oil.[4] $[\alpha]^{20}_{D}$ −19.1 (*c* 2.0, $CHCl_3$) [lit.[4] $[\alpha]^{20}_{D}$ −20.9 (*c* 1.8, $CHCl_3$)]. ^{1}H NMR (400 MHz, $CDCl_3$) δ 9.35 (s, 1H), 7.18 (t, *J* = 8.0 Hz, 1H), 6.79 (s, 1H), 6.76–6.68 (m, 2H), 3.24 (d, *J* = 11.2 Hz, 1H), 3.09 (d, *J* = 10.8 Hz, 1H), 2.97–2.90 (m, 1H), 2.47–2.27 (m, 2H), 2.07–1.91 (m, 3H), 1.89–1.70 (m, 2H), 1.61–1.48 (m, 3H), 0.87 (t, *J* = 7.2 Hz, 3H). ^{13}C NMR (100 MHz, $CDCl_3$) δ 157.0, 145.3, 129.8, 117.2, 114.7, 114.3, 61.4, 61.2, 54.0, 41. 8, 29.8, 25.2, 19.2, 12.0.

Asymmetric synthesis of compound 9

The asymmetric synthesis of compound **9** was outlined below.

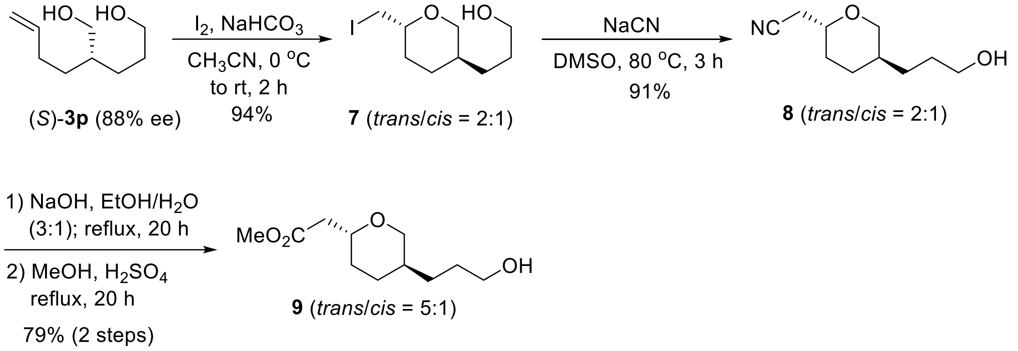

To a solution of (*S*)-**3p** (790 mg, 5.0 mmol) in CH_3CN (60 mL) was added $NaHCO_3$ (1.26 g, 15 mmol) at room temperature under N_2 atmosphere. The reaction mixture was vigorously stirred for 15 min and cooling to 0 °C followed by addition of I_2. After at room temperature stirring for 2 h, saturated Na_2SO_3 was added to the solution. The layers were separated and the aqueous layer was extracted with DCM (30 mL × 3). The combined organic layers were washed with brine, dried over $MgSO_4$ and concentrated in vacuo and the residue was purified by flash chromatography (petroleum ether/ethyl acetate = 5:1) to offer the corresponding compound **7** (1.33 g, 94% yield) as a mixture of *trans* and *cis*-isomer in a ratio of 2/1. Colorless oil. $[\alpha]^{20}_{D}$ +5.4 (*c* 1.0, $CHCl_3$). ^{1}H NMR (400 MHz, $CDCl_3$) δ 4.07–3.99 (m, 0.7H), 3.87–3.80 (m, 0.3H), 3.73–3.60 (m, 2.3H), 3.43–3.33 (m, 0.3H), 3.31–3.18 (m, 2.7H), 3.12 (t, *J* = 11.2 Hz, 0.7H), 2.00–1.91 (m, 0.7H), 1.90–1.83 (m, 0.7H), 1.79–1.72 (m, 0.6H), 1.68–1.47 (m, 5H), 1.43–1.12 (m, 3H). ^{13}C NMR (100 MHz, $CDCl_3$) δ 76.8, 76.7, 73.6, 70.8, 62.7, 62. 7, 35.1, 33.0, 31.45, 30.6, 29.8, 29.6, 28.1, 26.8, 26.8, 26.1, 9.73, 9.67. HRMS (ESI) Calcd for $C_9H_{18}IO_2$ ([M + H]$^+$): 285.0346, Found: 285.0346.

To the solution of **7** (1.33 g, 4.7 mmol) in DMSO (50 mL) was added NaCN (490 mg, 10 mmol). The reaction mixture was heated at 80 °C for 3 h and then cooled to room temperature. The solvent was removed in vacuo and the result was added H_2O (20 mL). The solution was extracted with EtOAc (20 mL × 3) and the combined extractes were washed with brine, dried over anhydrous $MgSO_4$ and concentrated in vacuo to afford the desired compound **8** as a colorless oil (0.78 g, 91% yield). $[\alpha]^{20}_{D}$ −1.0 (*c* 1.0, $CHCl_3$). ^{1}H NMR (400 MHz, $CDCl_3$) δ 4.03–3.95 (m, 0.65 H), 3.80 (d, *J* = 11.2 Hz, 0.35H), 3.71–3.57 (m, 2.70H), 3.53–3.48 (m, 0.65H), 3.08 (t, *J* = 11.2 Hz, 0.65H), 2.52 (m, 2H), 2.02–1.92 (m, 0.65H), 1.83–1.71 (m, 1.35H), 1.67–1.52 (m, 4H), 1.51–1.40 (m, 1H), 1.28–1.11 (m, 2H). ^{13}C NMR (101 MHz, $CDCl_3$) δ 117.4, 73.6, 72.8, 71.0, 62. 8, 62.7, 35.0, 32.8, 30.9, 30.7, 29. 7, 29.6, 28.3, 26.7, 26.3, 26.0, 24.7, 24.5. HRMS (ESI) Calcd for $C_{10}H_{21}N_2O_2$ ([M + NH_4]$^+$): 201.1603, Found: 201.1598.

The compound **8** (0.78 g, 4.3 mmol) was then added to a solution of NaOH (2.0 g, 50.0 mmol) in 50 mL of a 3:1 mixture of EtOH/H_2O. The reaction mixture was heated to reflux for 20 h. After cooling to 0 °C, the reaction mixture was acidated with 2 N HCl to pH > 1. The solution was concentrated in vacuo again and the remaining solution was extracted with EtOAc (20 mL × 3). The combined extractes

were washed with brine, dried over anhydrous $MgSO_4$ and concentrated in vacuo to afford the desired carboxylic acid as a crude product. The crude carboxylic acid was then redissolved in MeOH (20 mL) and several drops of *conc*. H_2SO_4 (0.5–1.0 mL) was added. The reaction mixture was refluxed for 20 h and concentrated in vacuo to yield a residue. The residue was diluted with EtOAc (20 mL), and neutralized with 1 N NaOH solution to pH 7 at 0 °C. The organic solution was separated and the aqueous solution was extracted with EtOAc (20 mL × 3). The combined organic solution was then dried over anhydrous $MgSO_4$, concentrated in vacuo and the residue was purified by flash chromatography (petroleum ether/ethyl acetate = 10:1) to offer the corresponding compound **9** (0.73 g, 79% yield) as a colorless oil (*trans*/*cis* = 5:1). $[\alpha]_D^{20}$ −10.9 (*c* 1.0, $CHCl_3$). ^1H NMR (400 MHz, $CDCl_3$) δ 3.91–3.83 (m, 1H), 3.71–3.59 (m, 4H), 3.54 (t, *J* = 8.4 Hz, 2H), 3.01 (t, *J* = 11.2 Hz, 1H), 2.47 (dd, *J* = 15.2, 8.0 Hz, 1H), 2.35 (dd, *J* = 15.2, 4.8 Hz, 1H), 2.14 (s, 1H), 1.90–1.80 (m, 1H), 1.70–1.58 (m, 1H), 1.57–1.38 (m, 3H), 1.35–1.22 (m, 1H), 1.21–1.01 (m, 3H). ^{13}C NMR (100 MHz, $CDCl_3$) δ 171.8, 74.19, 73.4, 62.6, 51. 6, 41.1, 35.2, 31.3, 30.0, 29.6, 28.3. HRMS (ESI) Calcd for $C_{11}H_{20}O_4Na$ ($[M + Na]^+$): 239.1254, Found: 239.1252.

References:

1. J. D. Rosen, T. D. Nelson, M. A. Huffman and J. M. McNamara, *Tetrahedron Lett.* 2003, **44**, 365.
2. M. Ito, T. Ootsuka, R. Watari, A. Shiibashi, A. Himizu and T. Ikariya, *J. Am. Chem. Soc.* 2011, **133**, 4240.
3. J. S. Harvey, S. P. Simonovich, C. R. Jamison and D. W. C. MacMillan, *J. Am. Chem. Soc.* 2011, **133**, 13782.
4. Z. Huang, Z. Chen, L. H. Lim, G. C. P. Quang, H. Hirao and J. Zhou, *Angew. Chem., Int. Ed.* 2013, **52**, 5807.
5. B. W. Boal, A. W. Schammel and N. K. Garg, *Org. Lett.* 2009, **11**, 3458.
6. M. P. Edwards, S. V. Ley, G. Simon, S. G. Lister, B. D. Palmer and D. J. Williams, *J. Org. Chem.* 1984, **49**, 3503.
7. L. Ayala, C. G. Lucero, J. A. C. Romero, S. A. Tabacco and K. A. Woerpel, *J. Am. Chem. Soc.* 2003, **125**, 15521.
8. A. J. Fry, R. D. Little and J. Leonetti, *J. Org. Chem.* 1994, **59**, 5017
9. S. S. M. A. Hakim and T. Sugimura, *Org. Lett.* 2010, **12**, 3626.
10. J, Y. Hamilton, D. Sarlah and E. M.d Carreira, *Angew. Chem. Int. Ed.* 2015, **54**, 7644.
11. T. Kano, F. Shirozu, M. Akakura and K. Maruoka, *J. Am. Chem. Soc*. 2012, **134**, 16068.
12. H. Krause and C. Sailer, *J. Organomet. Chem.* 1992, **423**, 271.
13. D. R. Williams, L. A. Robinson, C. R. Nevill and J. P. Reddy, *Angew. Chem. Int. Ed.* 2007, **46**, 915.
14. M. Keita, M. Vandamme and J.-F. Paquin, *Synthesis*, 2015, **47**, 3758.

(E) NMR spectra of new compounds

3-(4-Chlorophenyl)tetrahydro-2*H*-pyran-2-one (2b)

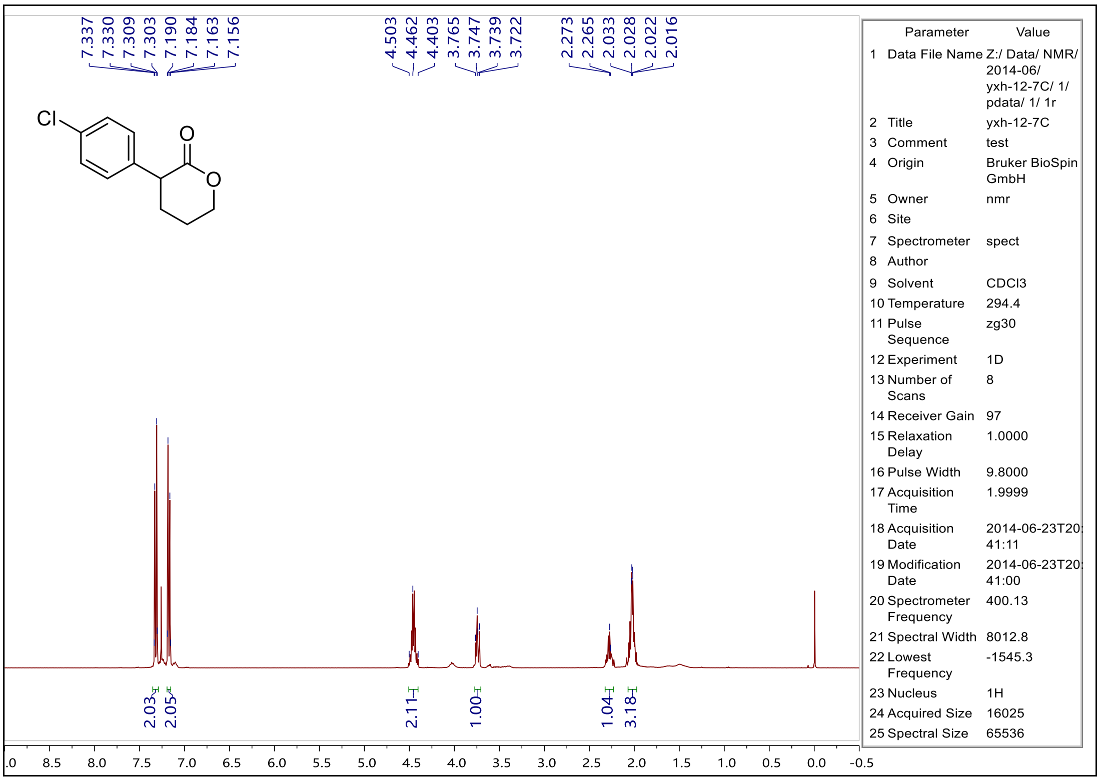

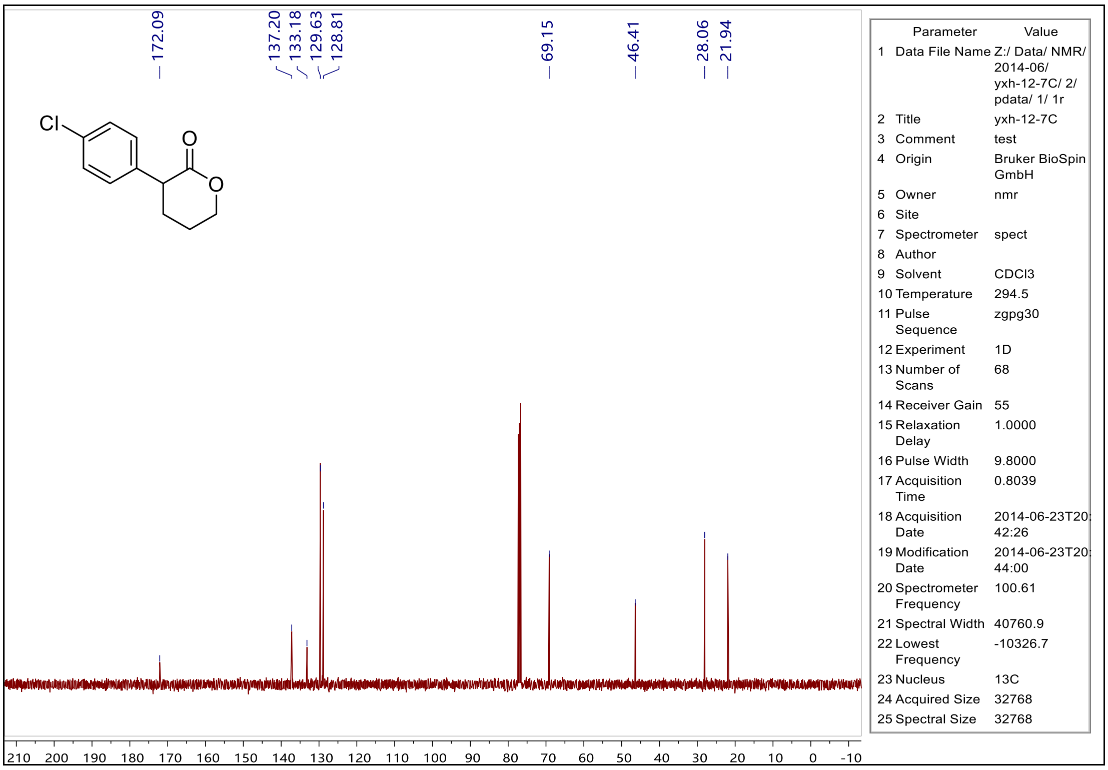

3-(4-Tolyl)tetrahydro-2*H*-pyran-2-one (2c)

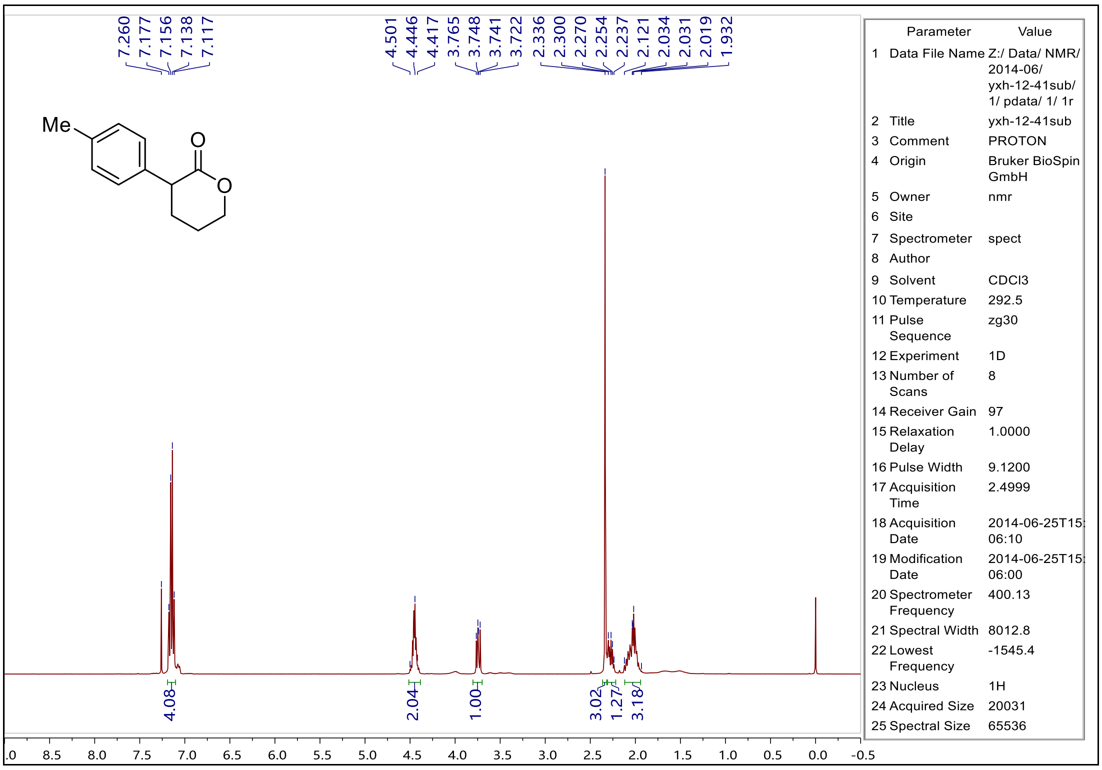

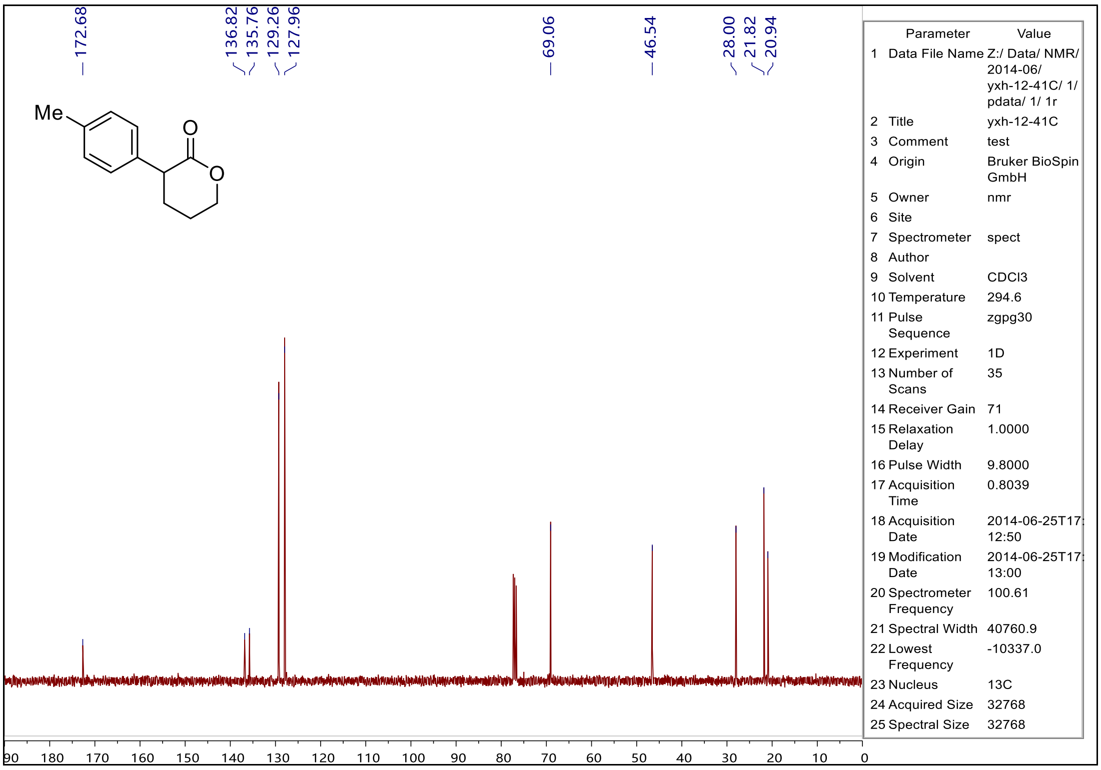

3-(3-Chlorophenyl)tetrahydro-2*H*-pyran-2-one (2e)

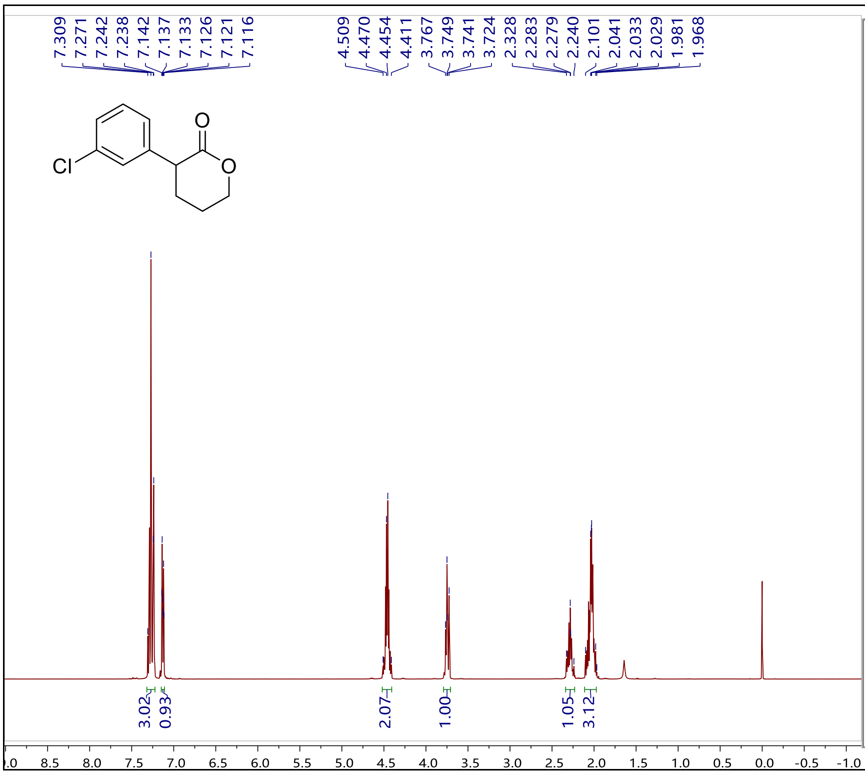

	Parameter	Value
1	Data File Name	Z:/ Data/ NMR/ 2014-07/ yxh-13-11sub/ 1/ pdata/ 1/ 1r
2	Title	yxh-13-11sub
3	Comment	test
4	Origin	Bruker BioSpin GmbH
5	Owner	nmr
6	Site	
7	Spectrometer	spect
8	Author	
9	Solvent	CDCl3
10	Temperature	294.3
11	Pulse Sequence	zg30
12	Experiment	1D
13	Number of Scans	8
14	Receiver Gain	87
15	Relaxation Delay	1.0000
16	Pulse Width	9.8000
17	Acquisition Time	1.9999
18	Acquisition Date	2014-07-17T10: 46:49
19	Modification Date	2014-07-17T10: 47:00
20	Spectrometer Frequency	400.13
21	Spectral Width	8012.8
22	Lowest Frequency	-1543.2
23	Nucleus	1H
24	Acquired Size	16025
25	Spectral Size	65536

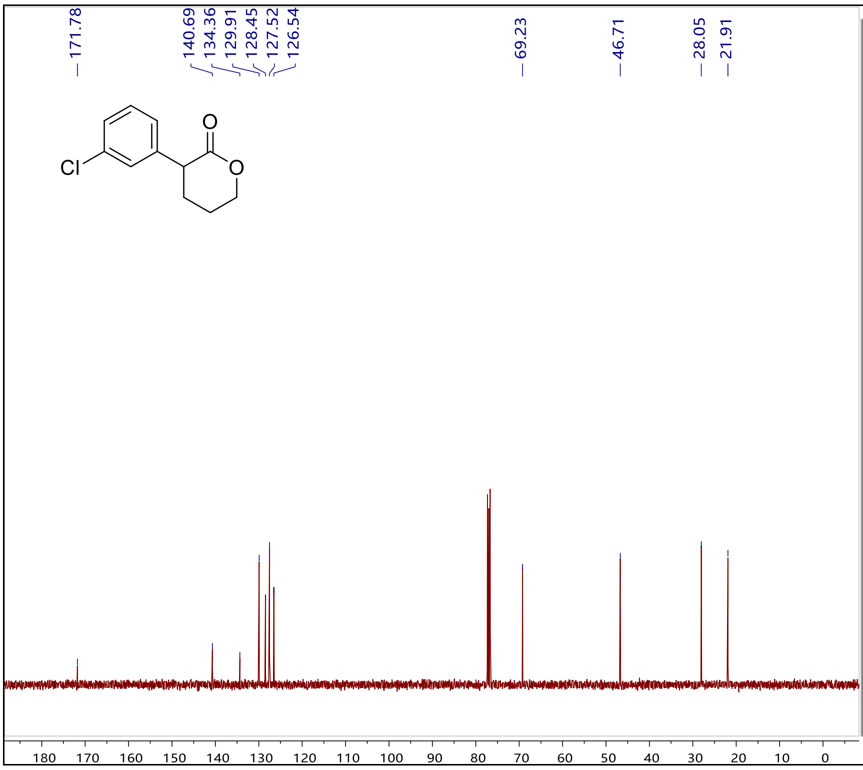

	Parameter	Value
1	Data File Name	Z:/ Data/ NMR/ 2014-07/ yxh-13-11sub/ 2/ pdata/ 1/ 1r
2	Title	yxh-13-11sub
3	Comment	test
4	Origin	Bruker BioSpin GmbH
5	Owner	nmr
6	Site	
7	Spectrometer	spect
8	Author	
9	Solvent	CDCl3
10	Temperature	294.5
11	Pulse Sequence	zgpg30
12	Experiment	1D
13	Number of Scans	43
14	Receiver Gain	97
15	Relaxation Delay	1.0000
16	Pulse Width	9.8000
17	Acquisition Time	0.8039
18	Acquisition Date	2014-07-17T10: 48:17
19	Modification Date	2014-07-17T10: 49:00
20	Spectrometer Frequency	100.61
21	Spectral Width	40760.9
22	Lowest Frequency	-10327.8
23	Nucleus	13C
24	Acquired Size	32768
25	Spectral Size	32768

3-(3,4-Dichlorophenyl)tetrahydro-2*H*-pyran-2-one (2h)

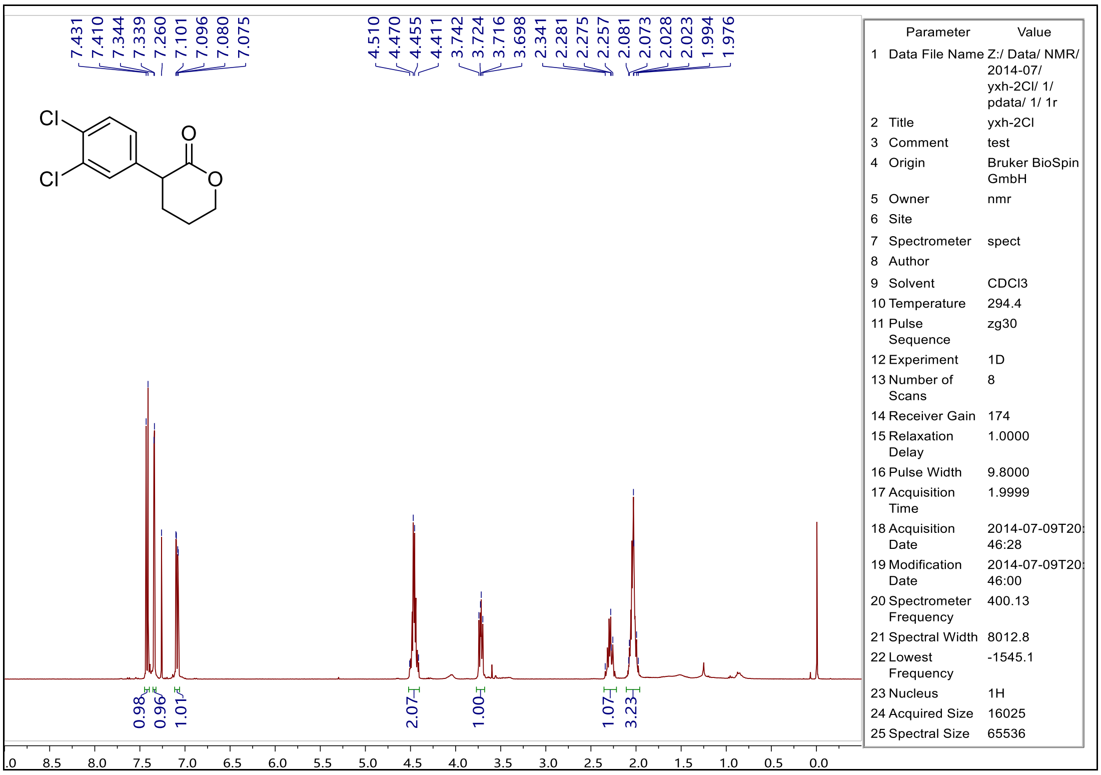

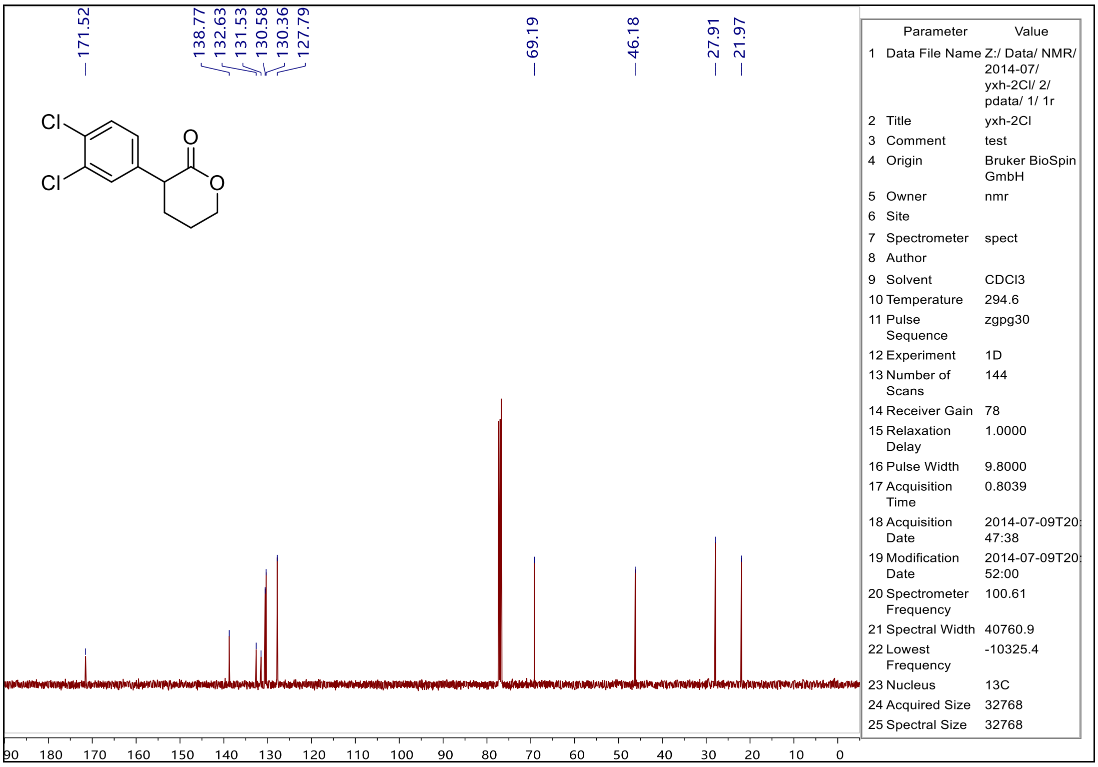

3-(3,4-Dimethoxyphenyl)tetrahydro-2*H*-pyran-2-one (2i)

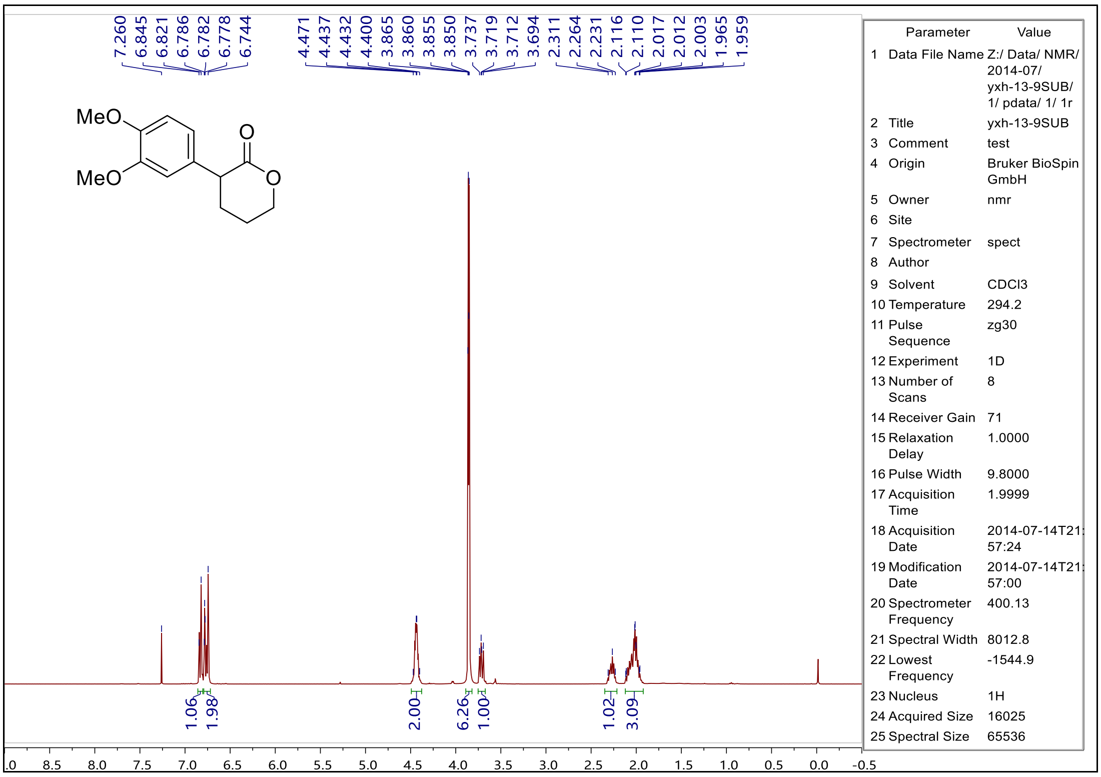

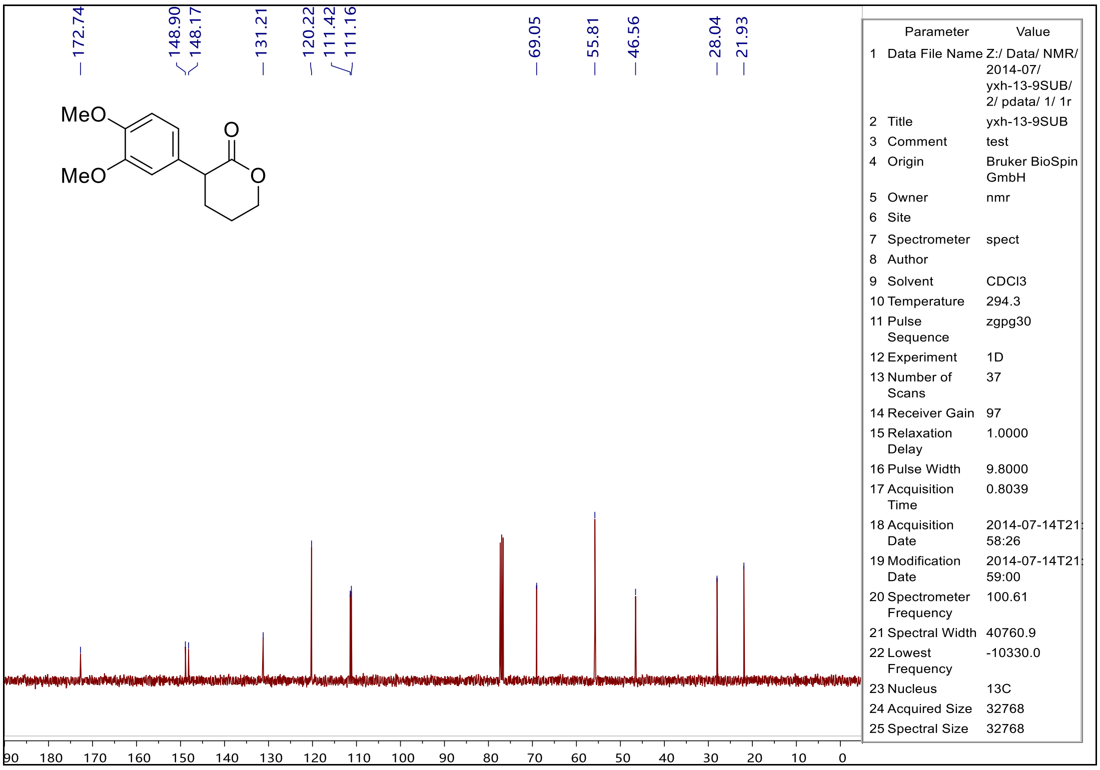

3-(2-Chlorophenyl)tetrahydro-2*H*-pyran-2-one (2j)

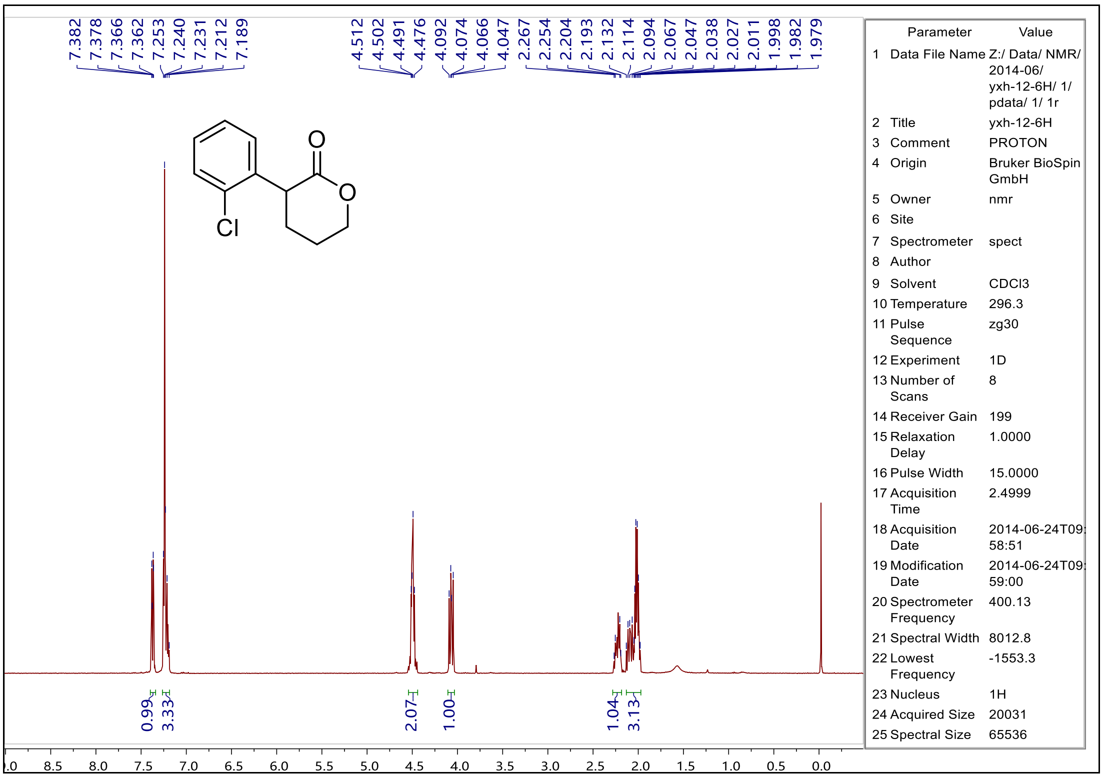

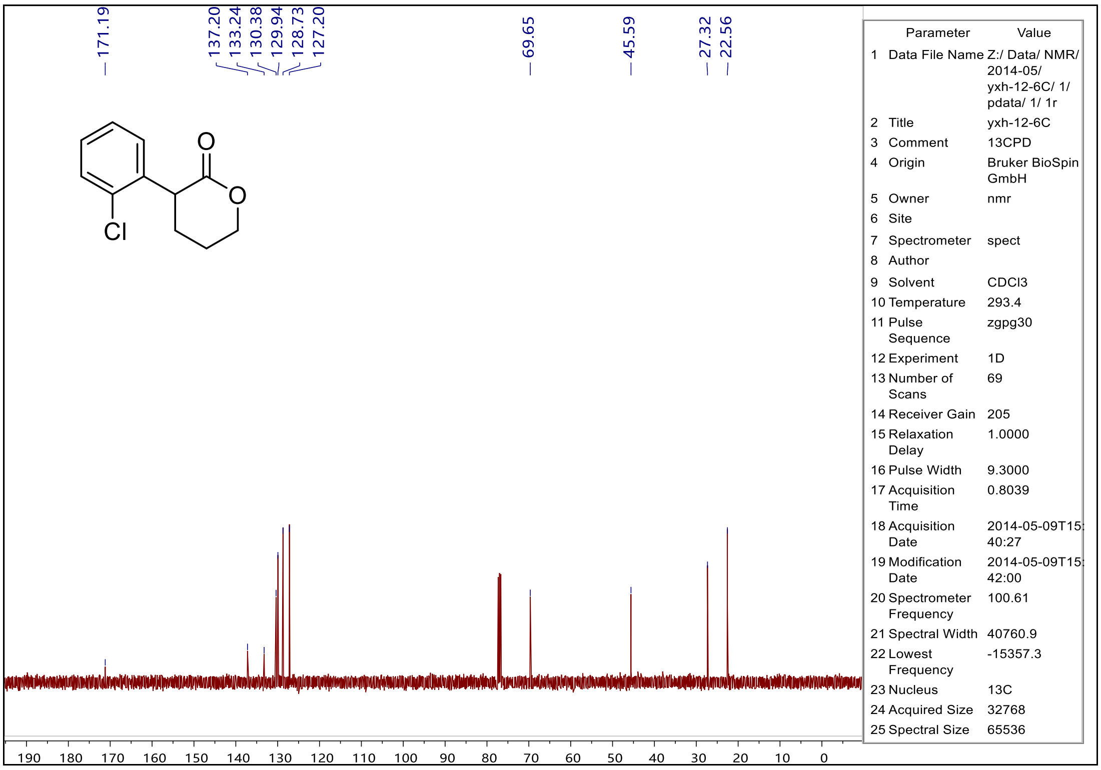

3-(2-Tolyl)tetrahydro-2*H*-pyran-2-one (2k)

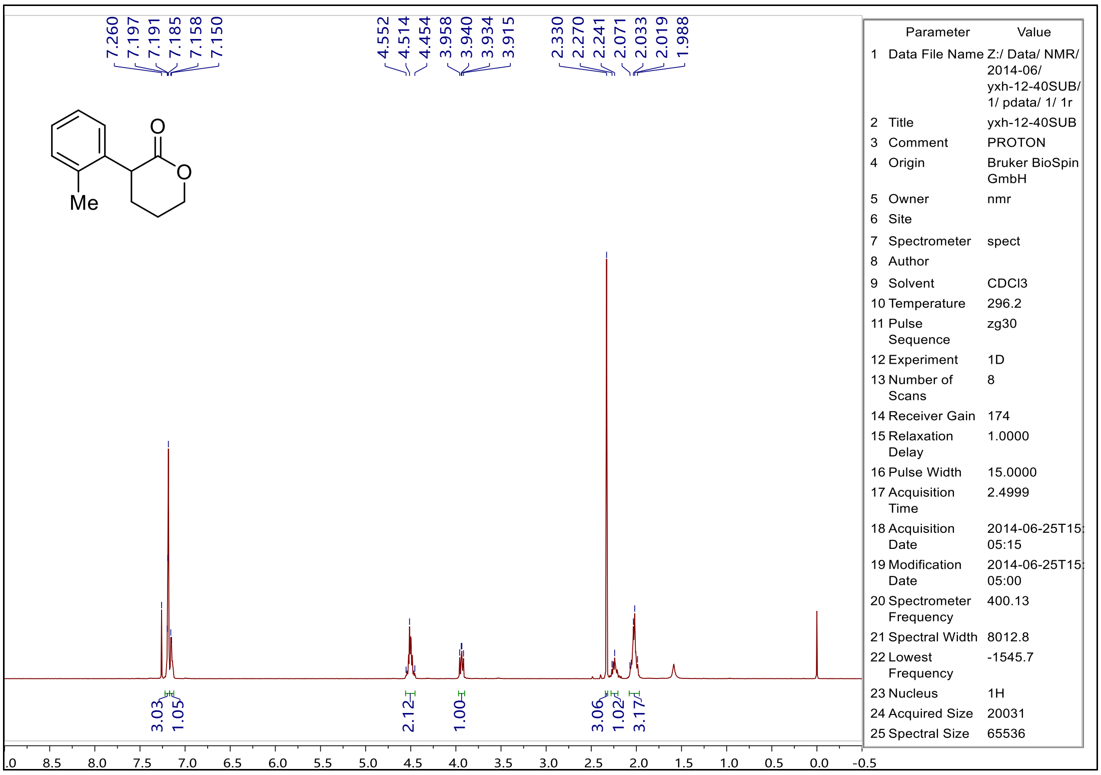

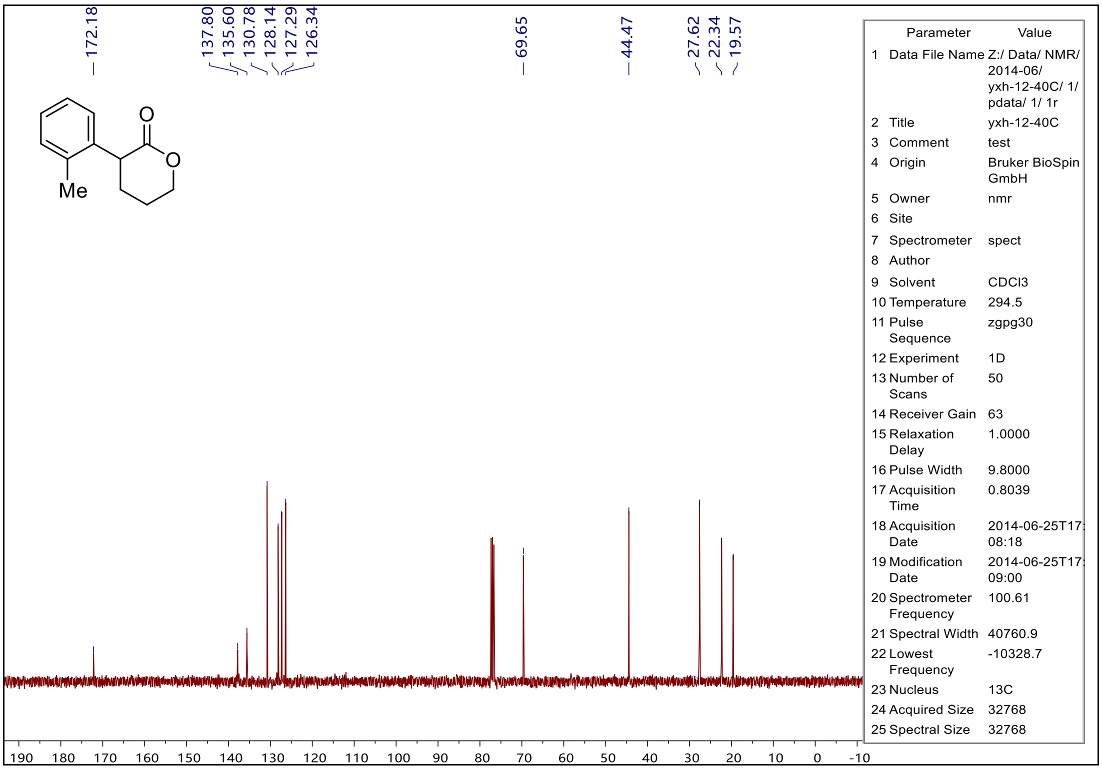

3-(2-Methoxyphenyl)tetrahydro-2*H*-pyran-2-one (2l)

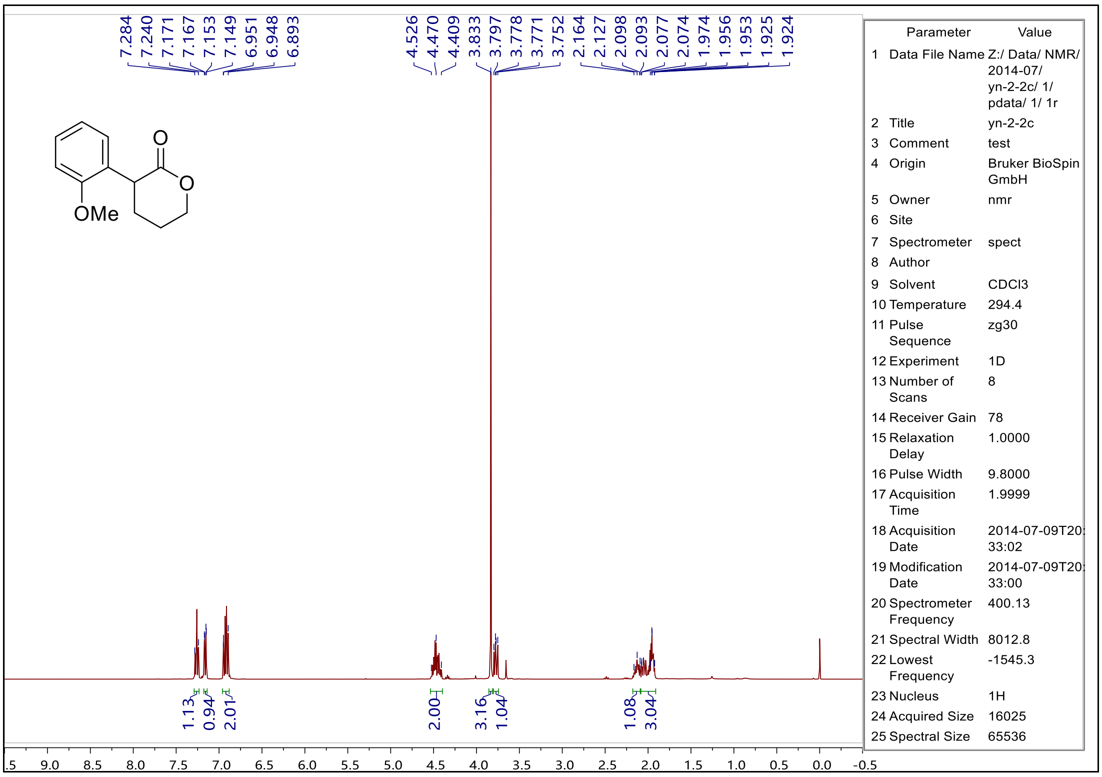

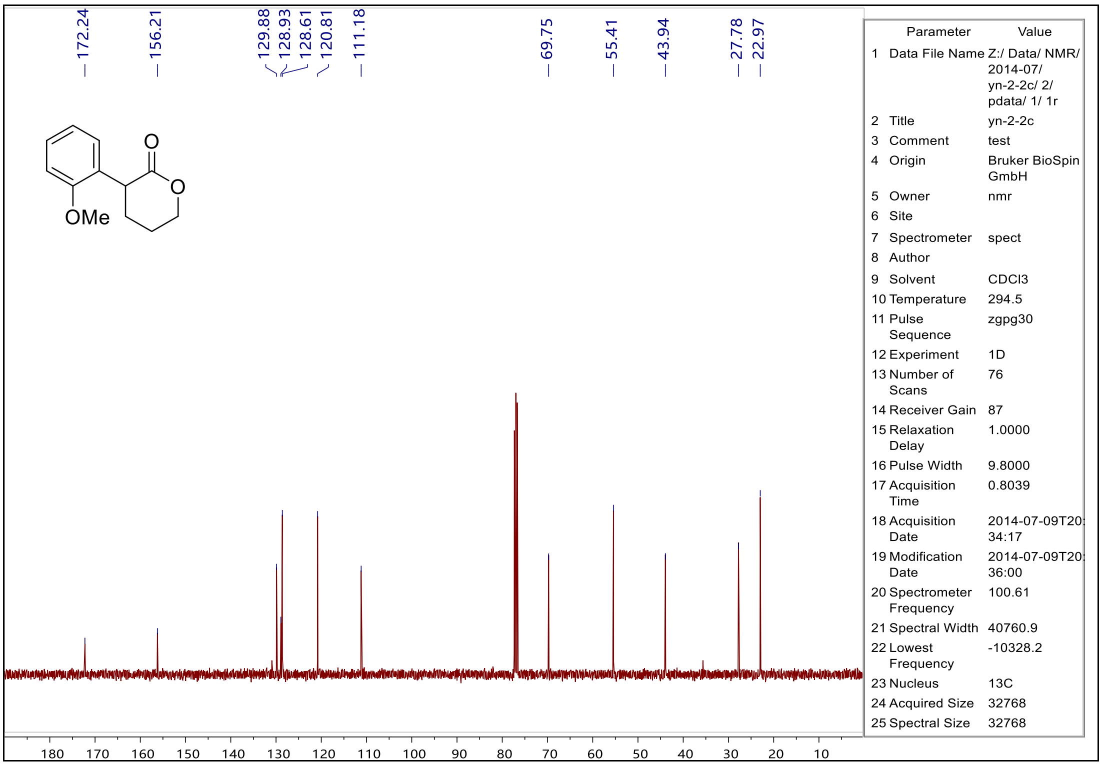

3-(But-3-en-1-yl)tetrahydro-2*H*-pyran-2-one (2p)

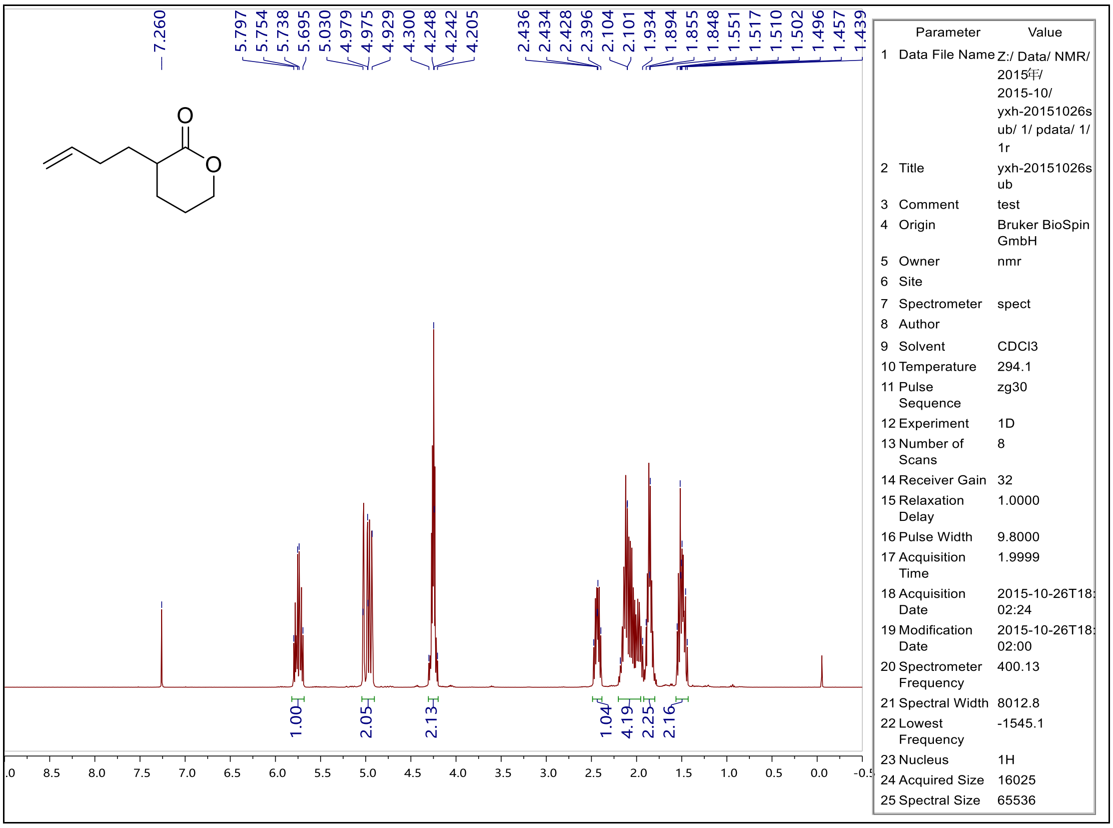

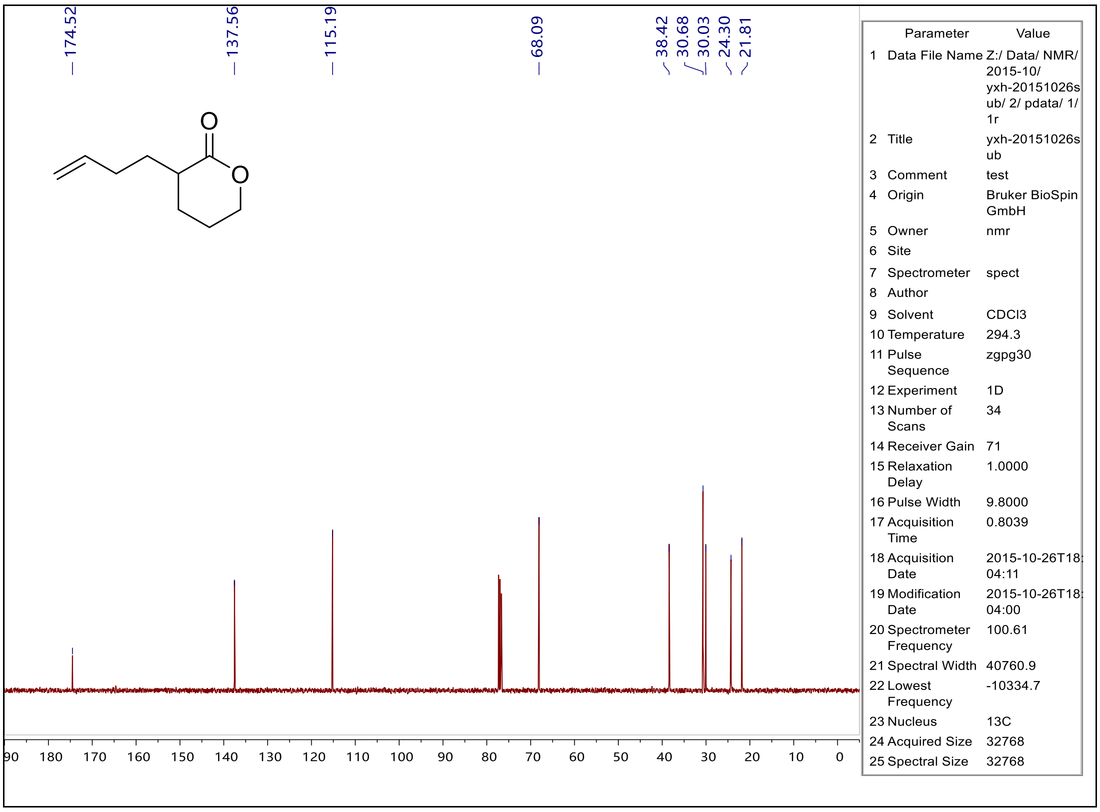

(*R*)-2-(4-Chlorophenyl)pentane-1,5-diol (3b)

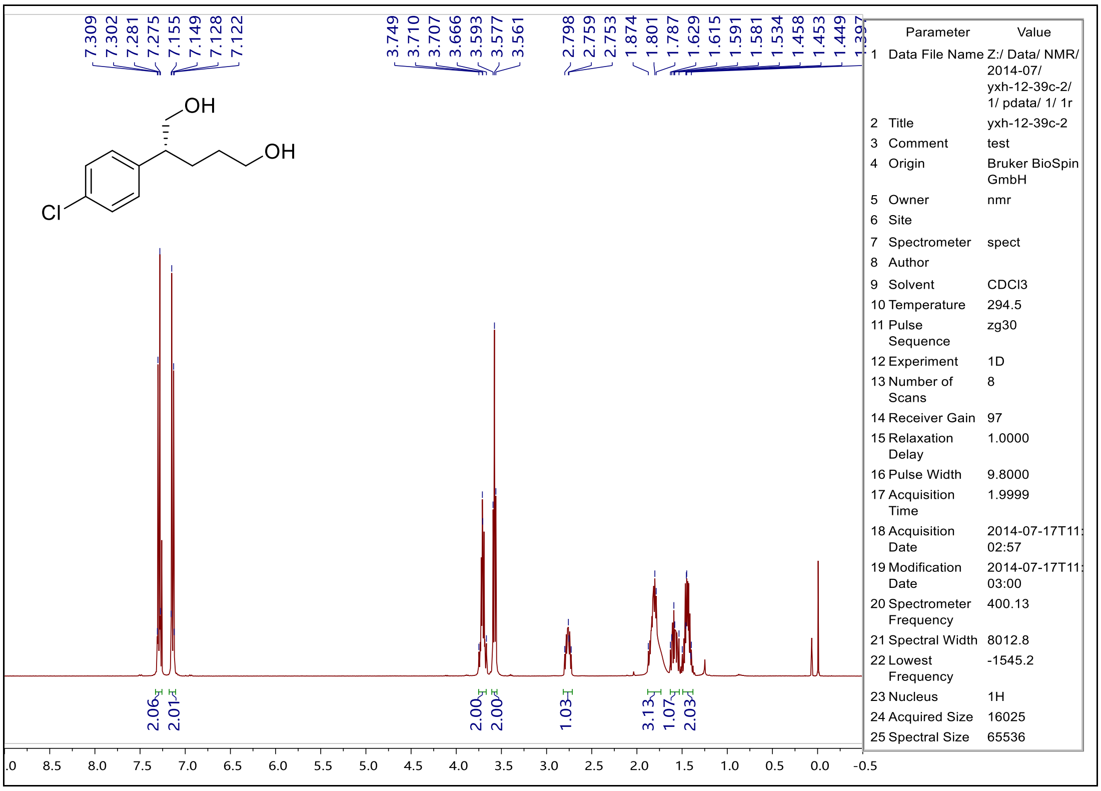

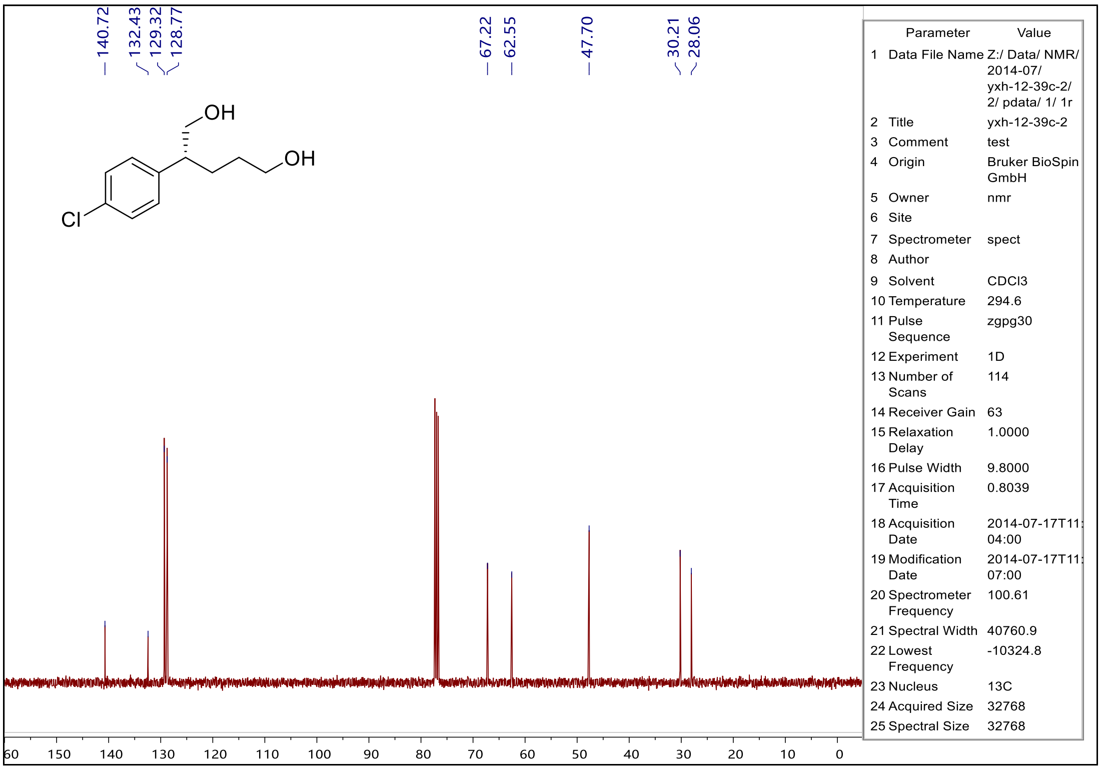

(*R*)-2-(4-Tolyl)pentane-1,5-diol (3c)

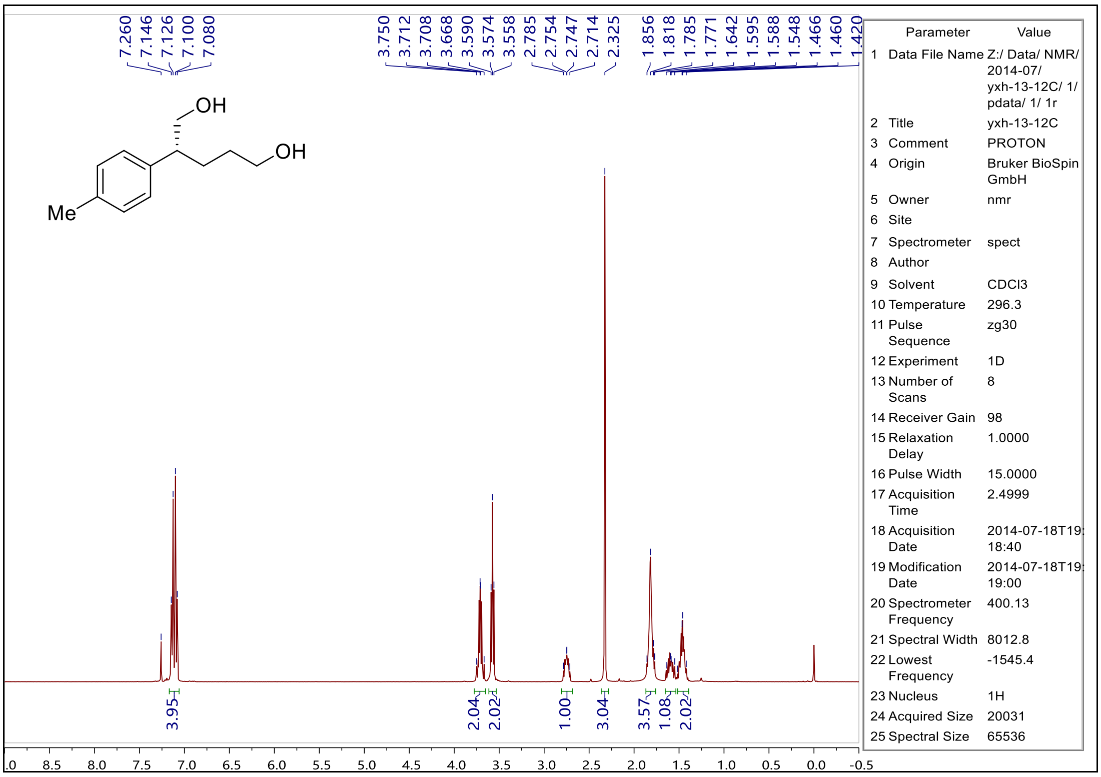

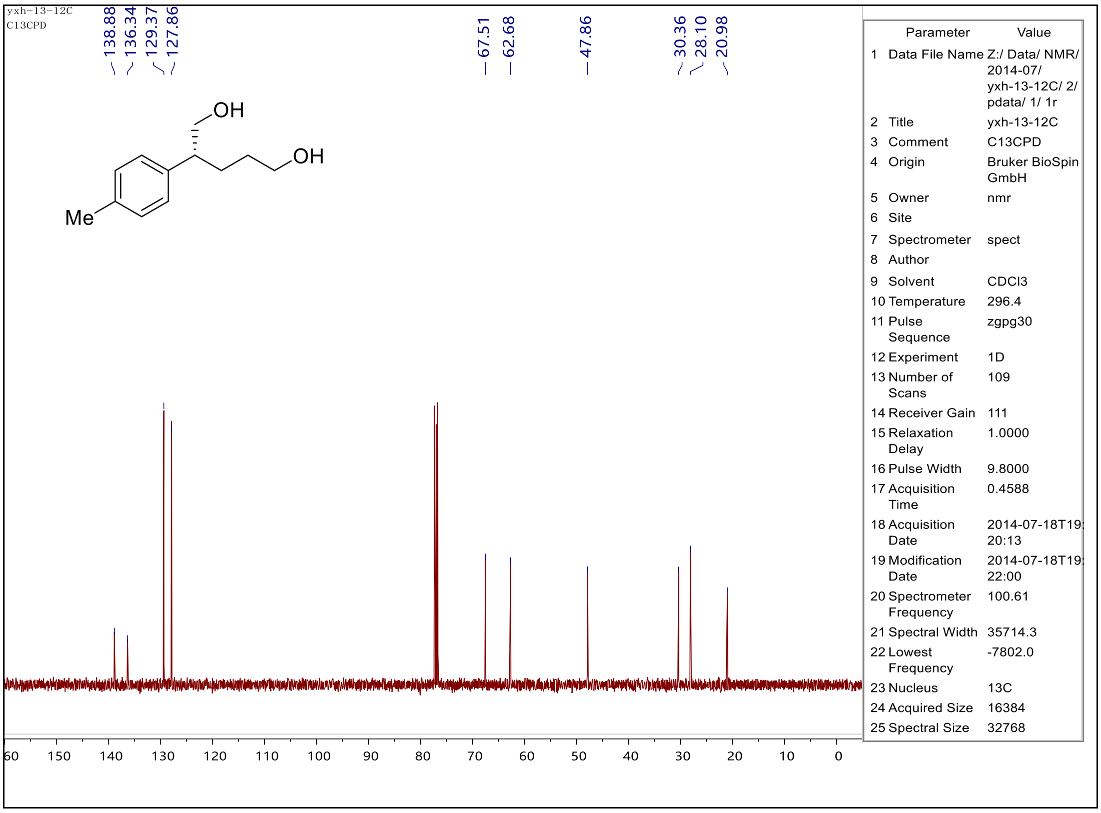

(*R*)-2-(3-Chlorophenyl)pentane-1,5-diol (3e)

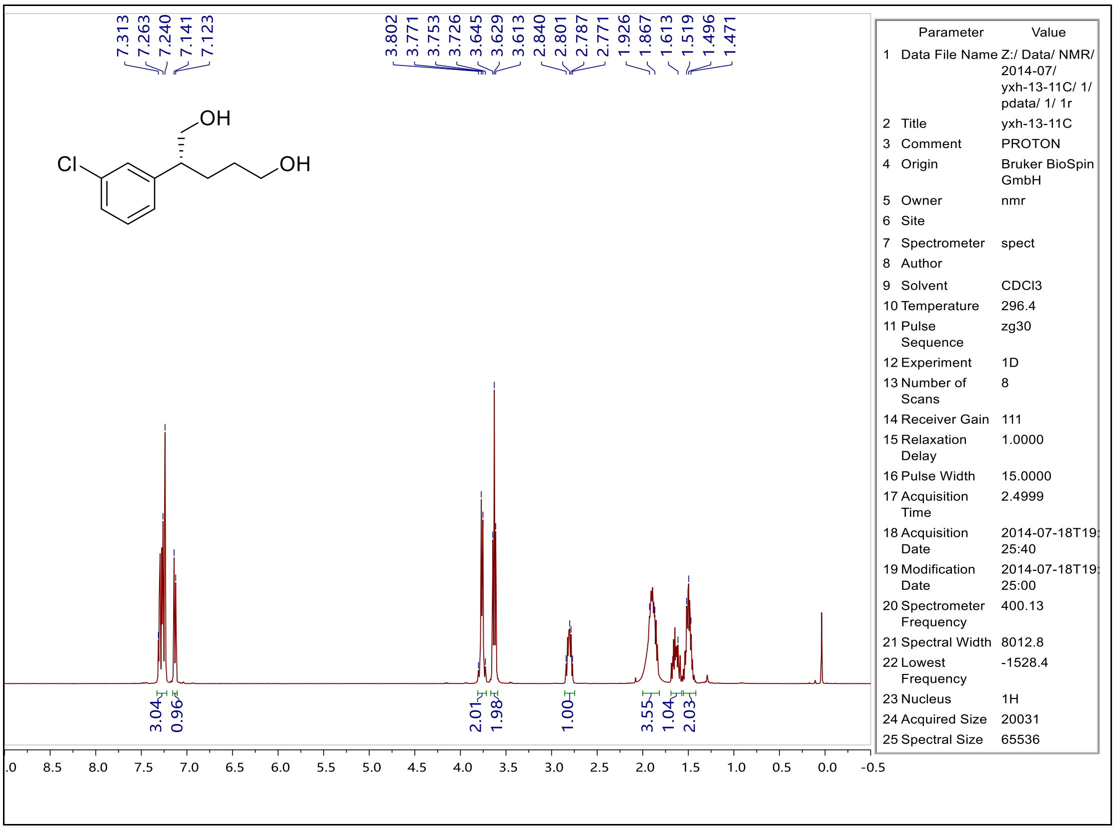

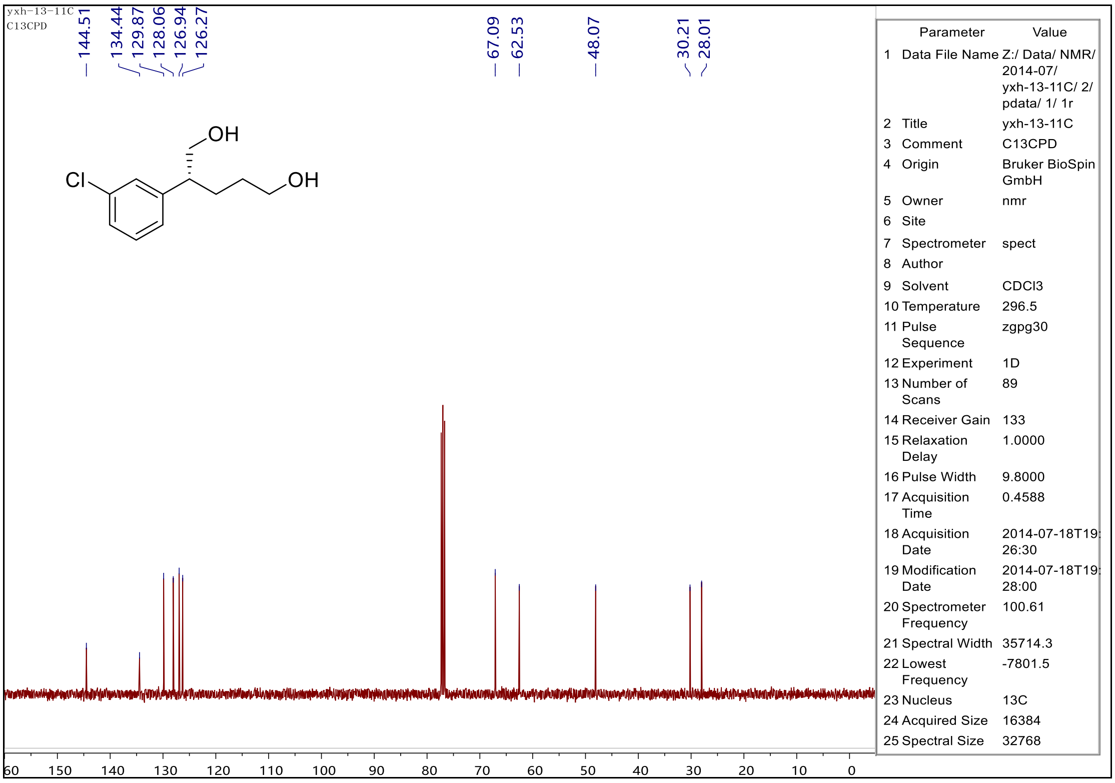

(*R*)-2-(3-Tolyl)pentane-1,5-diol (3f)

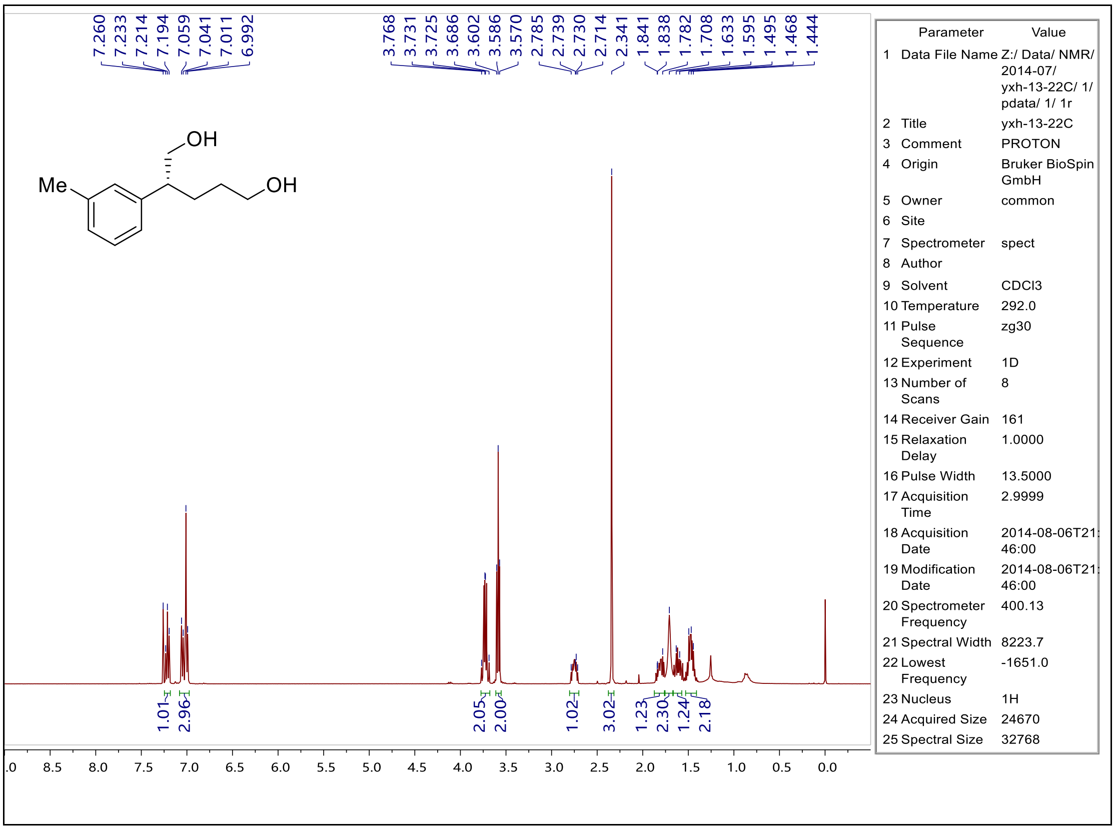

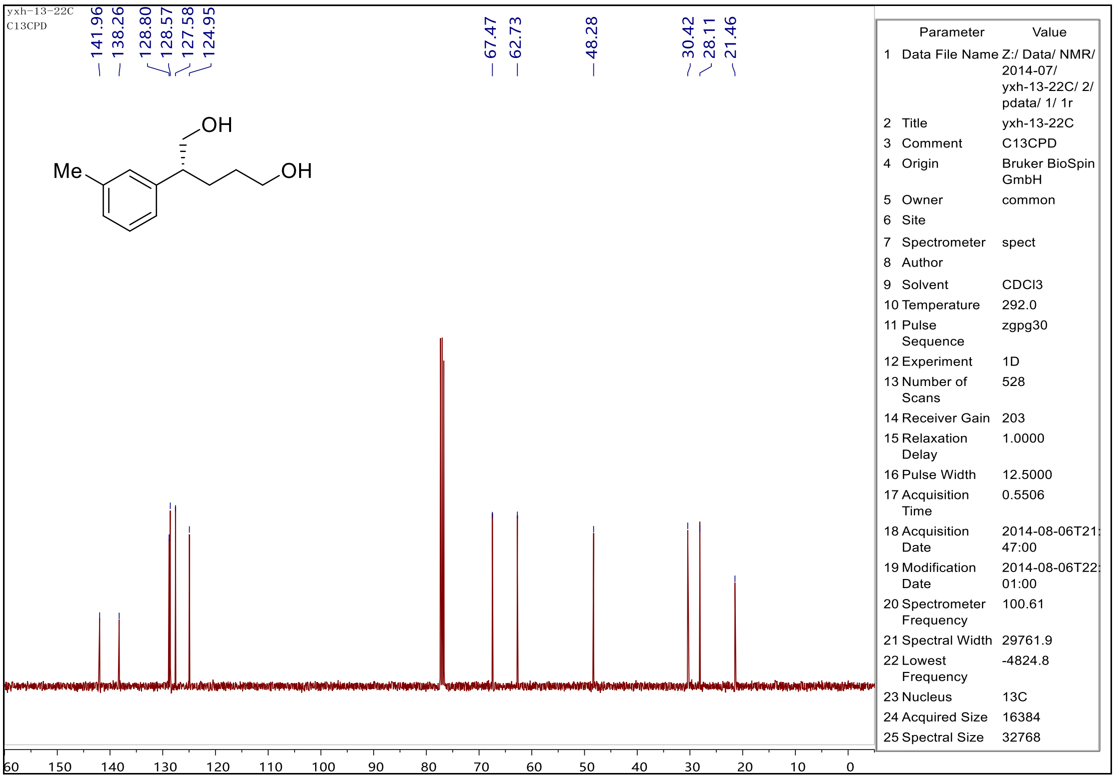

(*R*)-2-(3-Methoxyphenyl)pentane-1,5-diol (3g)

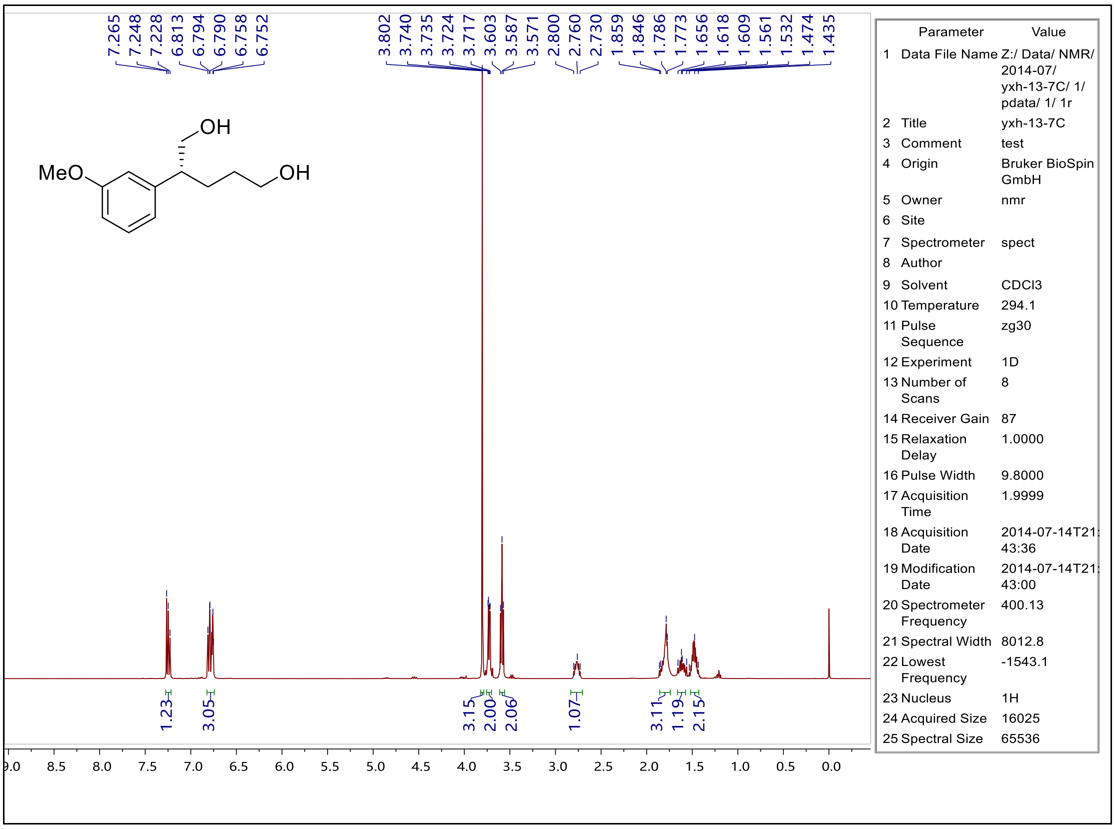

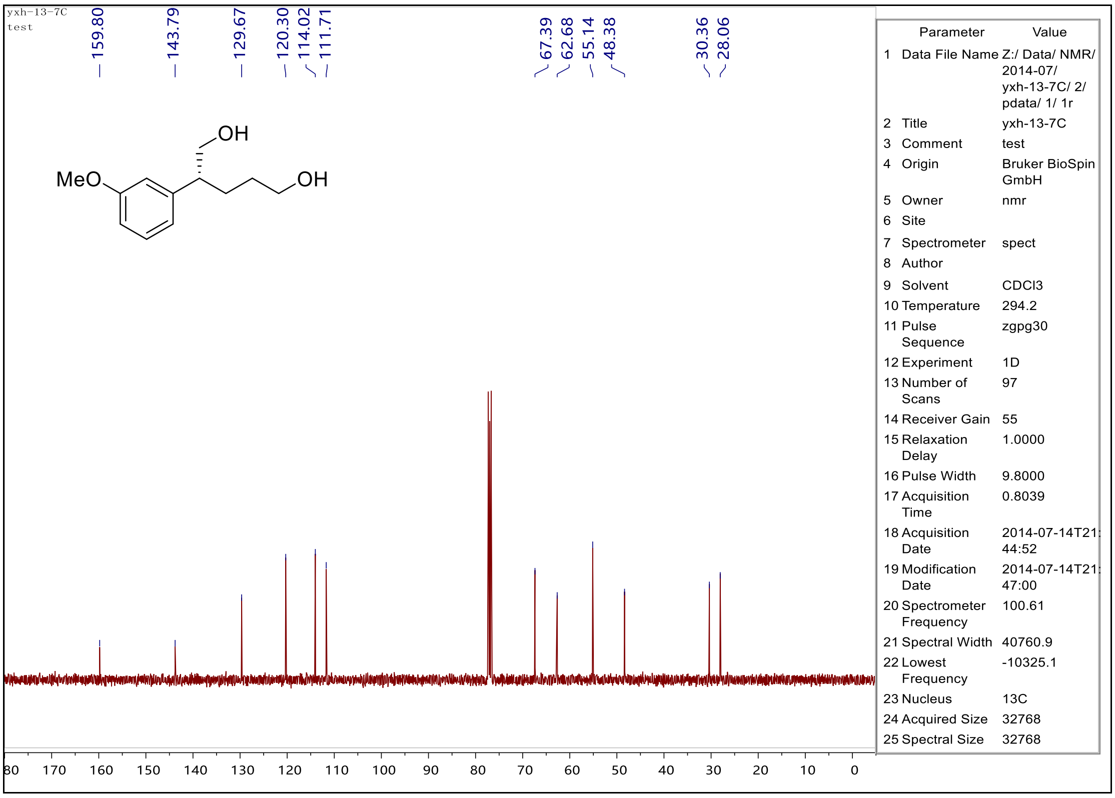

(*R*)-2-(3,4-Dichlorophenyl)pentane-1,5-diol (3h)

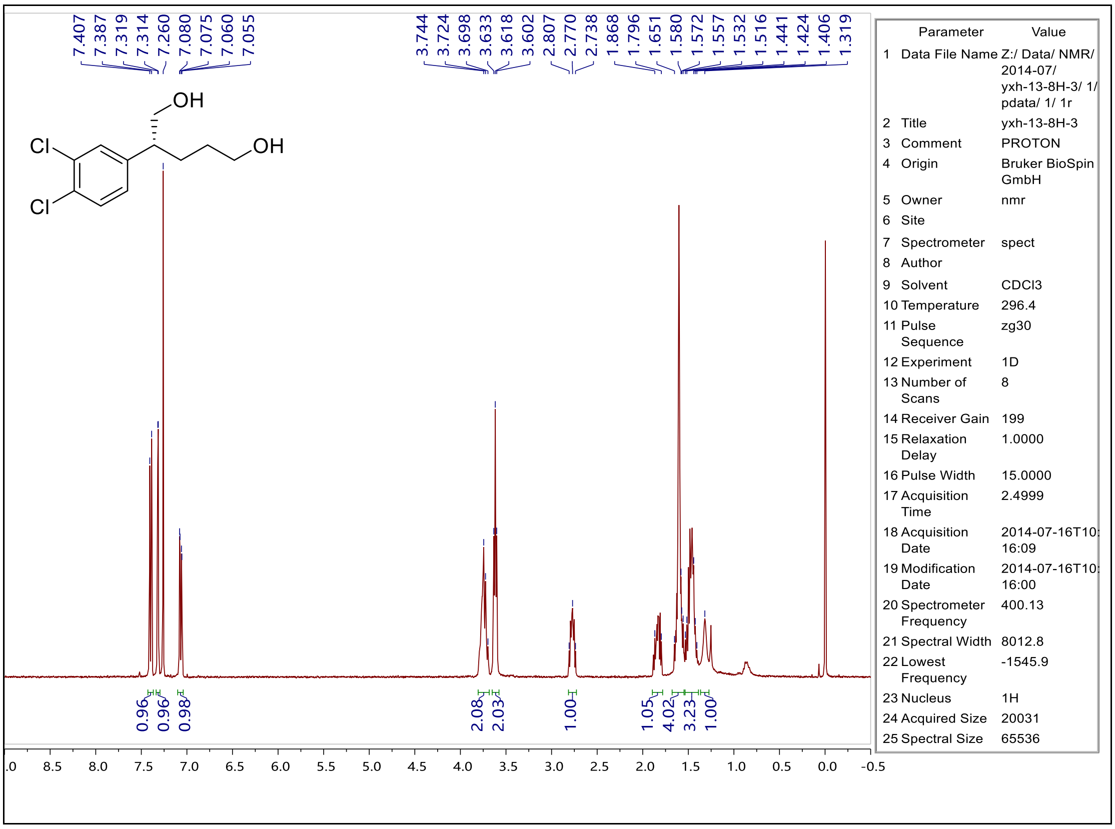

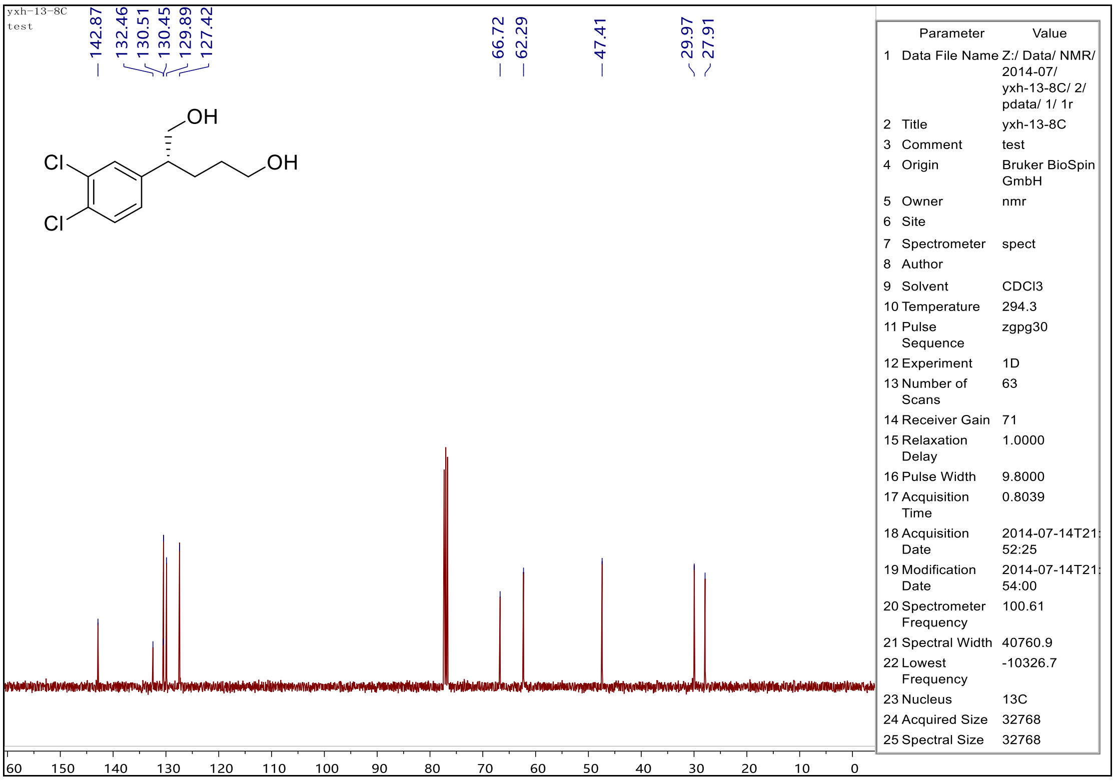

(*R*)-2-(3,4-Dimethoxyphenyl)pentane-1,5-diol (3i)

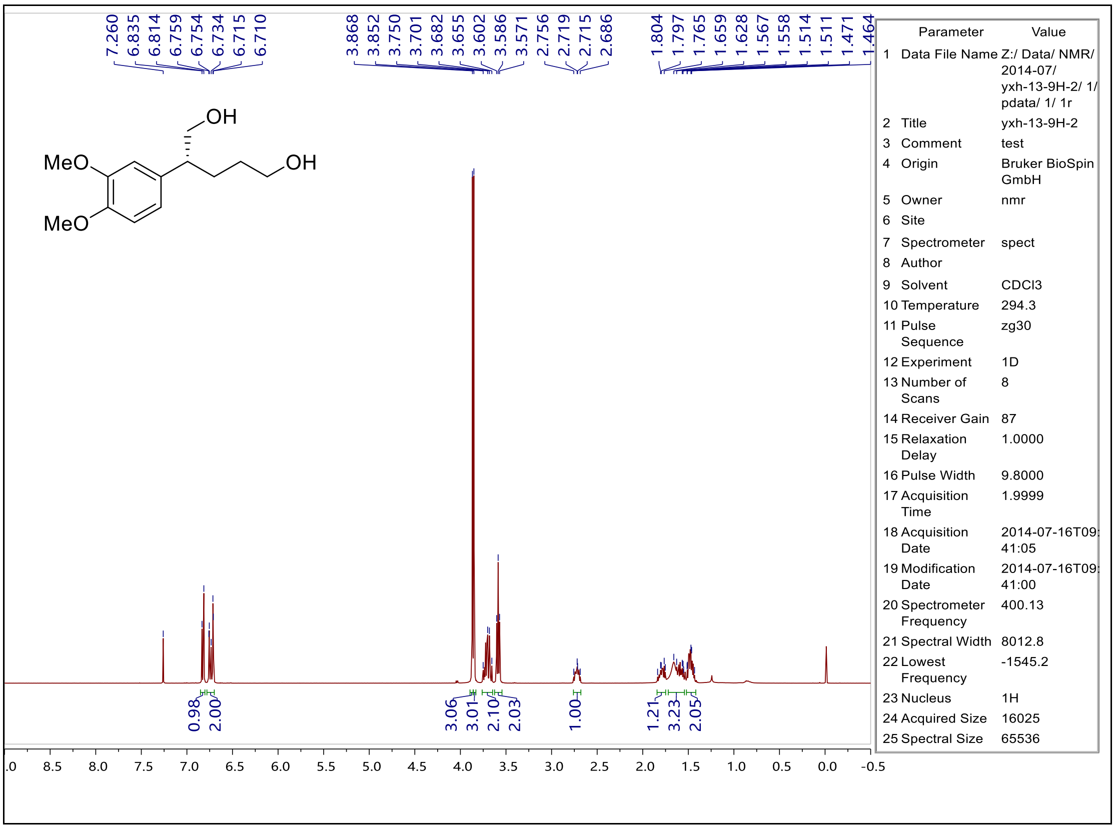

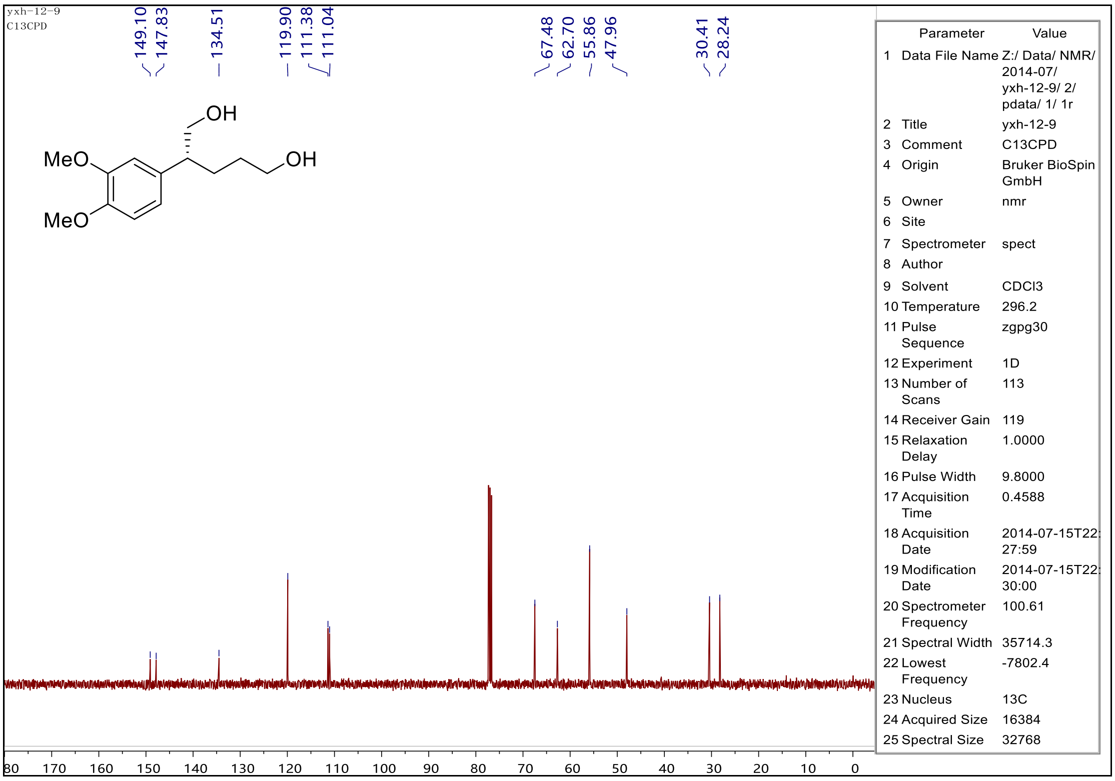

(*R*)-2-(2-Chlorophenyl)pentane-1,5-diol (3j)

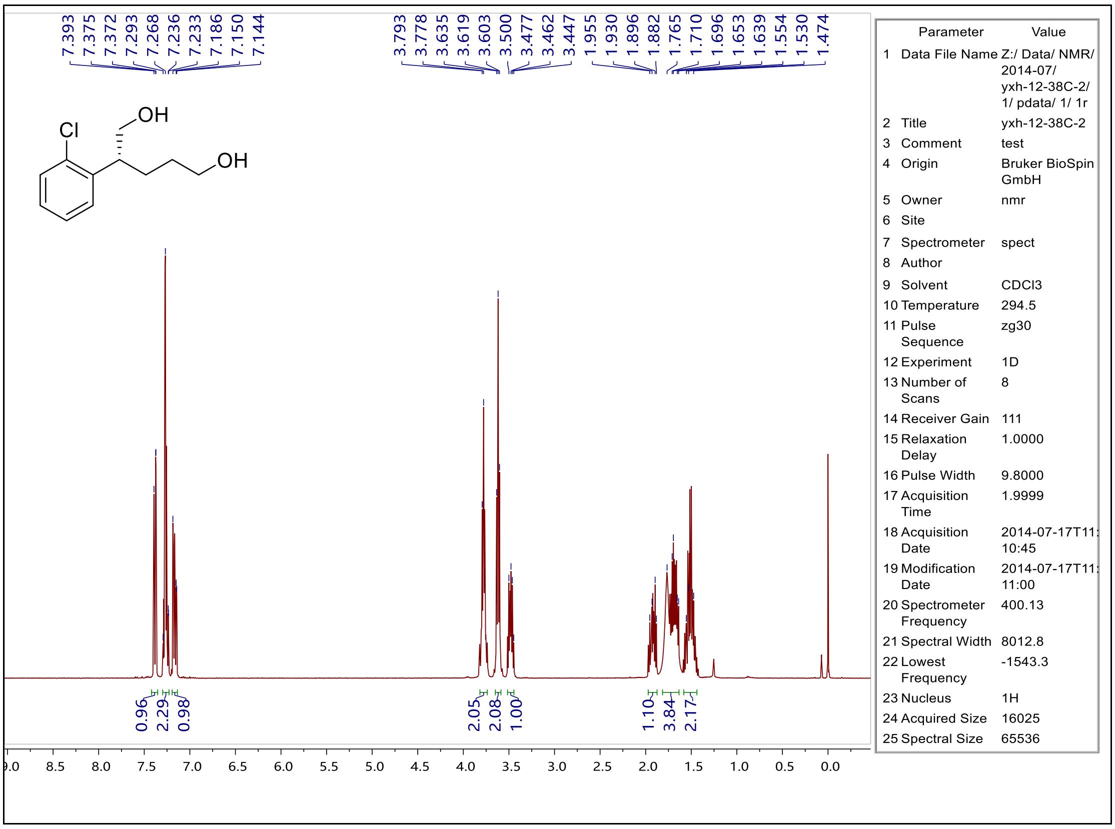

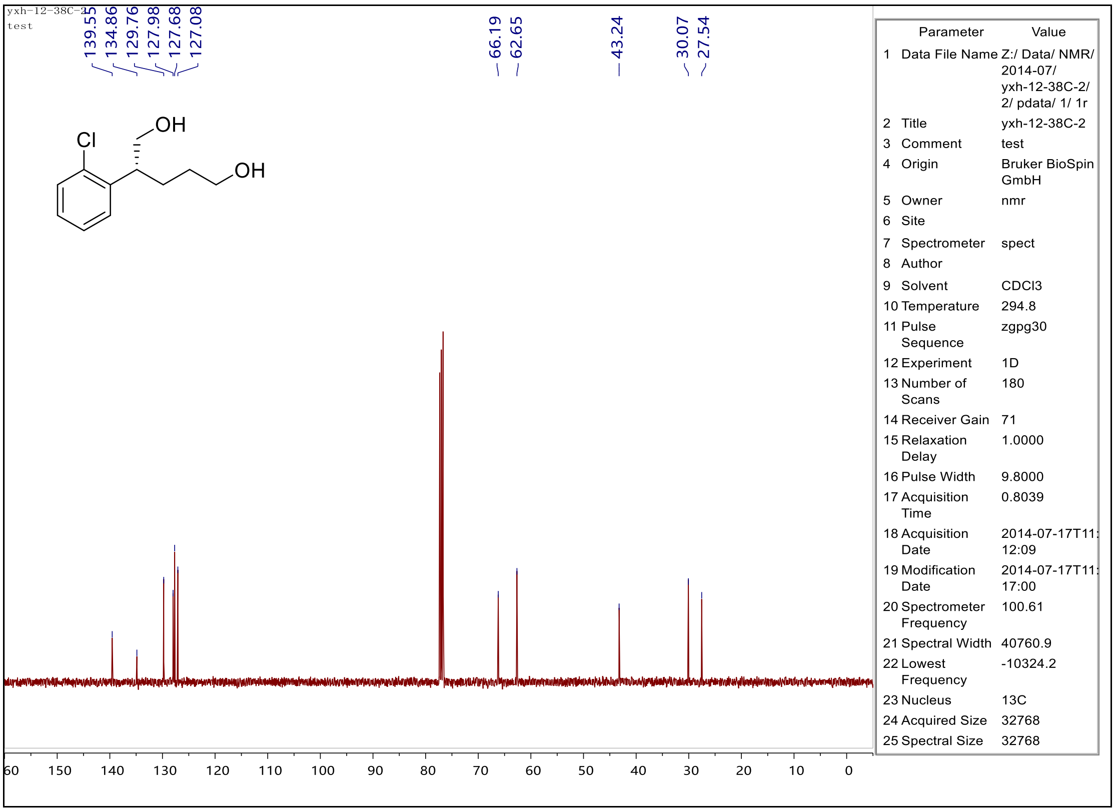

(*R*)-2-(2-Tolyl)pentane-1,5-diol (3k)

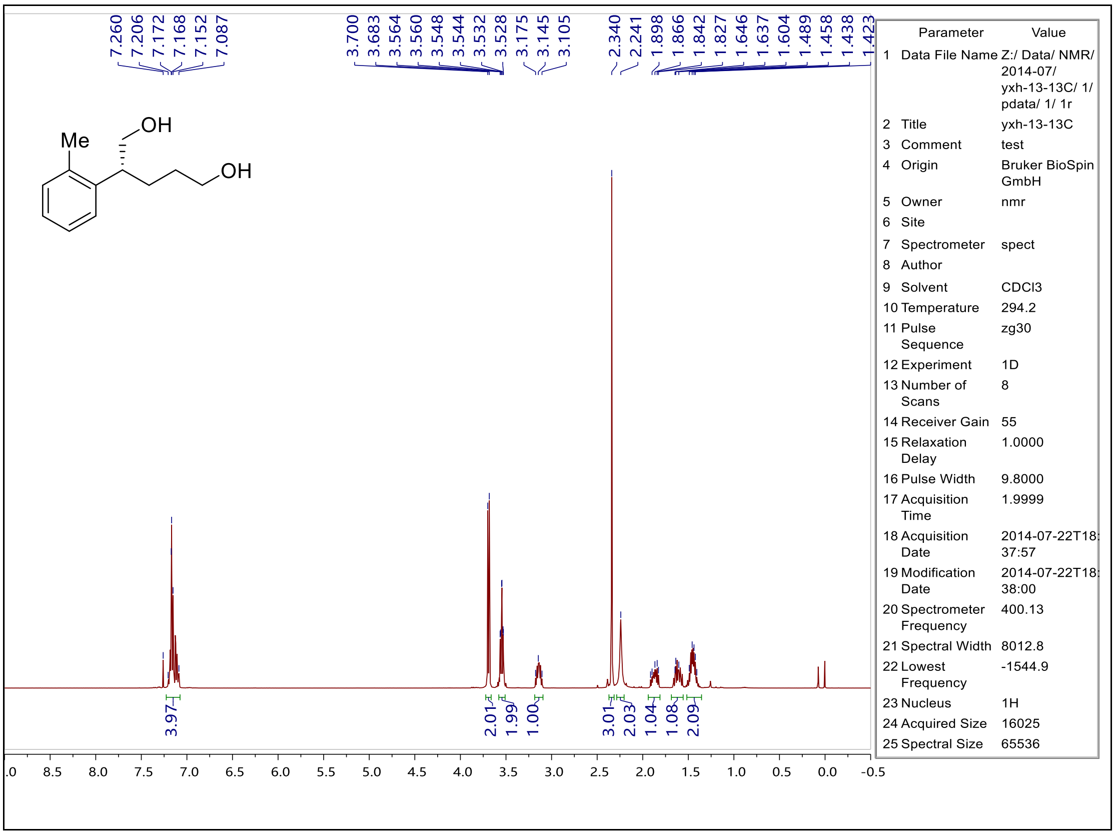

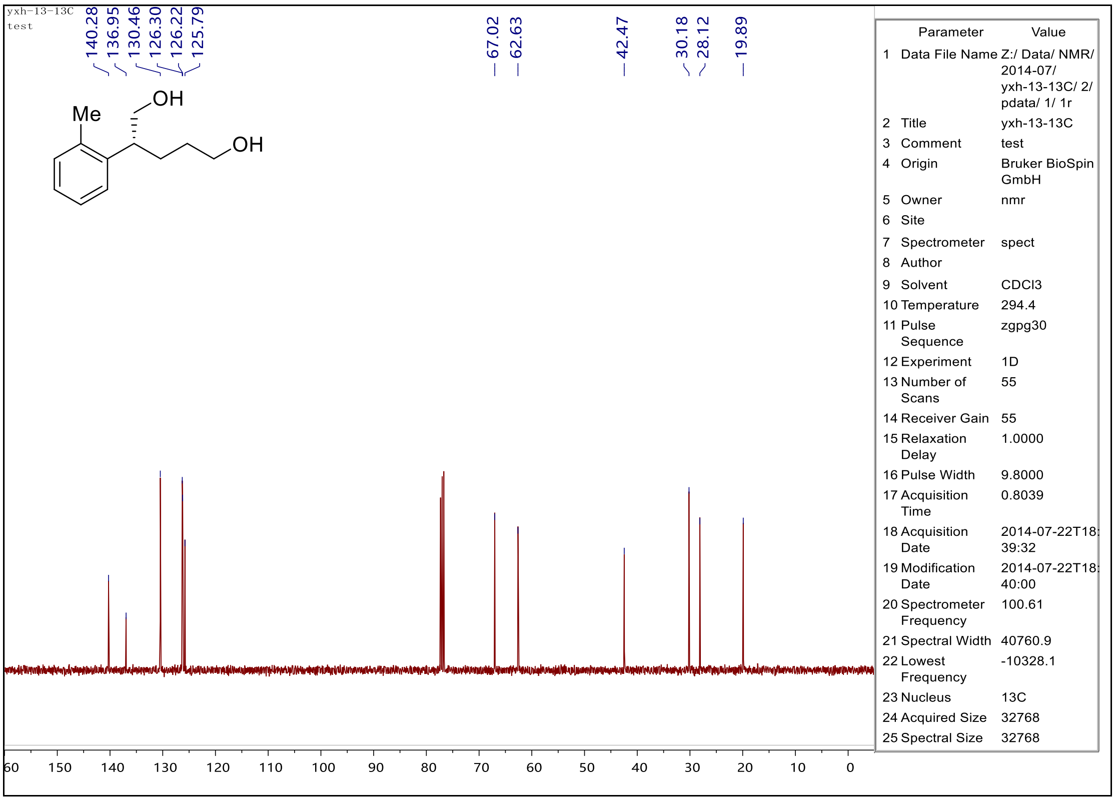

(*R*)-2-(2-Methoxyphenyl)pentane-1,5-diol (3l)

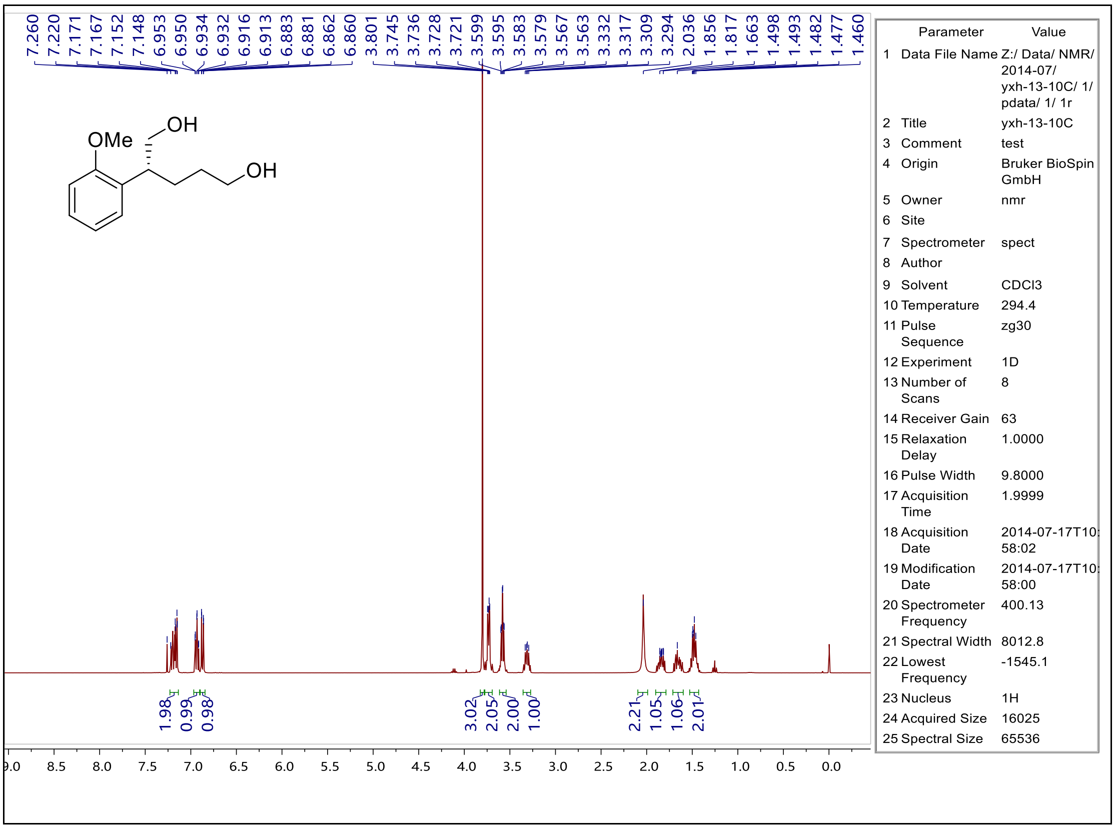

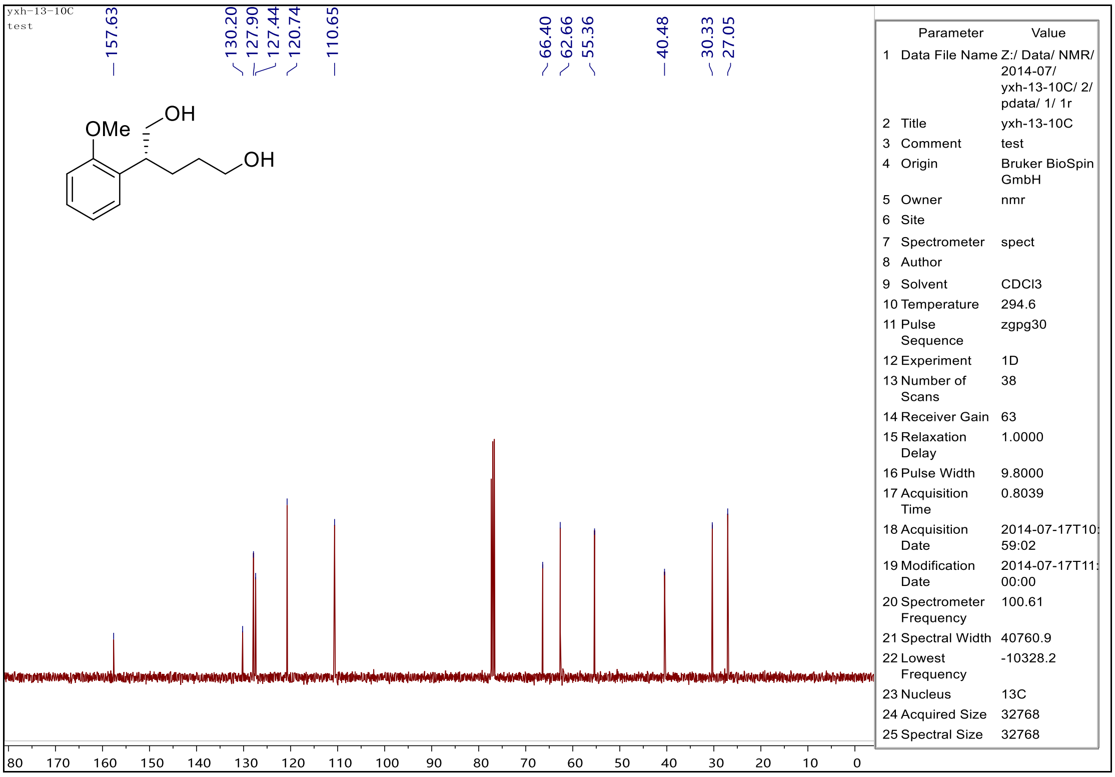

(*S*)-2-(But-3-en-1-yl)pentane-1,5-diol (3p)

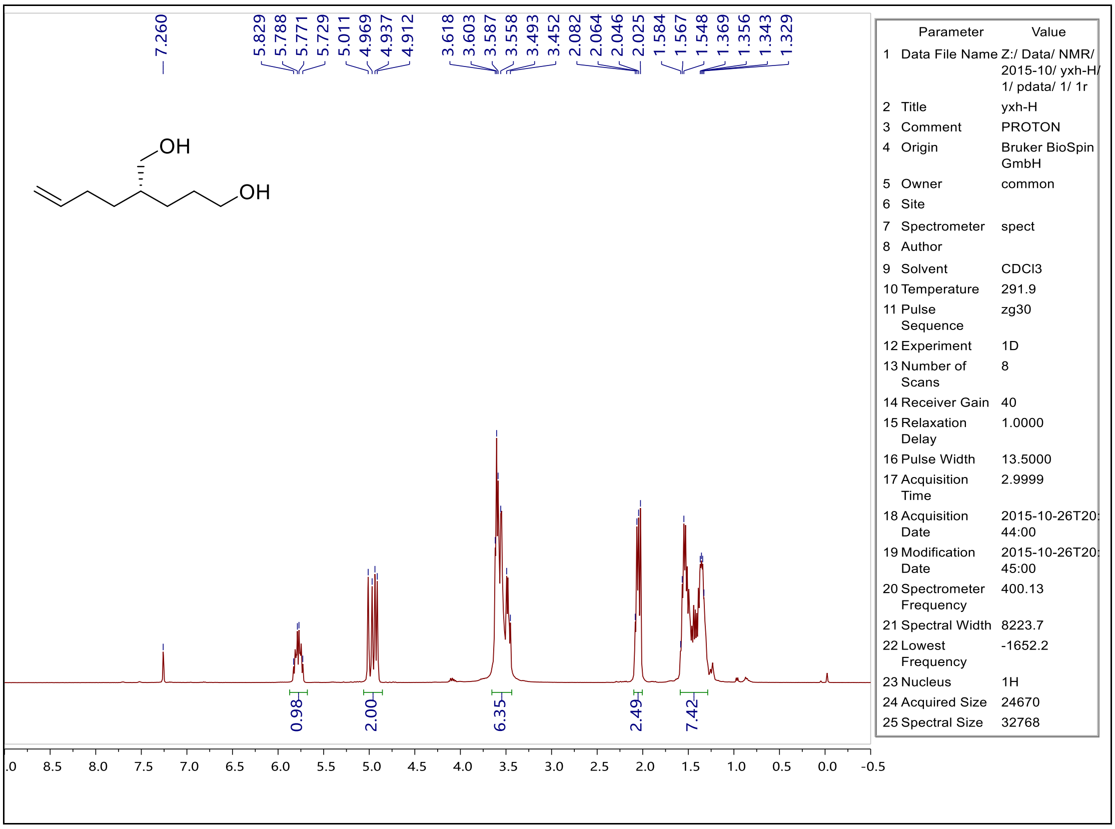

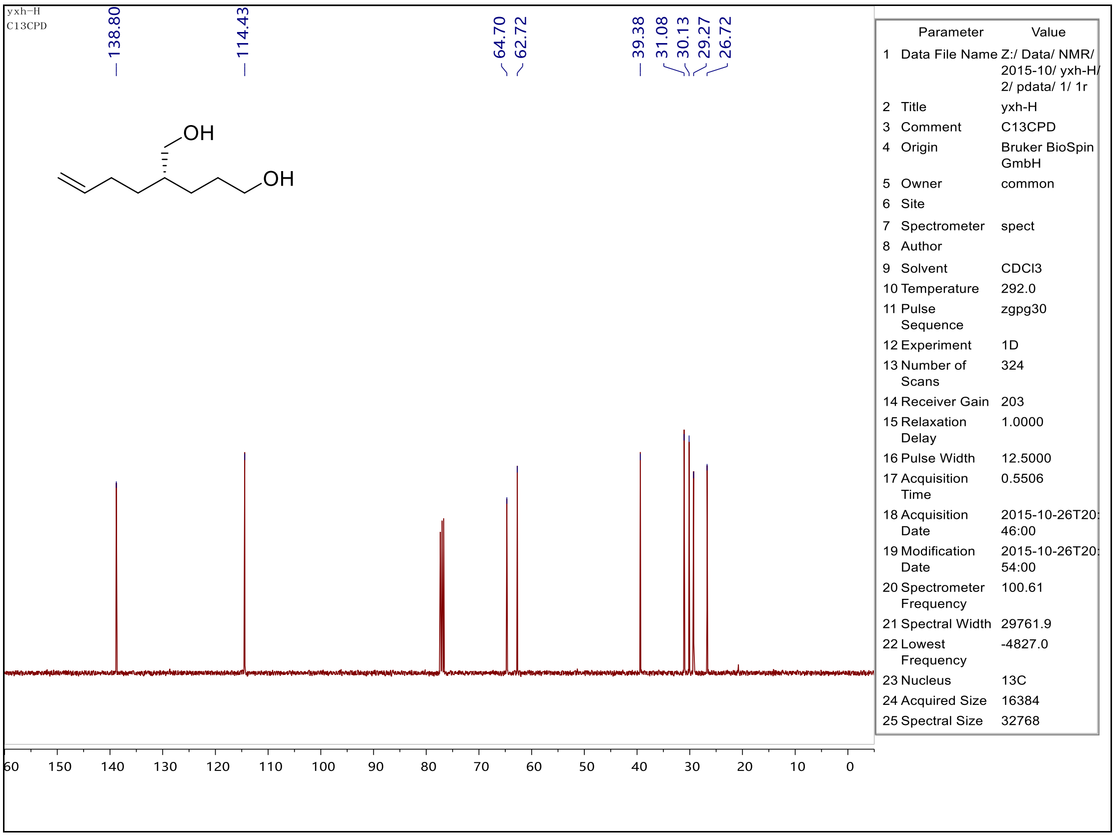

Compound 4

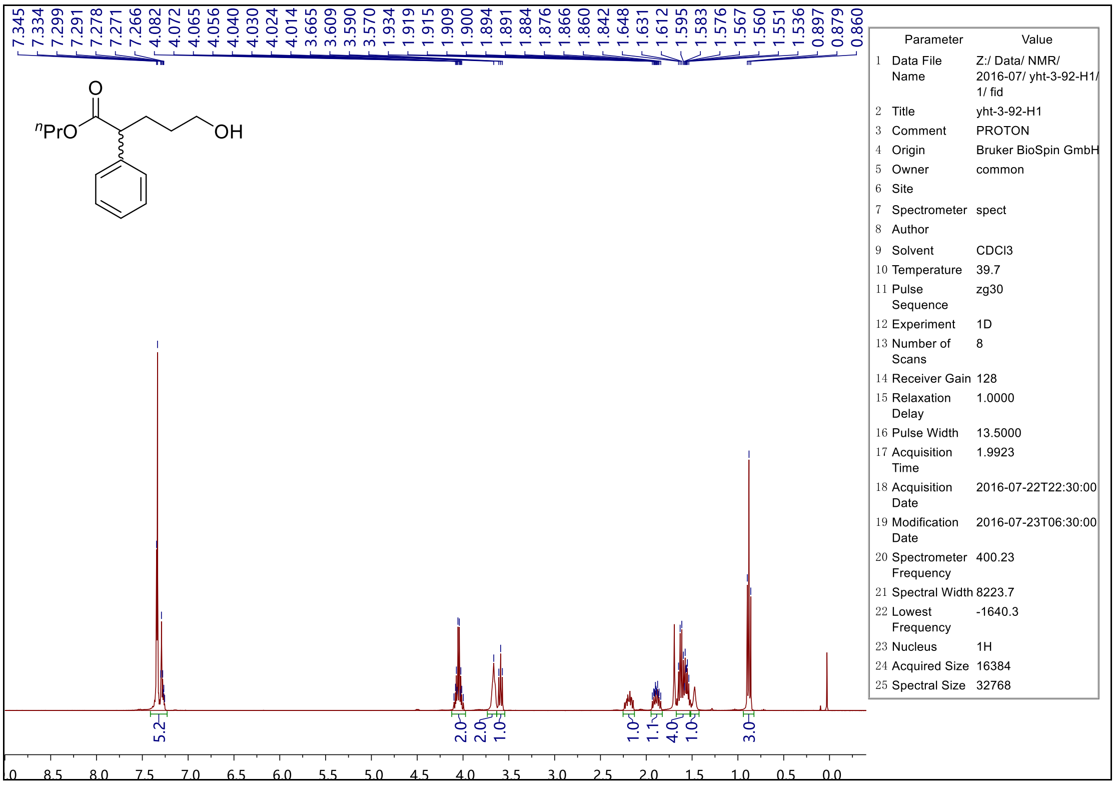

7.345 7.334 7.299 7.291 7.278 7.271 7.266 4.082 4.072 4.065 4.056 4.040 4.030 4.024 4.014 3.665 3.609 3.590 3.570 1.934 1.919 1.915 1.909 1.900 1.894 1.891 1.884 1.876 1.866 1.860 1.842 1.648 1.631 1.612 1.595 1.583 1.576 1.567 1.560 1.551 1.536 0.897 0.879 0.860
O
^{n}PrO
OH
5.2
2.0
2.0
1.0
1.0
1.1
4.0
1.0
3.0
8.5 8.0 7.5 7.0 6.5 6.0 5.5 5.0 4.5 4.0 3.5 3.0 2.5 2.0 1.5 1.0 0.5 0.0
Parameter Value
1 Data File Name Z:/ Data/ NMR/ 2016-07/ yht-3-92-H1/ 1/ fid
2 Title yht-3-92-H1
3 Comment PROTON
4 Origin Bruker BioSpin GmbH
5 Owner common
6 Site
7 Spectrometer spect
8 Author
9 Solvent CDCl3
10 Temperature 39.7
11 Pulse Sequence zg30
12 Experiment 1D
13 Number of Scans 8
14 Receiver Gain 128
15 Relaxation Delay 1.0000
16 Pulse Width 13.5000
17 Acquisition Time 1.9923
18 Acquisition Date 2016-07-22T22:30:00
19 Modification Date 2016-07-23T06:30:00
20 Spectrometer Frequency 400.23
21 Spectral Width 8223.7
22 Lowest Frequency -1640.3
23 Nucleus 1H
24 Acquired Size 16384
25 Spectral Size 32768

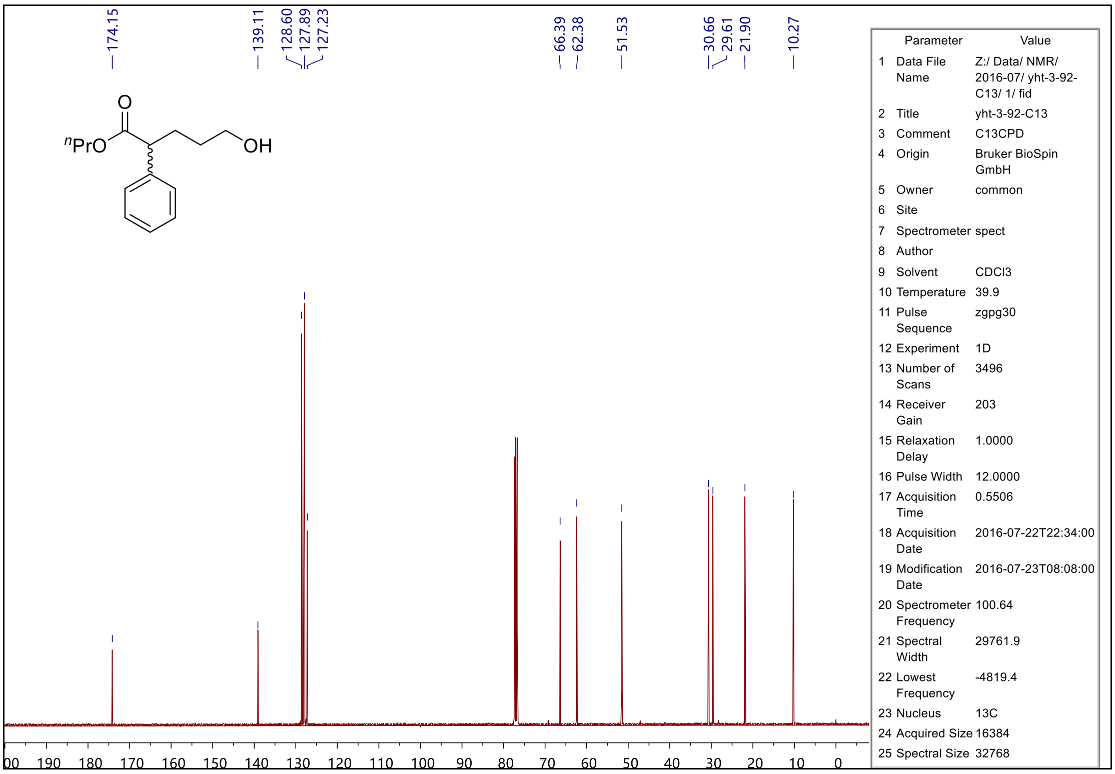

174.15 139.11 128.60 127.89 127.23 66.39 62.38 51.53 30.66 29.61 21.90 10.27
O
^{n}PrO
OH
00 190 180 170 160 150 140 130 120 110 100 90 80 70 60 50 40 30 20 10 0
Parameter Value
1 Data File Name Z:/ Data/ NMR/ 2016-07/ yht-3-92-C13/ 1/ fid
2 Title yht-3-92-C13
3 Comment C13CPD
4 Origin Bruker BioSpin GmbH
5 Owner common
6 Site
7 Spectrometer spect
8 Author
9 Solvent CDCl3
10 Temperature 39.9
11 Pulse Sequence zgpg30
12 Experiment 1D
13 Number of Scans 3496
14 Receiver Gain 203
15 Relaxation Delay 1.0000
16 Pulse Width 12.0000
17 Acquisition Time 0.5506
18 Acquisition Date 2016-07-22T22:34:00
19 Modification Date 2016-07-23T08:08:00
20 Spectrometer Frequency 100.64
21 Spectral Width 29761.9
22 Lowest Frequency -4819.4
23 Nucleus 13C
24 Acquired Size 16384
25 Spectral Size 32768

Compound 5

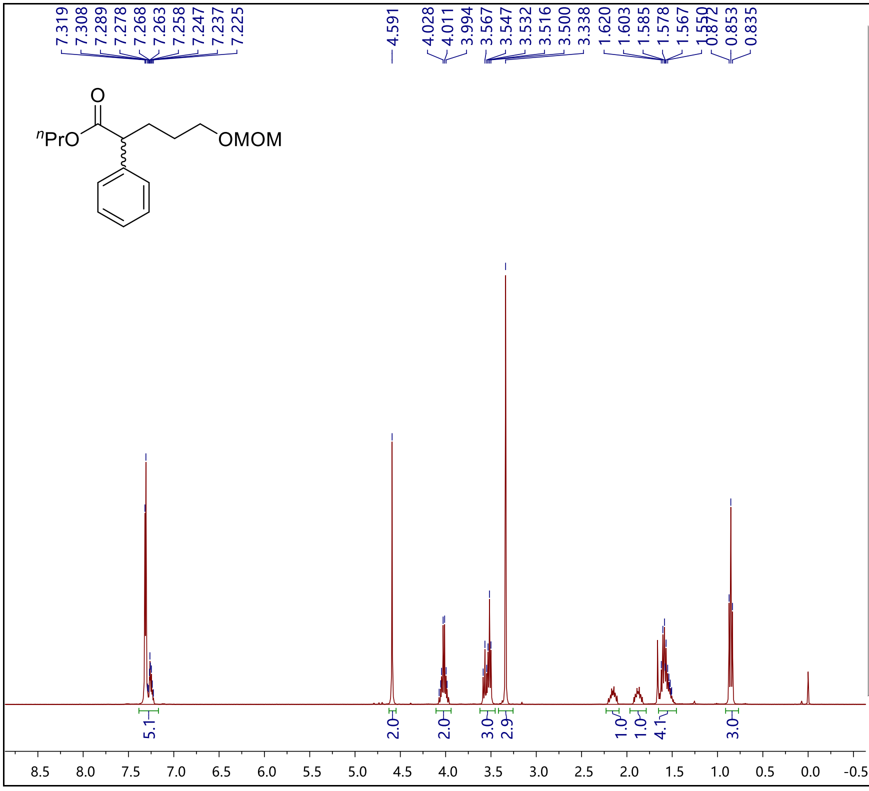

	Parameter	Value
1	Data File Name	Z:/ Data/ NMR/ 2016-07/ yht-3-97/ 1/ fid
2	Title	yht-3-97
3	Comment	PROTON
4	Origin	Bruker BioSpin GmbH
5	Owner	common
6	Site	
7	Spectrometer	spect
8	Author	
9	Solvent	CDCl3
10	Temperature	291.2
11	Pulse Sequence	zg30
12	Experiment	1D
13	Number of Scans	8
14	Receiver Gain	144
15	Relaxation Delay	1.0000
16	Pulse Width	13.5000
17	Acquisition Time	2.9999
18	Acquisition Date	2016-07-24T15:49:00
19	Modification Date	2016-07-24T23:49:00
20	Spectrometer Frequency	400.13
21	Spectral Width	8223.7
22	Lowest Frequency	-1649.9
23	Nucleus	1H
24	Acquired Size	24670
25	Spectral Size	65536

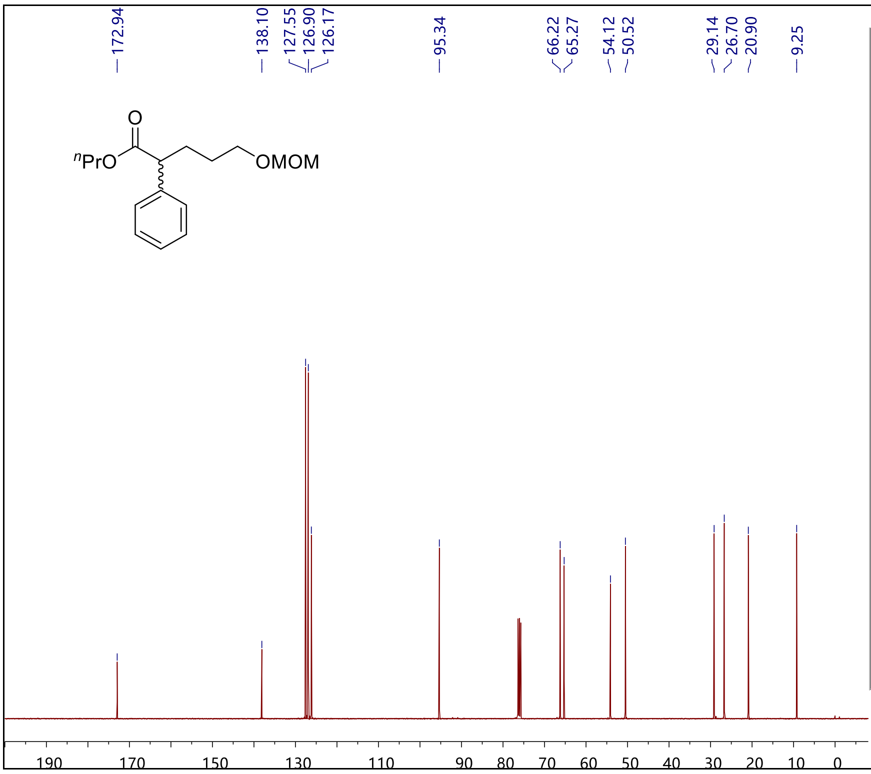

	Parameter	Value
1	Data File Name	Z:/ Data/ NMR/ 2016-07/ yht-3-97-C13/ 1/ fid
2	Title	yht-3-97-C13
3	Comment	C13CPD
4	Origin	Bruker BioSpin GmbH
5	Owner	common
6	Site	
7	Spectrometer	spect
8	Author	
9	Solvent	CDCl3
10	Temperature	291.2
11	Pulse Sequence	zgpg30
12	Experiment	1D
13	Number of Scans	10240
14	Receiver Gain	203
15	Relaxation Delay	1.0000
16	Pulse Width	12.5000
17	Acquisition Time	0.5506
18	Acquisition Date	2016-07-22T23:01:00
19	Modification Date	2016-07-23T11:37:00
20	Spectrometer Frequency	100.61
21	Spectral Width	29761.9
22	Lowest Frequency	-4924.0
23	Nucleus	13C
24	Acquired Size	16384
25	Spectral Size	32768

(−)-Preclamol

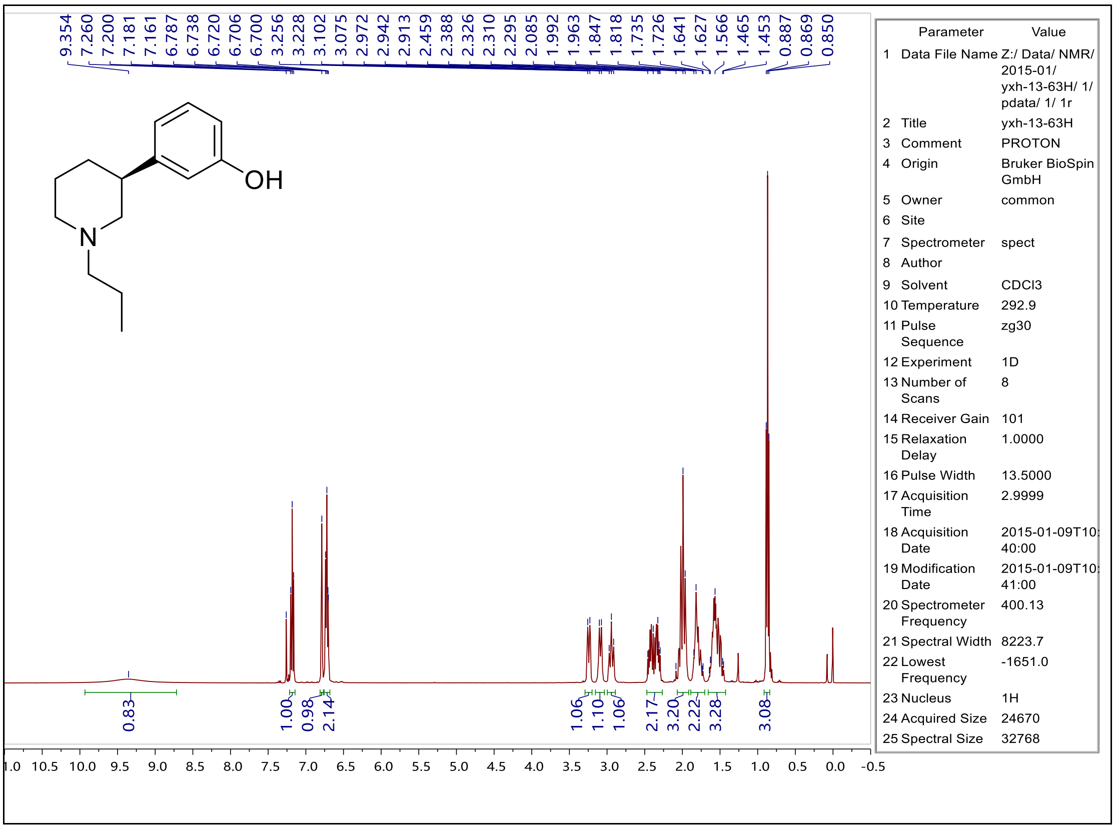
9.354 7.260 7.200 7.181 7.161 6.787 6.738 6.720 6.706 6.700 3.256 3.228 3.102 3.075 2.972 2.942 2.913 2.459 2.388 2.326 2.310 2.295 2.085 1.992 1.963 1.847 1.818 1.735 1.726 1.641 1.627 1.566 1.465 1.453 0.887 0.869 0.850
OH
N
0.83 1.00 0.98 2.14 1.06 1.10 1.06 2.17 3.20 2.22 3.28 3.08
1.0 10.5 10.0 9.5 9.0 8.5 8.0 7.5 7.0 6.5 6.0 5.5 5.0 4.5 4.0 3.5 3.0 2.5 2.0 1.5 1.0 0.5 0.0 -0.5
Parameter Value
1 Data File Name Z:/ Data/ NMR/ 2015-01/ yxh-13-63H/ 1/ pdata/ 1/ 1r
2 Title yxh-13-63H
3 Comment PROTON
4 Origin Bruker BioSpin GmbH
5 Owner common
6 Site
7 Spectrometer spect
8 Author
9 Solvent CDCl3
10 Temperature 292.9
11 Pulse Sequence zg30
12 Experiment 1D
13 Number of Scans 8
14 Receiver Gain 101
15 Relaxation Delay 1.0000
16 Pulse Width 13.5000
17 Acquisition Time 2.9999
18 Acquisition Date 2015-01-09T10: 40:00
19 Modification Date 2015-01-09T10: 41:00
20 Spectrometer Frequency 400.13
21 Spectral Width 8223.7
22 Lowest Frequency -1651.0
23 Nucleus 1H
24 Acquired Size 24670
25 Spectral Size 32768

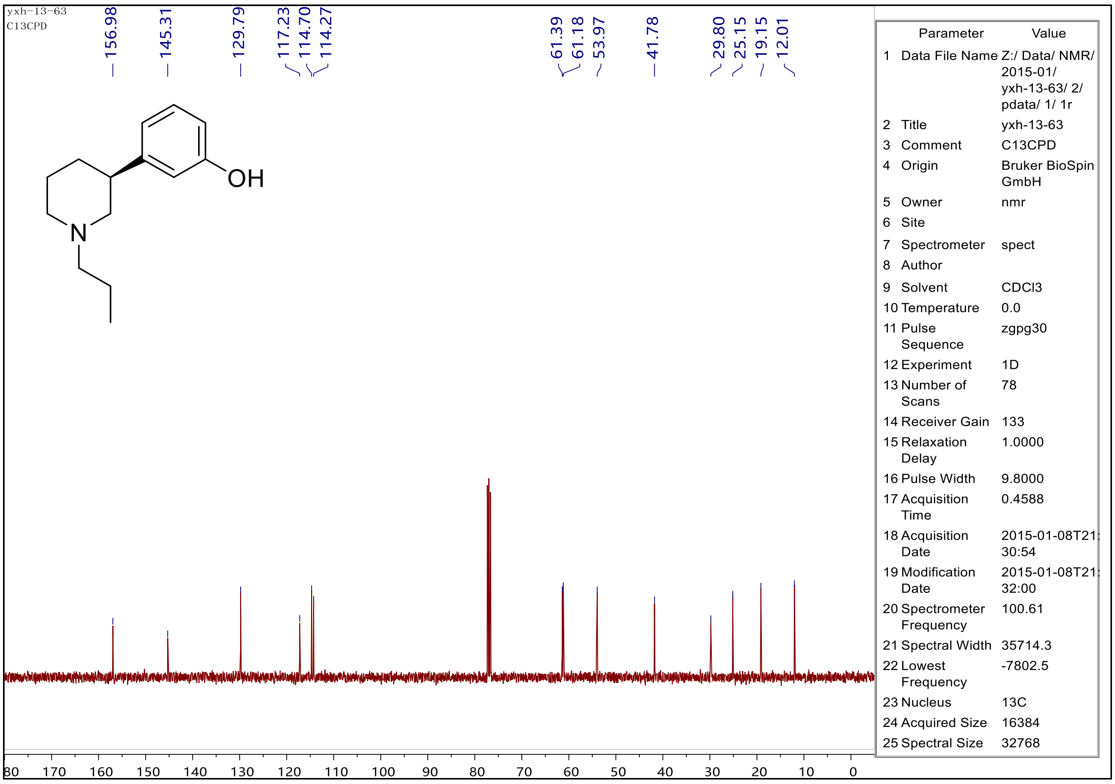
yxh-13-63
C13CPD
156.98 145.31 129.79 117.23 114.70 114.27 61.39 61.18 53.97 41.78 29.80 25.15 19.15 12.01
OH
N
80 170 160 150 140 130 120 110 100 90 80 70 60 50 40 30 20 10 0
Parameter Value
1 Data File Name Z:/ Data/ NMR/ 2015-01/ yxh-13-63/ 2/ pdata/ 1/ 1r
2 Title yxh-13-63
3 Comment C13CPD
4 Origin Bruker BioSpin GmbH
5 Owner nmr
6 Site
7 Spectrometer spect
8 Author
9 Solvent CDCl3
10 Temperature 0.0
11 Pulse Sequence zgpg30
12 Experiment 1D
13 Number of Scans 78
14 Receiver Gain 133
15 Relaxation Delay 1.0000
16 Pulse Width 9.8000
17 Acquisition Time 0.4588
18 Acquisition Date 2015-01-08T21: 30:54
19 Modification Date 2015-01-08T21: 32:00
20 Spectrometer Frequency 100.61
21 Spectral Width 35714.3
22 Lowest Frequency -7802.5
23 Nucleus 13C
24 Acquired Size 16384
25 Spectral Size 32768

Compound 7

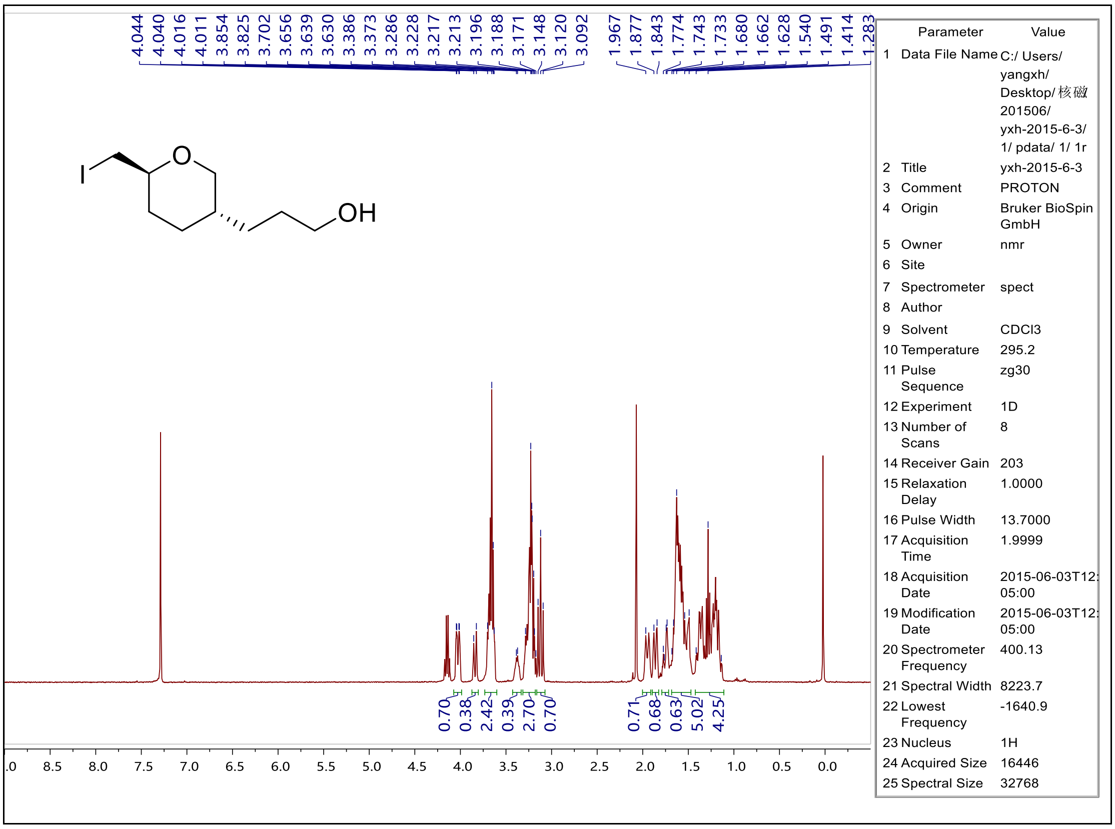

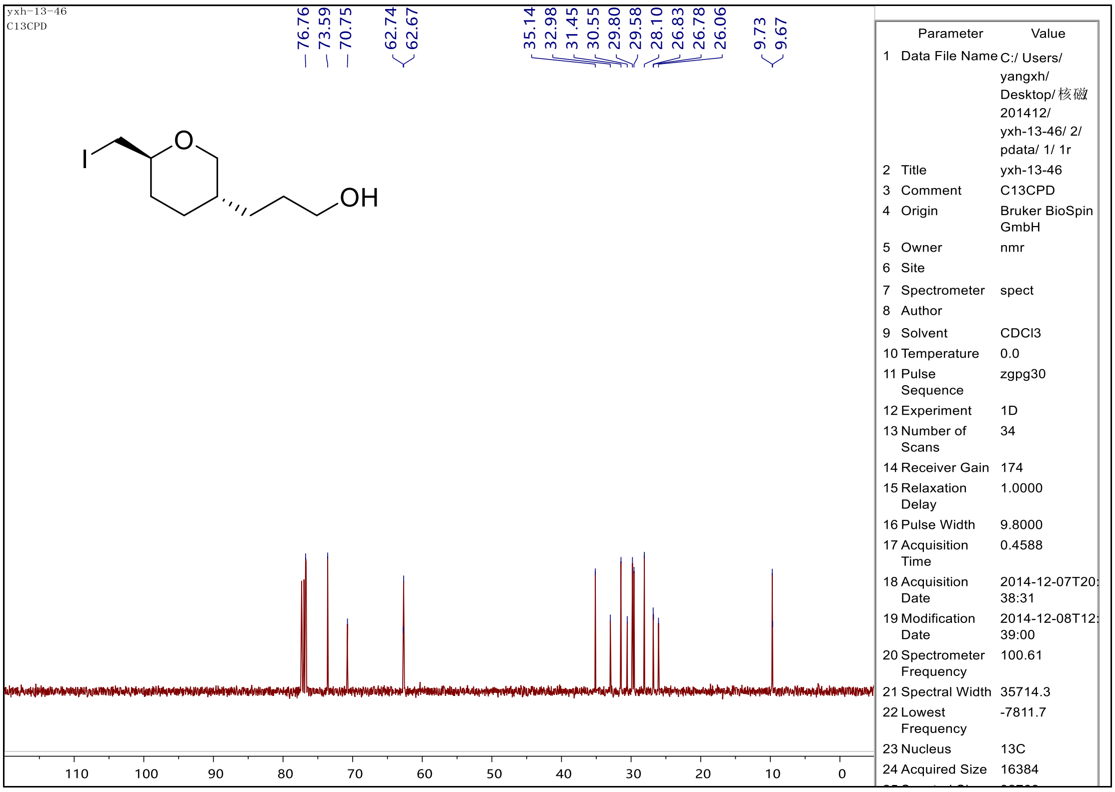

Compound 8

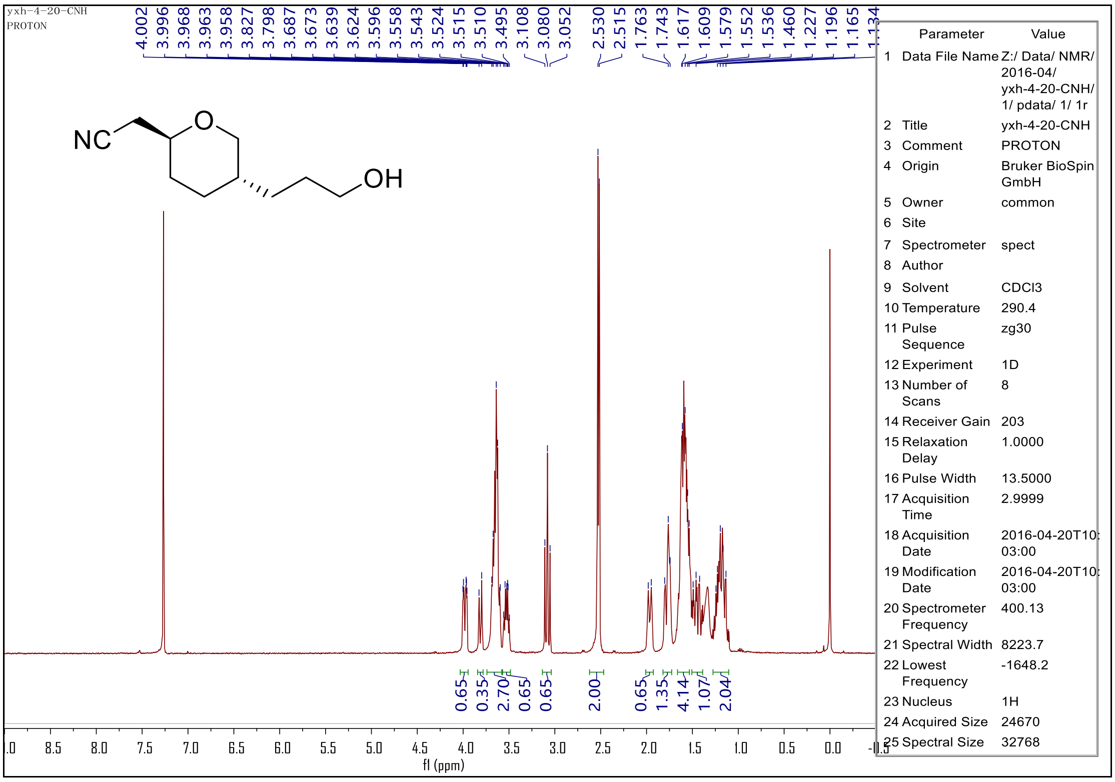

yxh-4-20-CNH
PROTON
4.002 3.996 3.968 3.963 3.958 3.827 3.798 3.687 3.673 3.639 3.624 3.596 3.558 3.543 3.524 3.515 3.510 3.495 3.108 3.080 3.052 2.530 2.515 1.763 1.743 1.617 1.609 1.579 1.552 1.536 1.460 1.227 1.196 1.165 1.134
NC
O
OH
0.65 0.35 2.70 0.65 0.65 2.00 0.65 1.35 4.14 1.07 2.04
fl (ppm)
Parameter Value
1 Data File Name Z:/ Data/ NMR/ 2016-04/ yxh-4-20-CNH/ 1/ pdata/ 1/ 1r
2 Title yxh-4-20-CNH
3 Comment PROTON
4 Origin Bruker BioSpin GmbH
5 Owner common
6 Site
7 Spectrometer spect
8 Author
9 Solvent CDCl3
10 Temperature 290.4
11 Pulse Sequence zg30
12 Experiment 1D
13 Number of Scans 8
14 Receiver Gain 203
15 Relaxation Delay 1.0000
16 Pulse Width 13.5000
17 Acquisition Time 2.9999
18 Acquisition Date 2016-04-20T10:03:00
19 Modification Date 2016-04-20T10:03:00
20 Spectrometer Frequency 400.13
21 Spectral Width 8223.7
22 Lowest Frequency -1648.2
23 Nucleus 1H
24 Acquired Size 24670
25 Spectral Size 32768

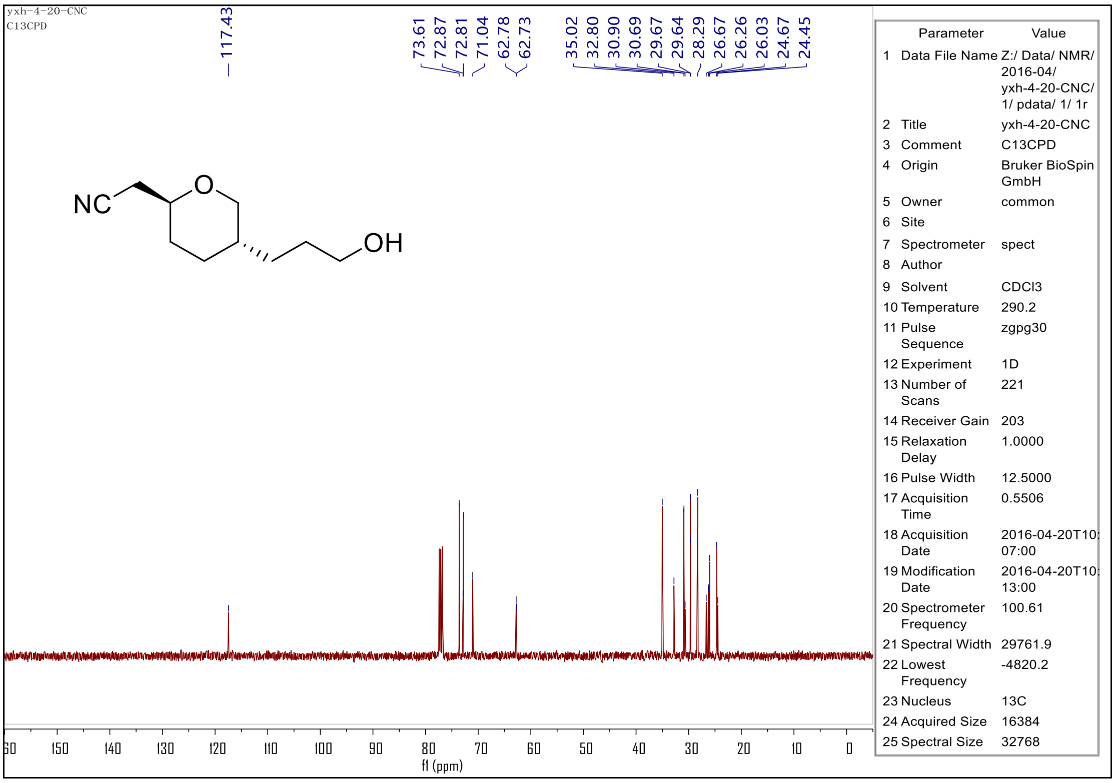

yxh-4-20-CNC
C13CPD
117.43
73.61 72.87 72.81 71.04 62.78 62.73
35.02 32.80 30.90 30.69 29.67 29.64 28.29 26.67 26.26 26.03 24.67 24.45
NC
O
OH
fl (ppm)
Parameter Value
1 Data File Name Z:/ Data/ NMR/ 2016-04/ yxh-4-20-CNC/ 1/ pdata/ 1/ 1r
2 Title yxh-4-20-CNC
3 Comment C13CPD
4 Origin Bruker BioSpin GmbH
5 Owner common
6 Site
7 Spectrometer spect
8 Author
9 Solvent CDCl3
10 Temperature 290.2
11 Pulse Sequence zgpg30
12 Experiment 1D
13 Number of Scans 221
14 Receiver Gain 203
15 Relaxation Delay 1.0000
16 Pulse Width 12.5000
17 Acquisition Time 0.5506
18 Acquisition Date 2016-04-20T10:07:00
19 Modification Date 2016-04-20T10:13:00
20 Spectrometer Frequency 100.61
21 Spectral Width 29761.9
22 Lowest Frequency -4820.2
23 Nucleus 13C
24 Acquired Size 16384
25 Spectral Size 32768

Compound 9

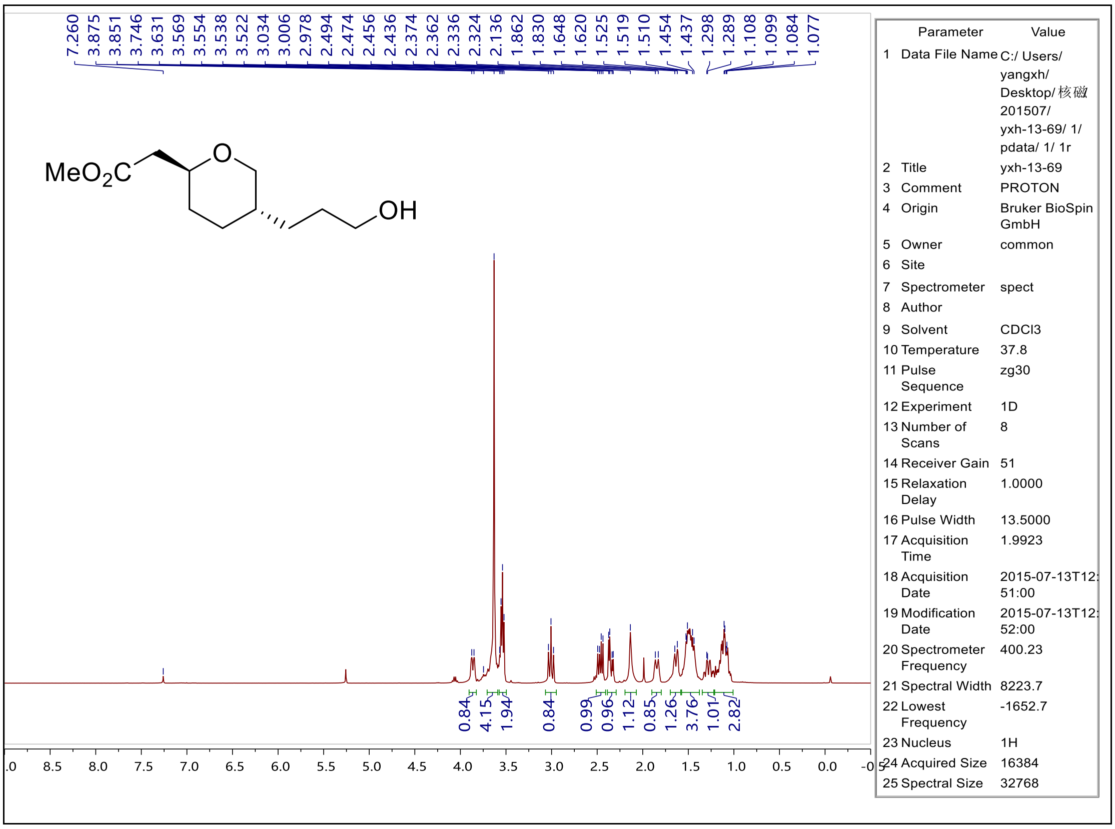

7.260
3.875
3.851
3.746
3.631
3.569
3.554
3.538
3.522
3.034
3.006
2.978
2.494
2.474
2.456
2.436
2.374
2.362
2.336
2.324
2.136
1.862
1.830
1.648
1.620
1.525
1.519
1.510
1.454
1.437
1.298
1.289
1.108
1.099
1.084
1.077
MeO_2C
O
OH
0.84
4.15
1.94
0.84
0.99
0.96
1.12
0.85
1.26
3.76
1.01
2.82
Parameter Value
1 Data File Name C:/ Users/ yangxh/ Desktop/ 核磁 201507/ yxh-13-69/ 1/ pdata/ 1/ 1r
2 Title yxh-13-69
3 Comment PROTON
4 Origin Bruker BioSpin GmbH
5 Owner common
6 Site
7 Spectrometer spect
8 Author
9 Solvent CDCl3
10 Temperature 37.8
11 Pulse Sequence zg30
12 Experiment 1D
13 Number of Scans 8
14 Receiver Gain 51
15 Relaxation Delay 1.0000
16 Pulse Width 13.5000
17 Acquisition Time 1.9923
18 Acquisition Date 2015-07-13T12: 51:00
19 Modification Date 2015-07-13T12: 52:00
20 Spectrometer Frequency 400.23
21 Spectral Width 8223.7
22 Lowest Frequency -1652.7
23 Nucleus 1H
24 Acquired Size 16384
25 Spectral Size 32768

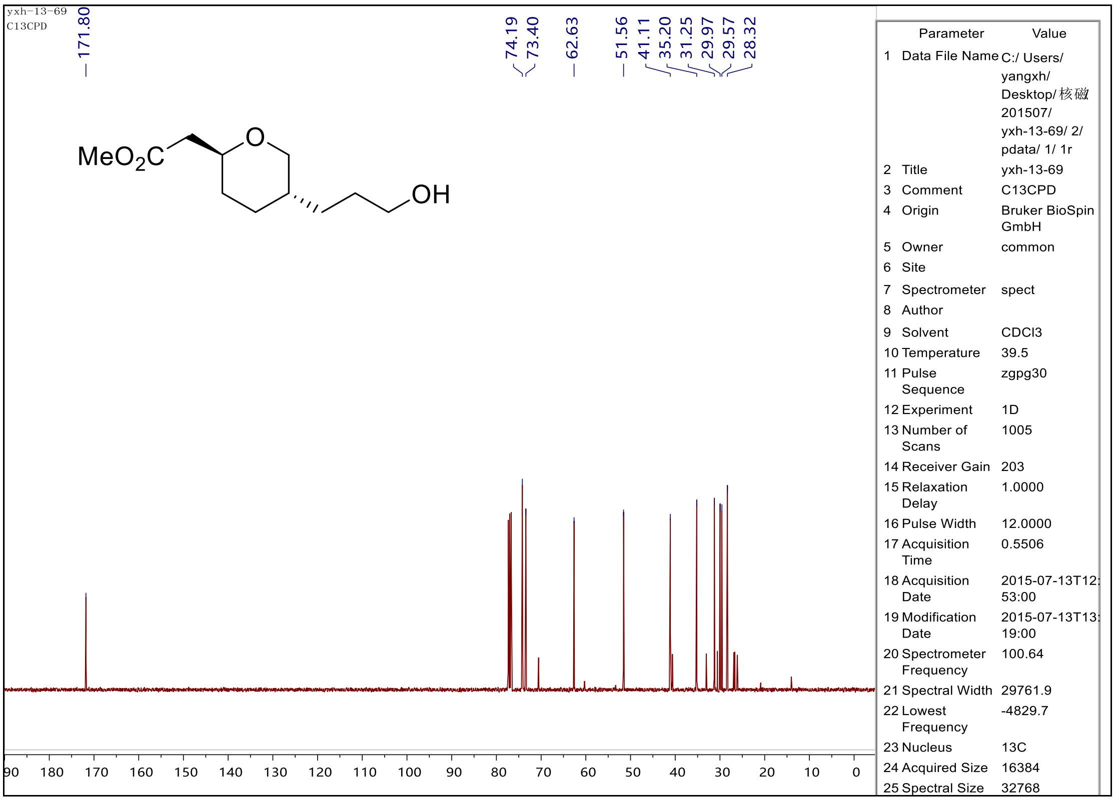

yxh-13-69
C13CPD
171.80
74.19
73.40
62.63
51.56
41.11
35.20
31.25
29.97
29.57
28.32
MeO_2C
O
OH
Parameter Value
1 Data File Name C:/ Users/ yangxh/ Desktop/ 核磁 201507/ yxh-13-69/ 2/ pdata/ 1/ 1r
2 Title yxh-13-69
3 Comment C13CPD
4 Origin Bruker BioSpin GmbH
5 Owner common
6 Site
7 Spectrometer spect
8 Author
9 Solvent CDCl3
10 Temperature 39.5
11 Pulse Sequence zgpg30
12 Experiment 1D
13 Number of Scans 1005
14 Receiver Gain 203
15 Relaxation Delay 1.0000
16 Pulse Width 12.0000
17 Acquisition Time 0.5506
18 Acquisition Date 2015-07-13T12: 53:00
19 Modification Date 2015-07-13T13: 19:00
20 Spectrometer Frequency 100.64
21 Spectral Width 29761.9
22 Lowest Frequency -4829.7
23 Nucleus 13C
24 Acquired Size 16384
25 Spectral Size 32768

(F) HPLC charts for hydrogenation products

(*R*)-2-Phenylpentane-1,5-diol (3a)

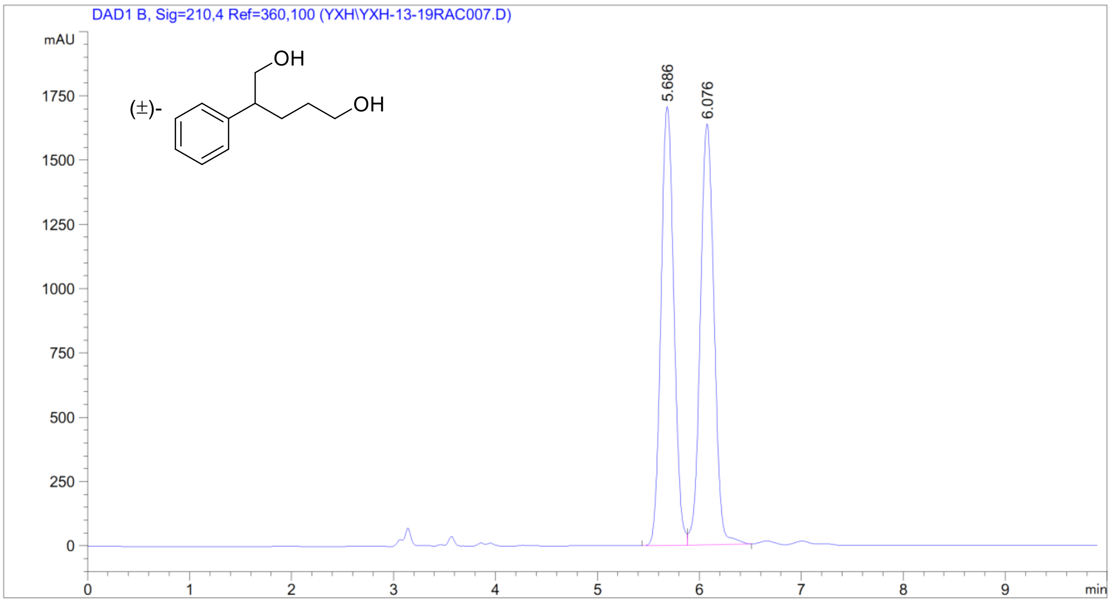

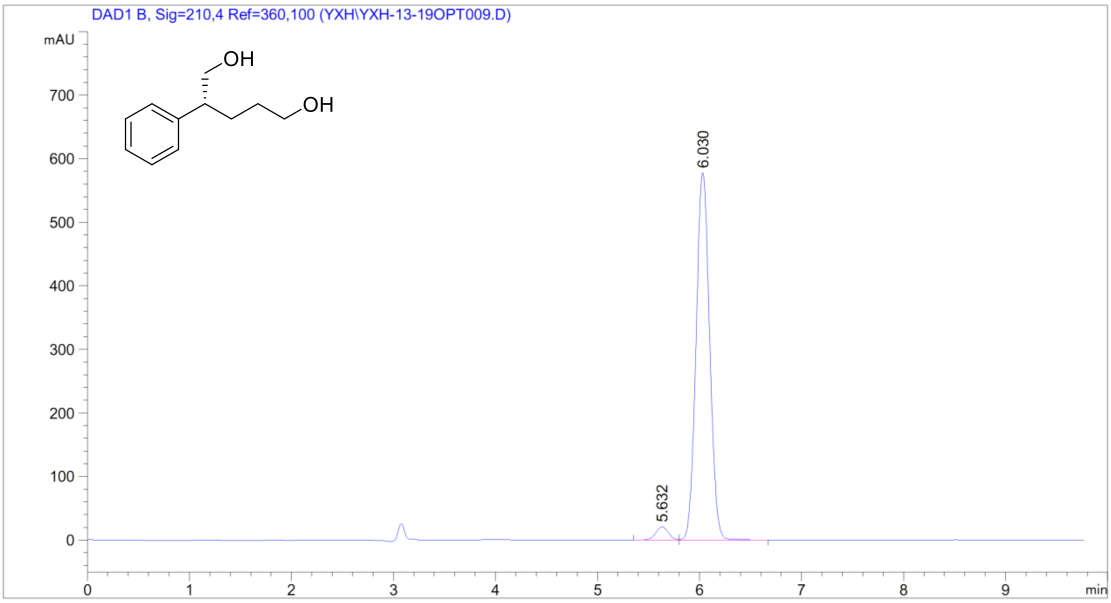

Peak #	RetTime [min]	Type	Width [min]	Area [mAU*s]	Height [mAU]	Area %
1	5.632	BV	0.1336	178.10455	20.86667	3.2320
2	6.030	VB	0.1435	5332.56934	578.53839	96.7680

(*R*)-2-(4-Chlorophenyl)pentane-1,5-diol (3b)

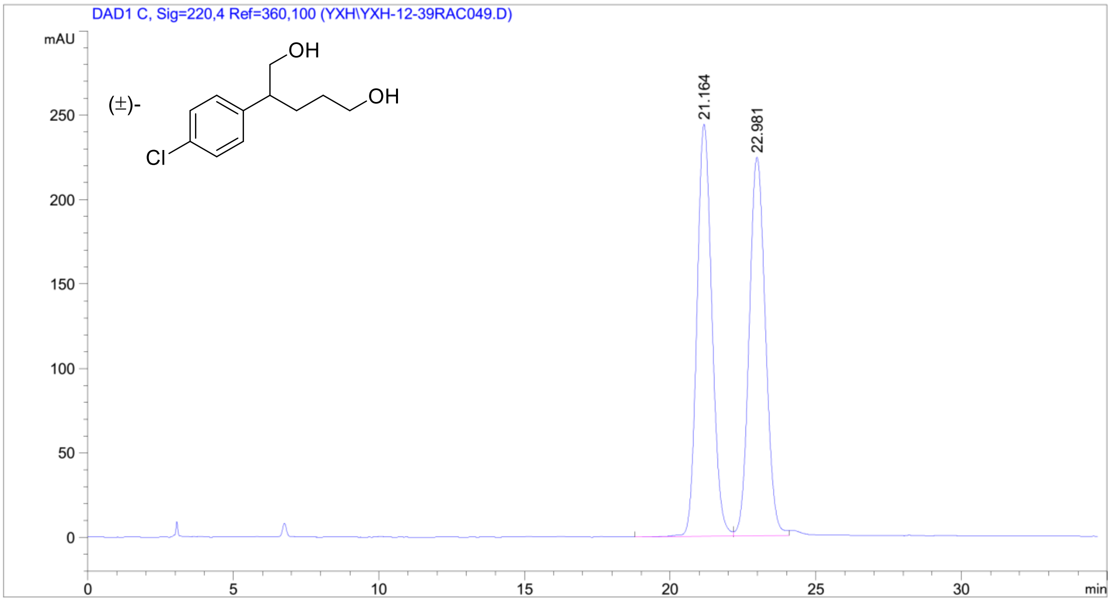

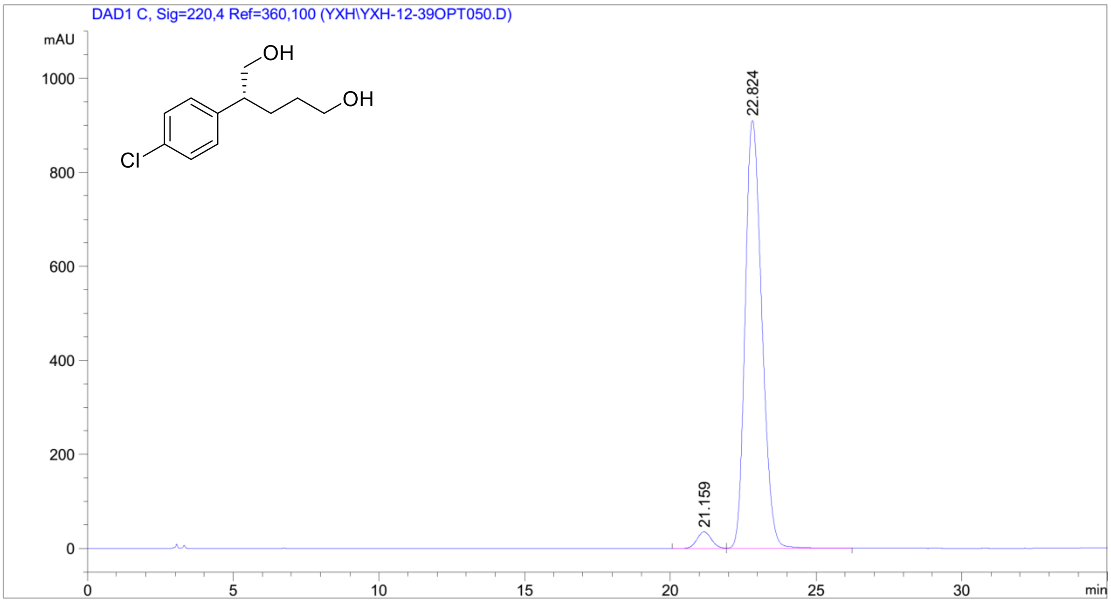

Peak #	RetTime [min]	Type	Width [min]	Area [mAU*s]	Height [mAU]	Area %
1	21.159	BV	0.5393	1223.11536	35.37984	3.2675
2	22.824	VB	0.6200	3.62100e4	910.54260	96.7325

(*R*)-2-(4-Tolyl)pentane-1,5-diol (3c)

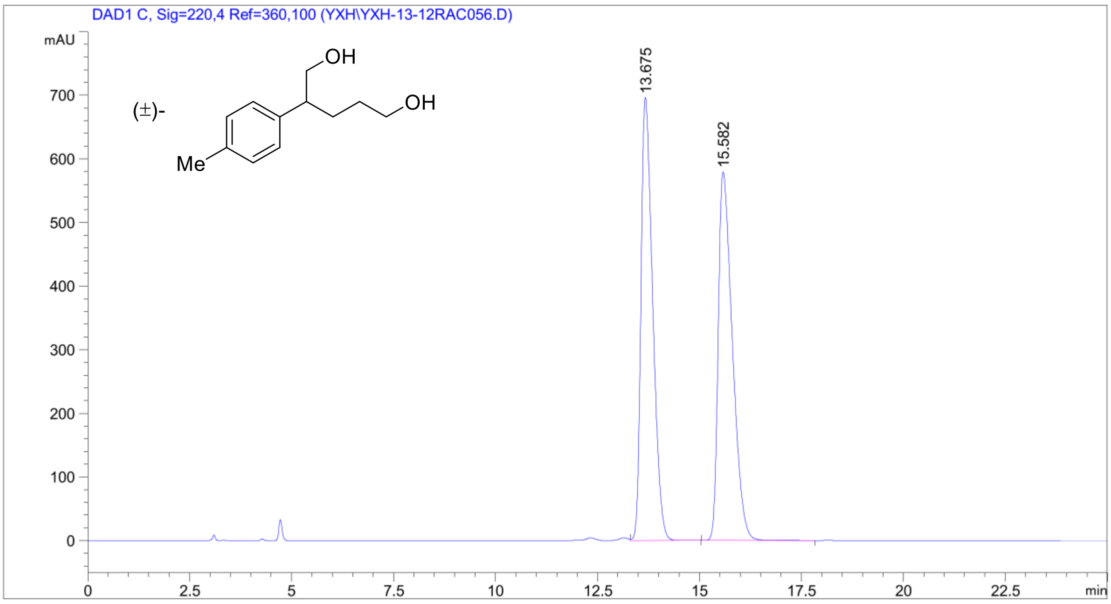

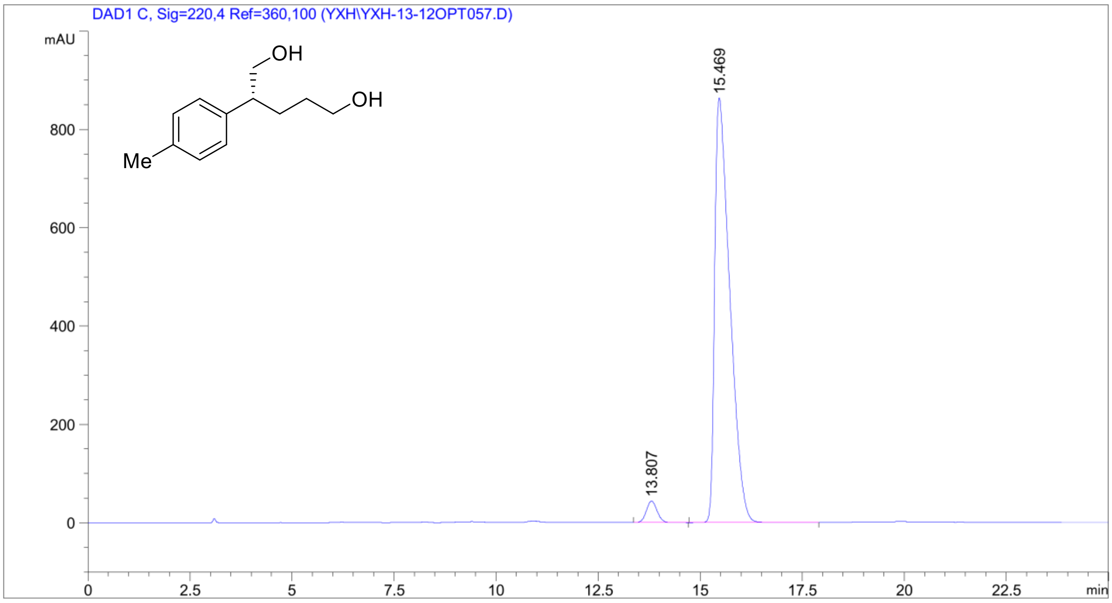

Peak #	RetTime [min]	Type	Width [min]	Area [mAU*s]	Height [mAU]	Area %
1	13.807	VB	0.2824	803.22278	43.70848	3.4841
2	15.469	BB	0.3929	2.22510e4	862.75928	96.5159

(*R*)-2-(4-Methoxyphenyl)pentane-1,5-diol (3d)

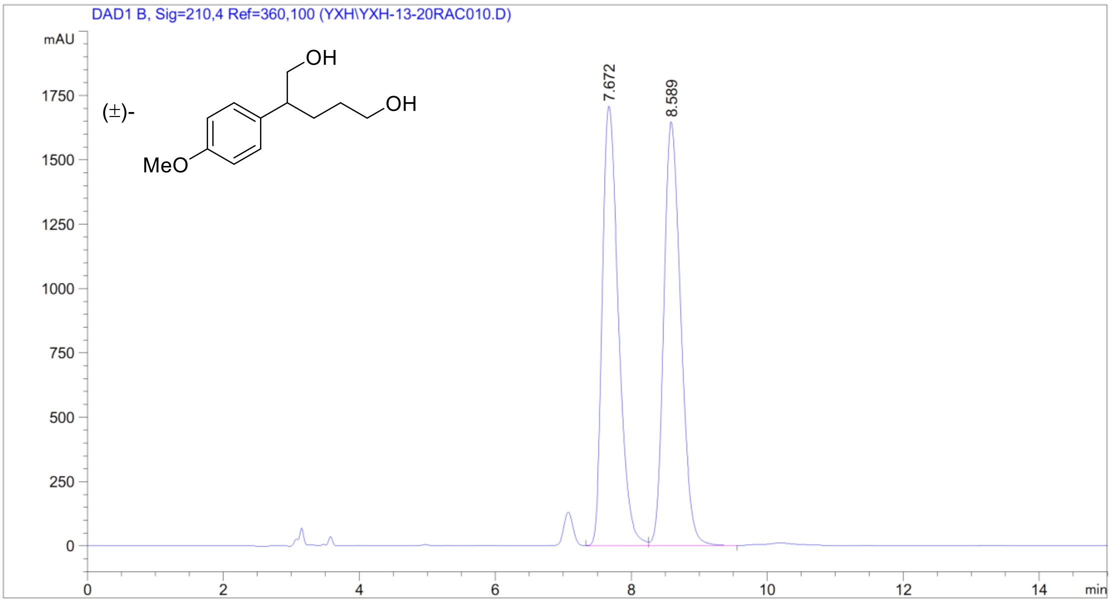

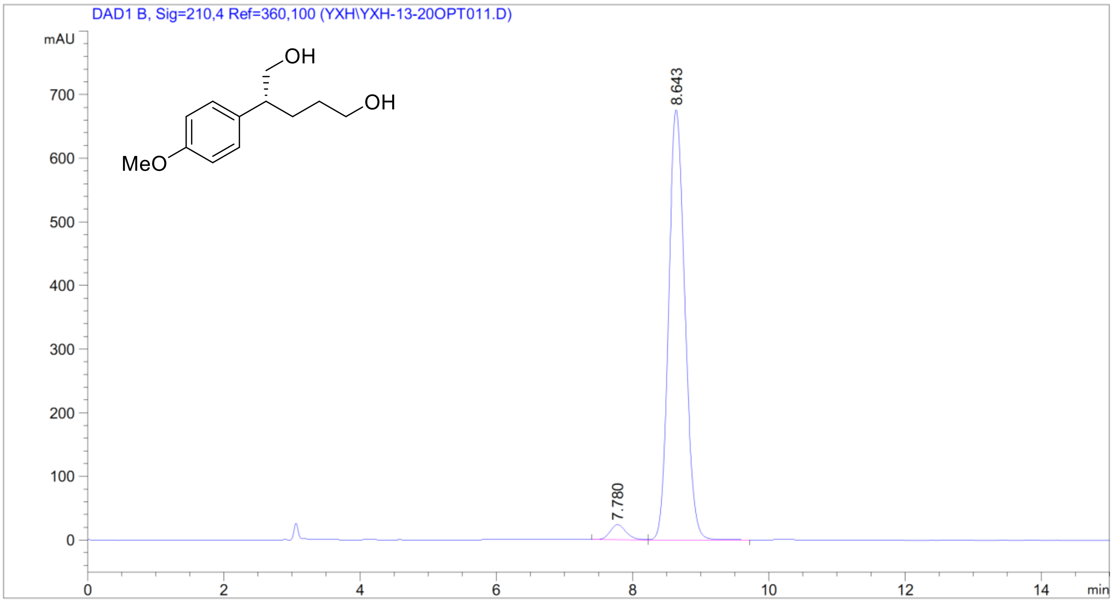

Peak #	RetTime [min]	Type	Width [min]	Area [mAU*s]	Height [mAU]	Area %
1	7.780	BV	0.2493	388.67471	23.95855	3.3540
2	8.643	VB	0.2573	1.11996e4	675.92743	96.6460

(*R*)-2-(3-Chlorophenyl)pentane-1,5-diol (3e)

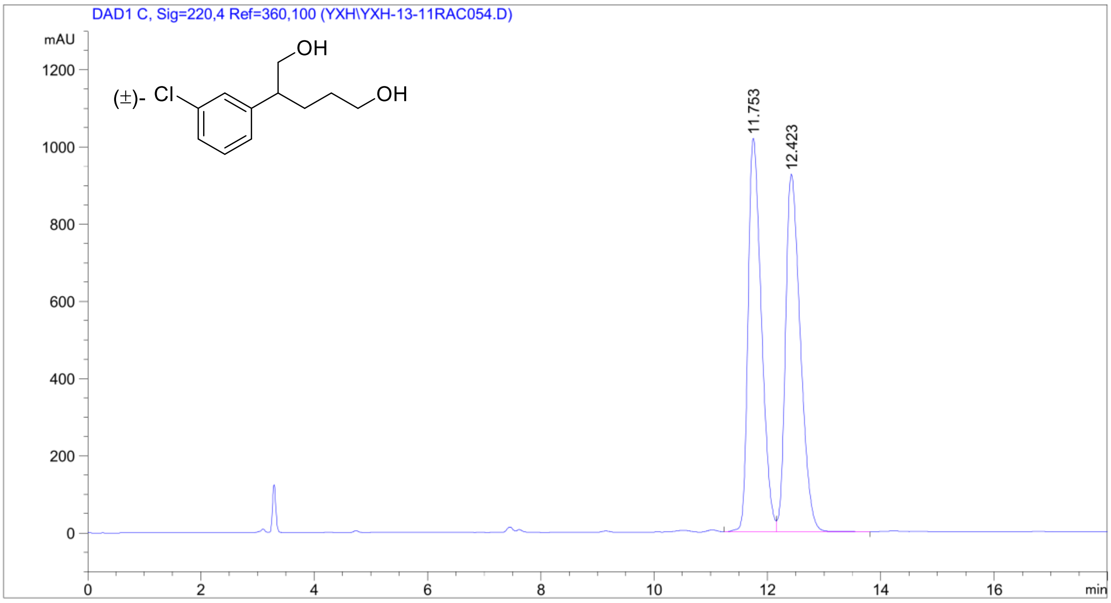

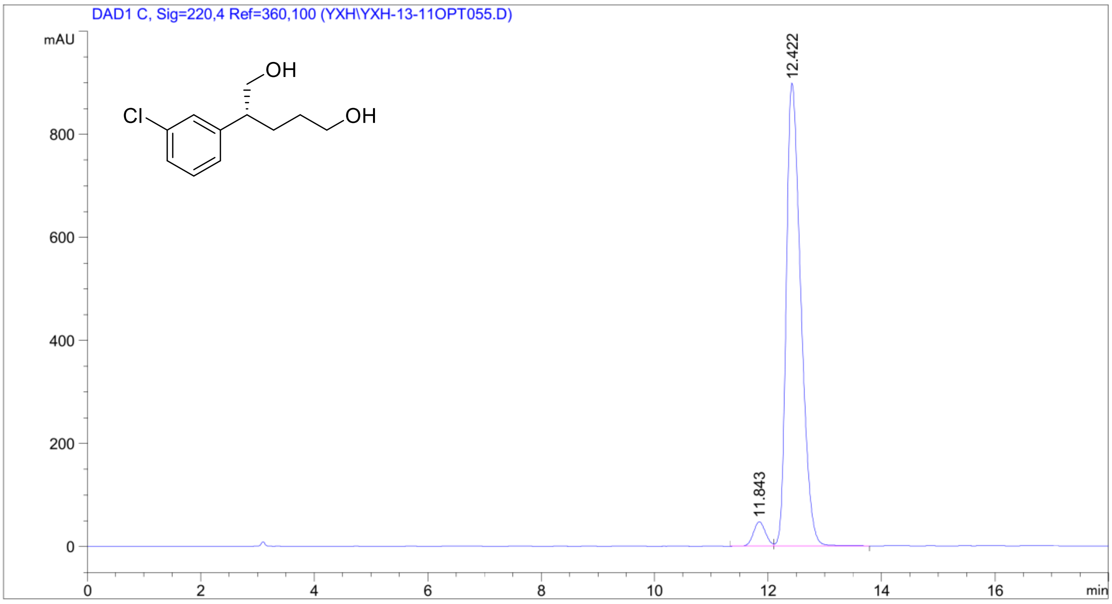

Peak #	RetTime [min]	Type	Width [min]	Area [mAU*s]	Height [mAU]	Area %
1	11.843	BV	0.2333	702.26642	47.27721	4.1978
2	12.422	VB	0.2763	1.60269e4	897.75177	95.8022

(*R*)-2-(3-Tolyl)pentane-1,5-diol (3f)

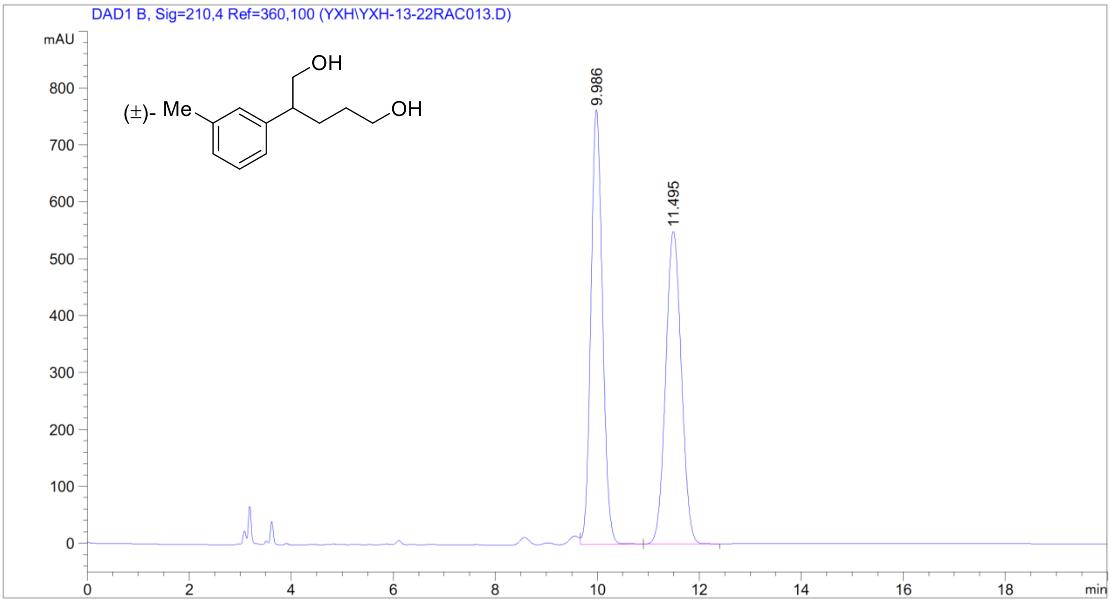

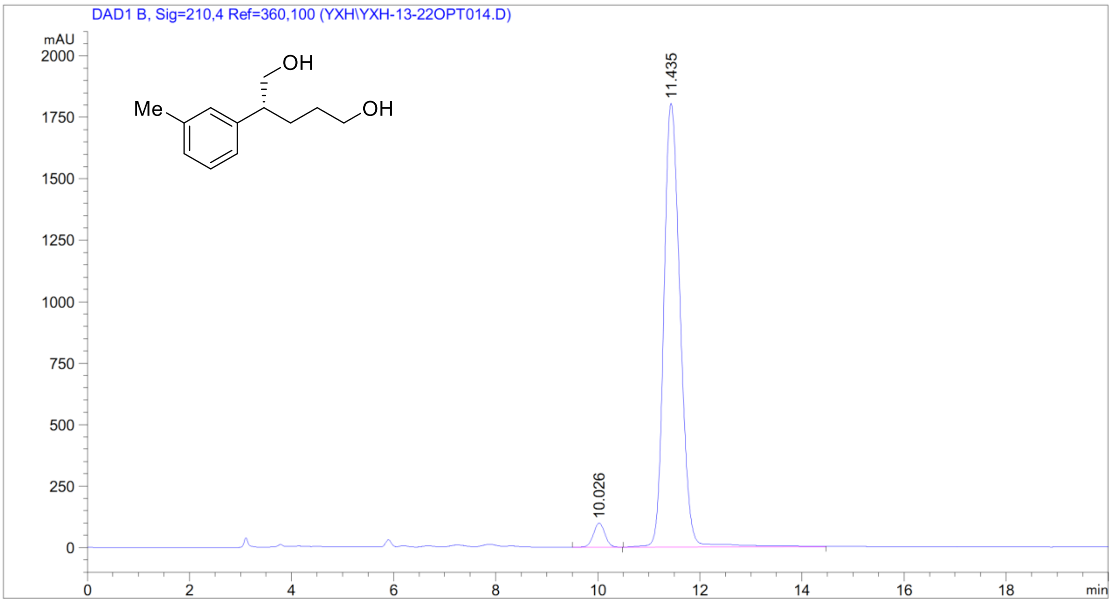

Peak #	RetTime [min]	Type	Width [min]	Area [mAU*s]	Height [mAU]	Area %
1	10.026	BB	0.2447	1527.18933	97.50394	3.6482
2	11.435	BV	0.3487	4.03343e4	1805.00513	96.3518

(*R*)-2-(3-Methoxyphenyl)pentane-1,5-diol (3g)

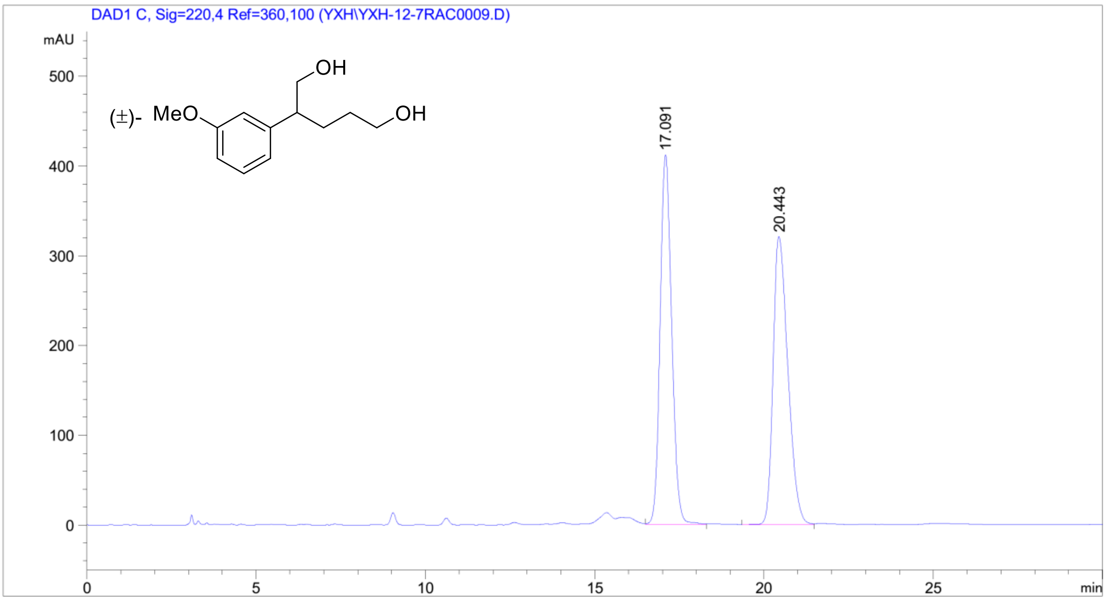

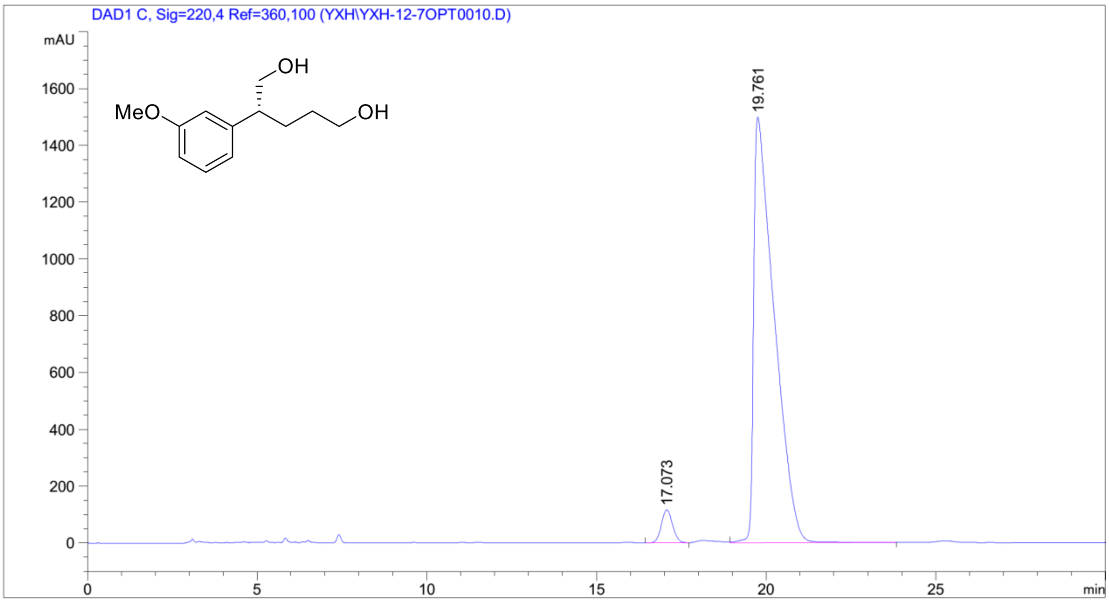

Peak #	RetTime [min]	Type	Width [min]	Area [mAU*s]	Height [mAU]	Area %
1	17.073	BB	0.3599	2656.24780	115.68431	4.0609
2	19.761	VV	0.6006	6.27546e4	1498.40247	95.9391

(*R*)-2-(3,4-Dichlorophenyl)pentane-1,5-diol (3h)

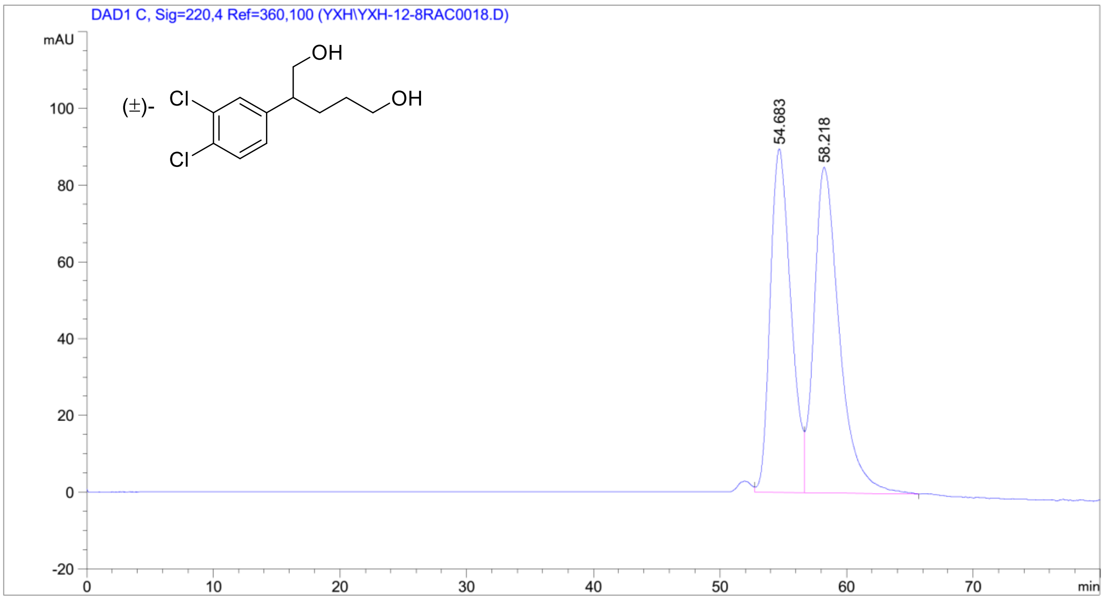

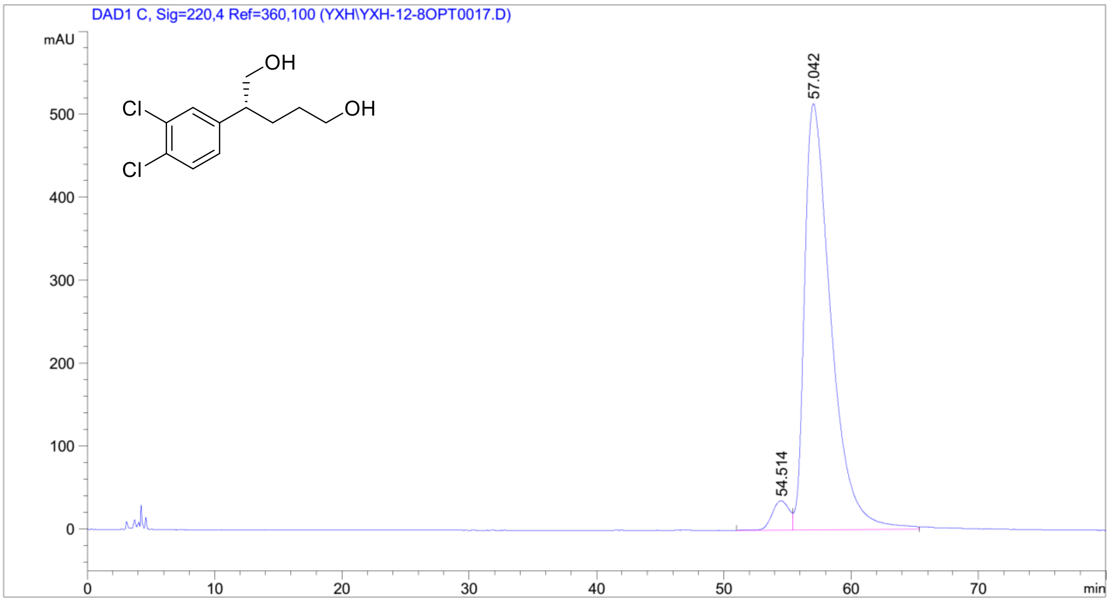

Peak #	RetTime [min]	Type	Width [min]	Area [mAU*s]	Height [mAU]	Area %
1	54.514	BV	1.4296	3281.66504	35.39734	4.2439
2	57.042	VV	2.1376	7.40454e4	513.72180	95.7561

(*R*)-2-(3,4-Dimethoxyphenyl)pentane-1,5-diol (3i)

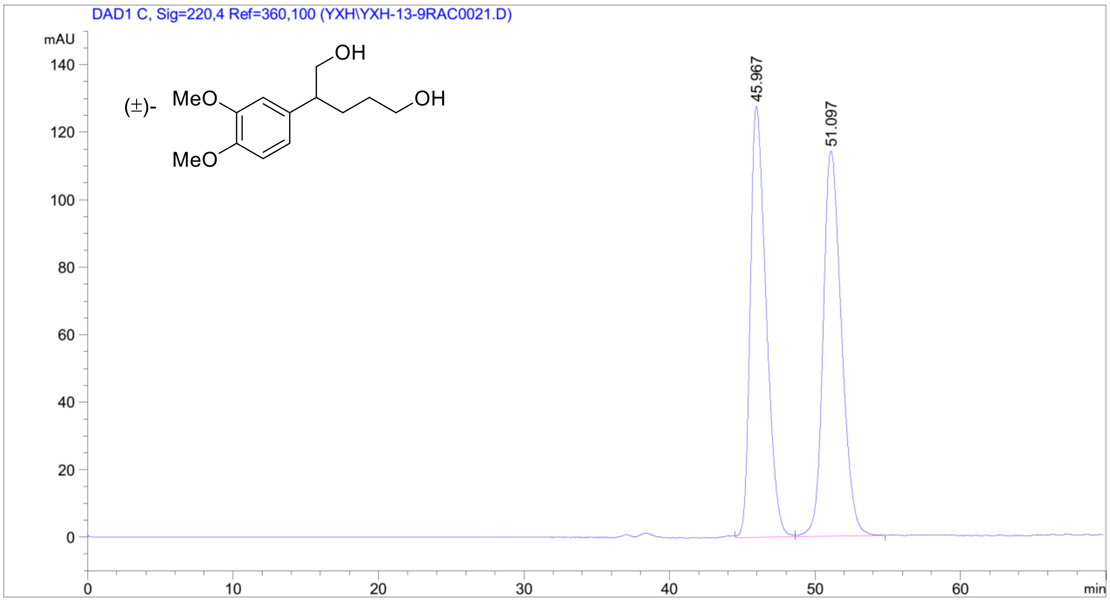

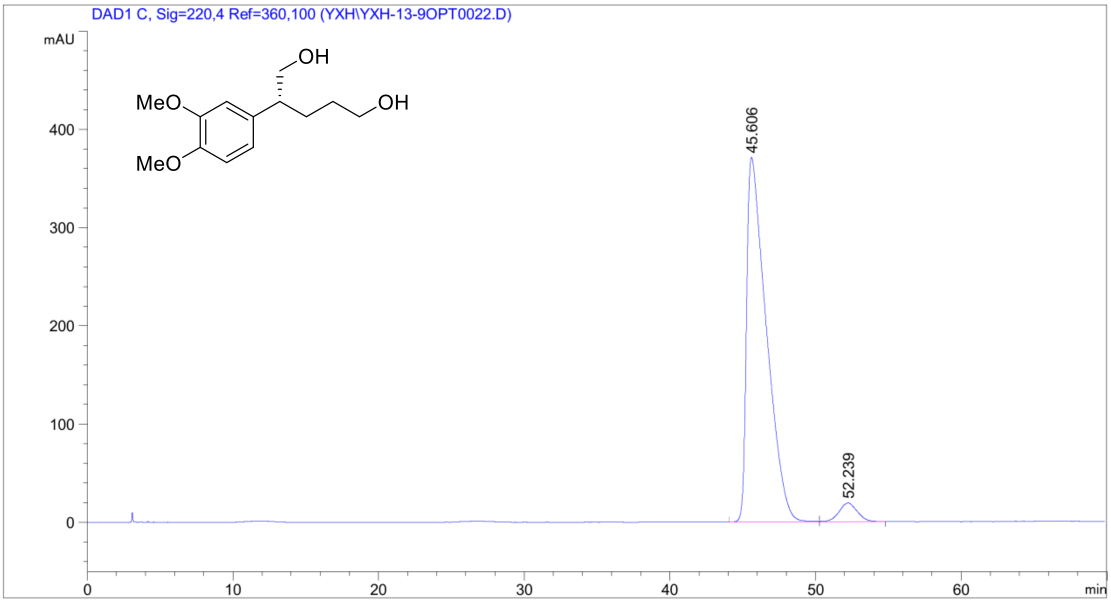

Peak #	RetTime [min]	Type	Width [min]	Area [mAU*s]	Height [mAU]	Area %
1	45.606	BV	1.3241	3.48732e4	371.19522	95.3972
2	52.239	VB	1.3059	1682.60229	19.05971	4.6028

(*R*)-2-(2-Chlorophenyl)pentane-1,5-diol (3j)

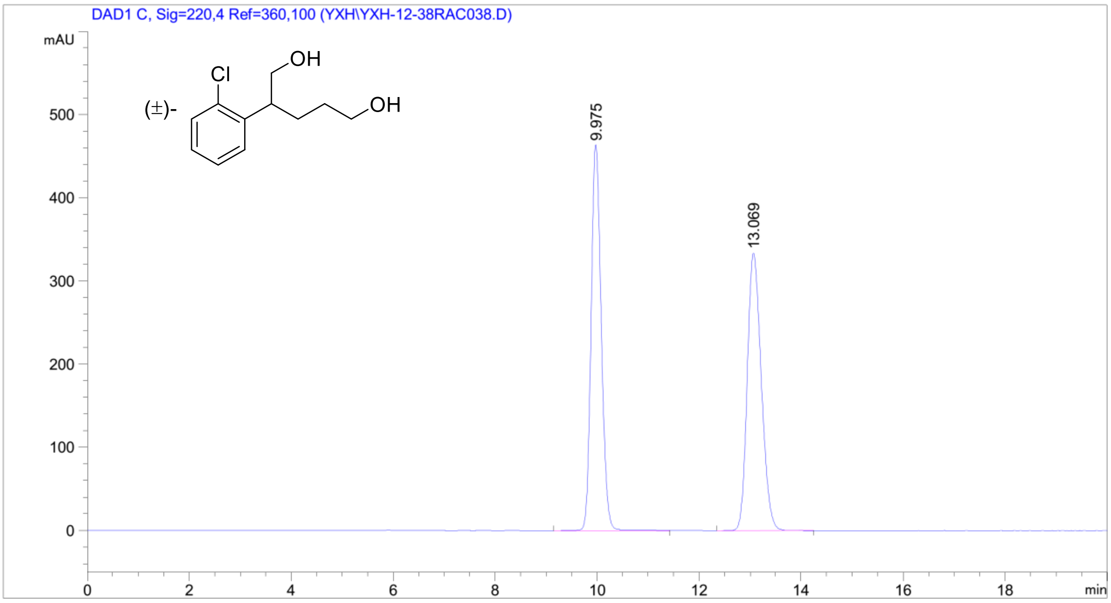

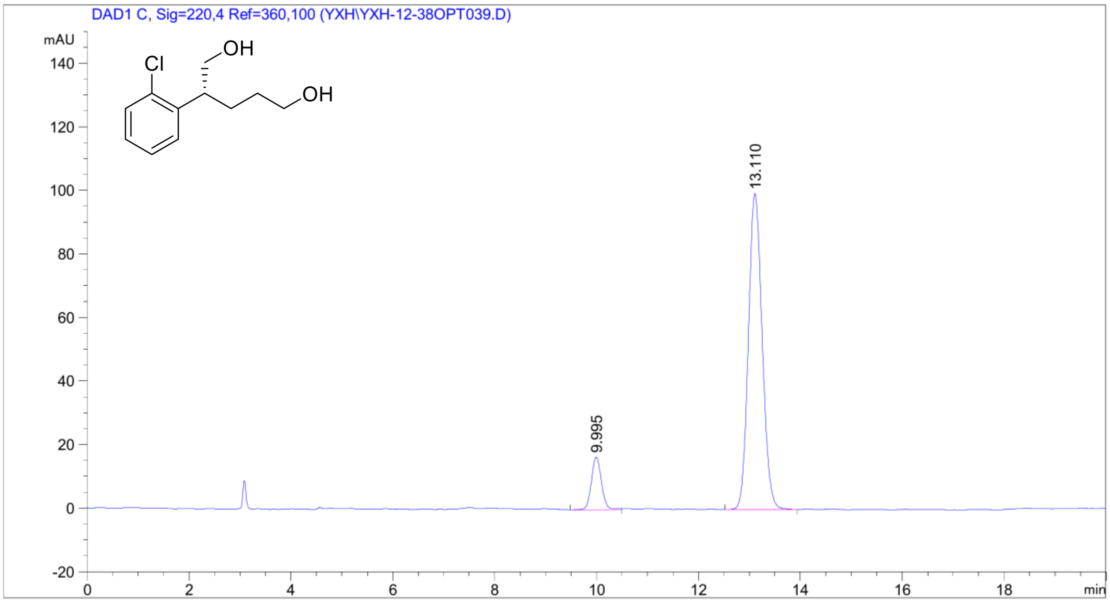

Peak #	RetTime [min]	Type	Width [min]	Area [mAU*s]	Height [mAU]	Area %
1	9.995	BV	0.2134	229.17755	16.56094	10.9514
2	13.110	BB	0.2948	1863.49719	99.38586	89.0486

(*R*)-2-(2-Tolyl)pentane-1,5-diol (3k)

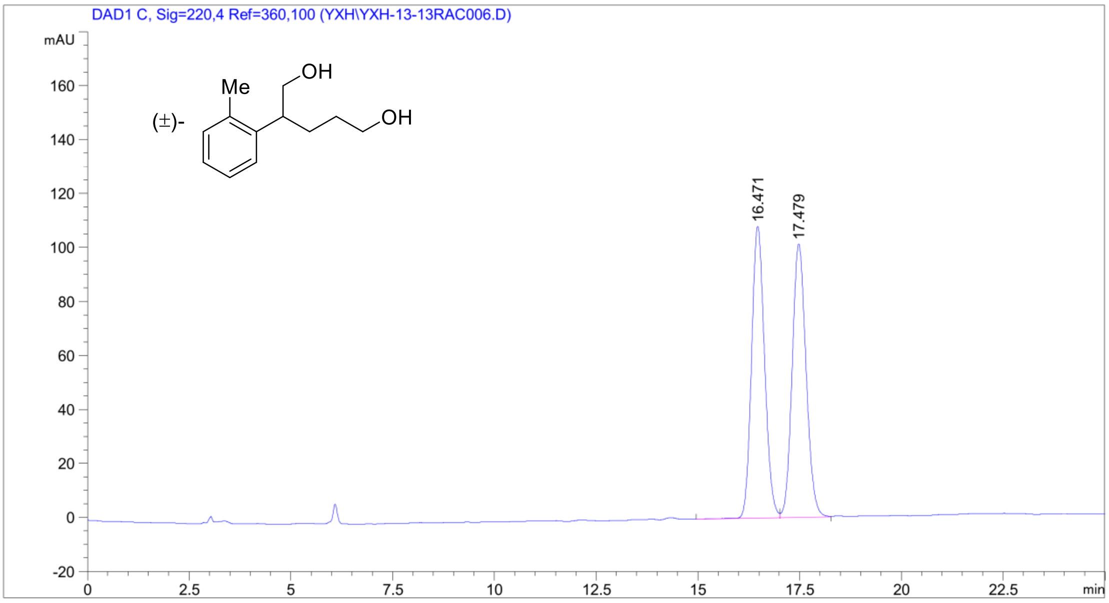

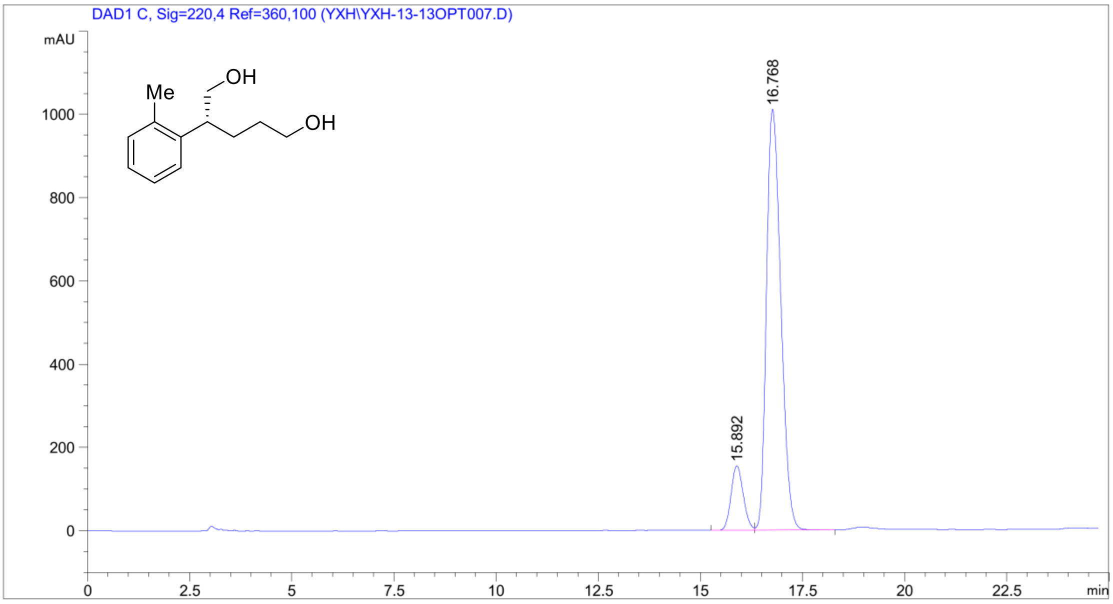

Peak #	RetTime [min]	Type	Width [min]	Area [mAU*s]	Height [mAU]	Area %
1	15.892	BV	0.3228	3226.01245	155.02222	11.5618
2	16.768	VV	0.3849	2.46763e4	1010.63812	88.4382

(*R*)-2-(2-Methoxyphenyl)pentane-1,5-diol (3l)

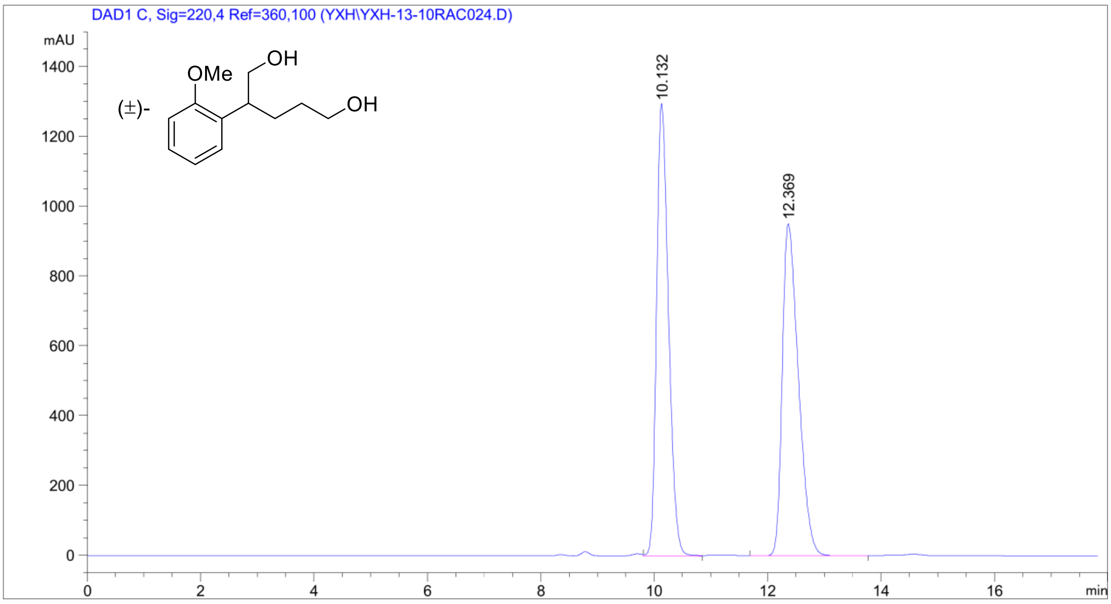

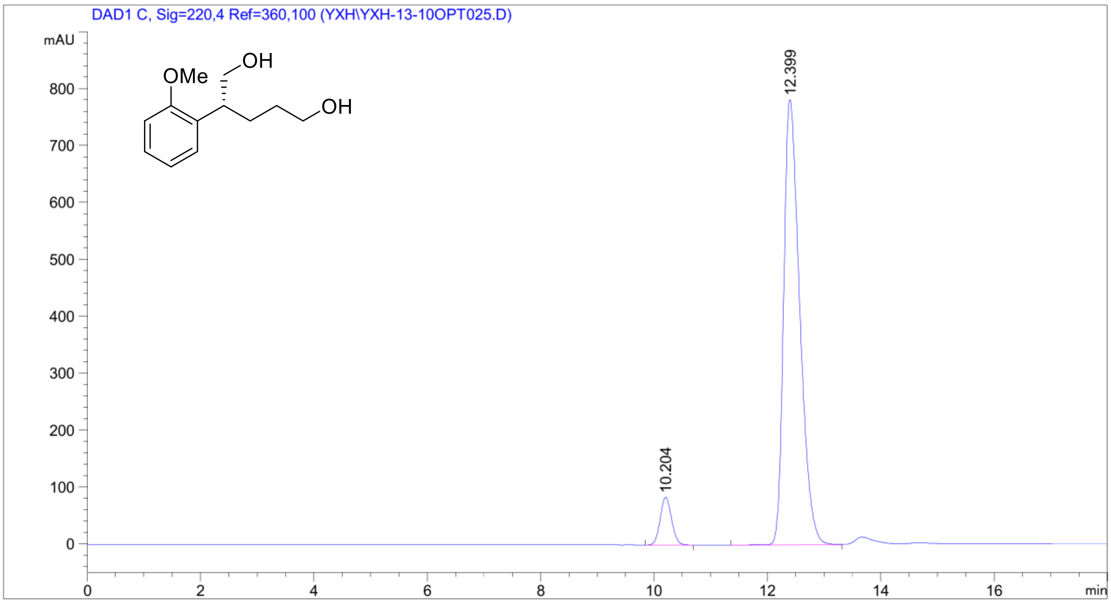

Peak #	RetTime [min]	Type	Width [min]	Area [mAU*s]	Height [mAU]	Area %
1	10.204	BB	0.2189	1170.08374	83.81371	7.0763
2	12.399	BV	0.3052	1.53652e4	782.08508	92.9237

(*S*)-2-Methylpentane-1,5-diol (3m)

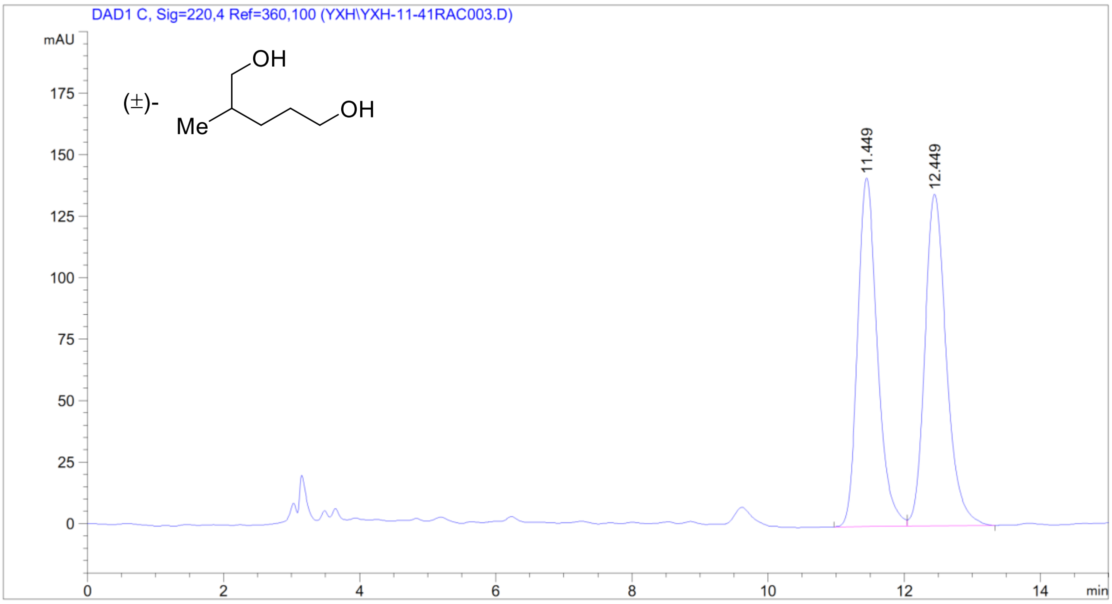

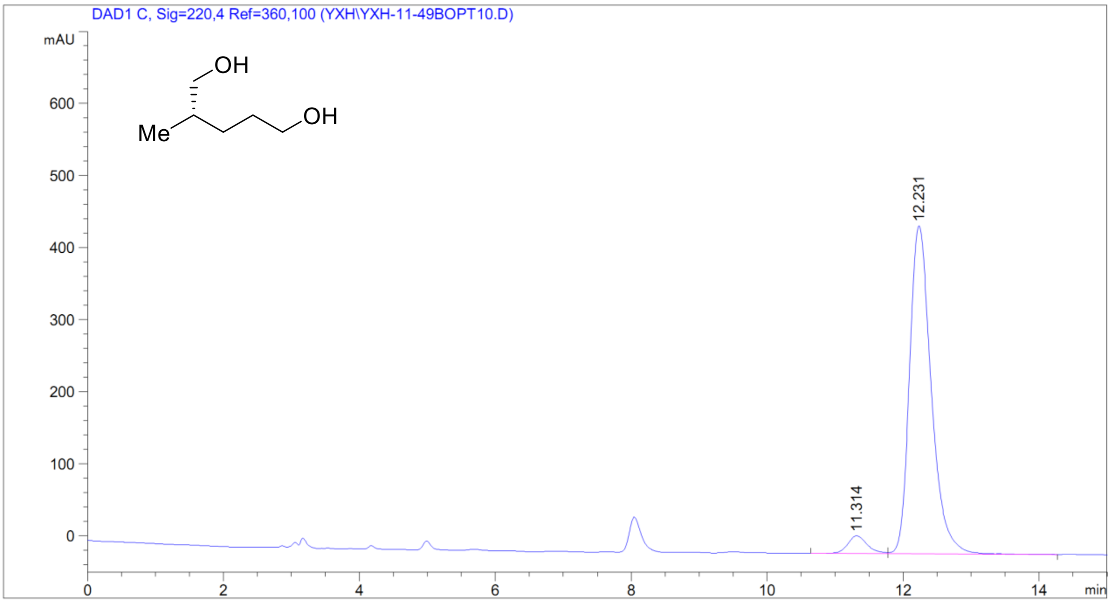

Peak #	RetTime [min]	Type	Width [min]	Area [mAU*s]	Height [mAU]	Area %
1	11.314	BV	0.3051	487.04681	24.38050	4.6194
2	12.231	VB	0.3399	1.00565e4	454.88486	95.3806

(*S*)-2-Ethylpentane-1,5-diol (3n)

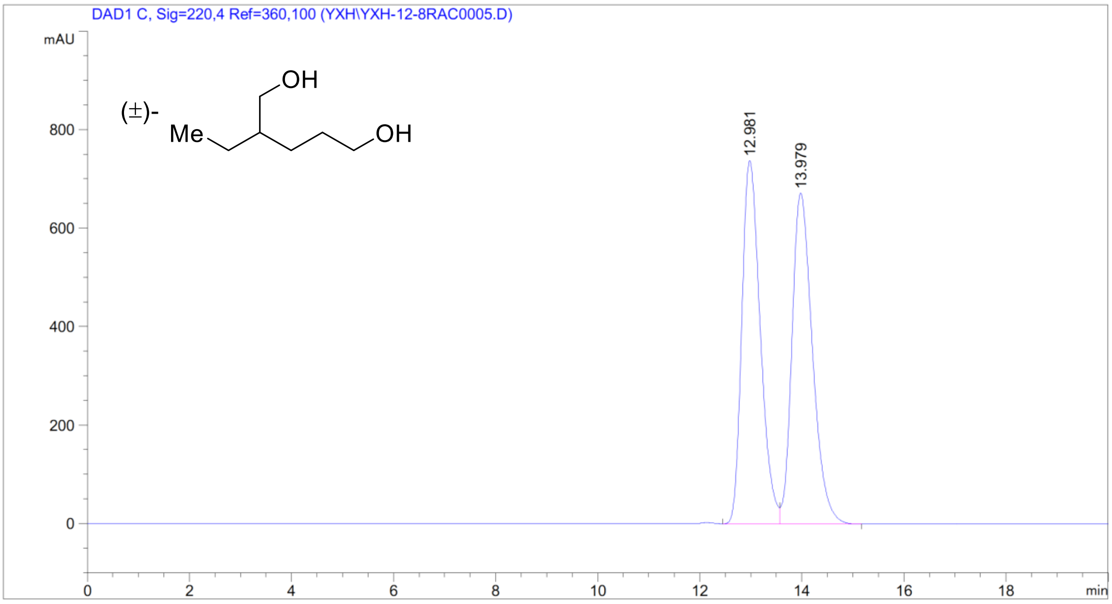

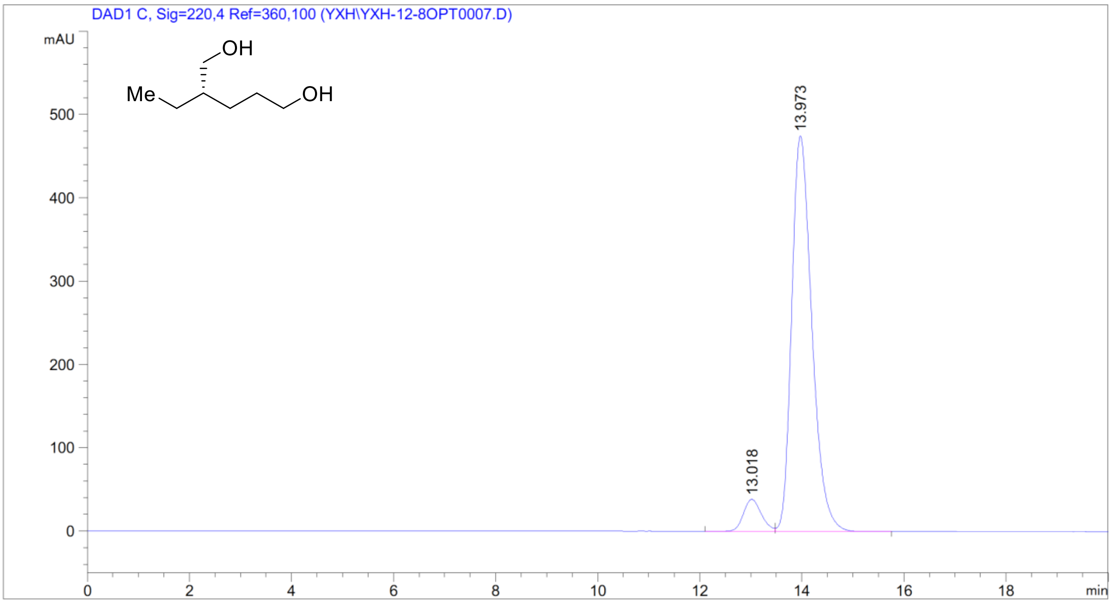

Peak #	RetTime [min]	Type	Width [min]	Area [mAU*s]	Height [mAU]	Area %
1	13.018	BV	0.3785	946.38873	38.54408	6.7492
2	13.973	VB	0.4252	1.30758e4	474.91827	93.2508

(*R*)-2-Isopropylpentane-1,5-diol (3o)

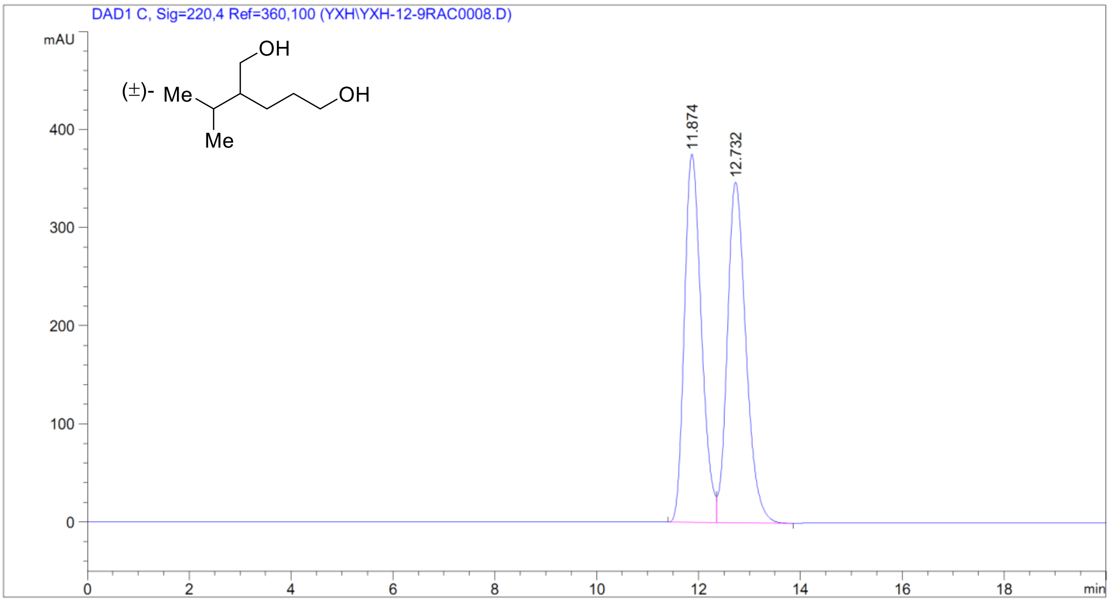

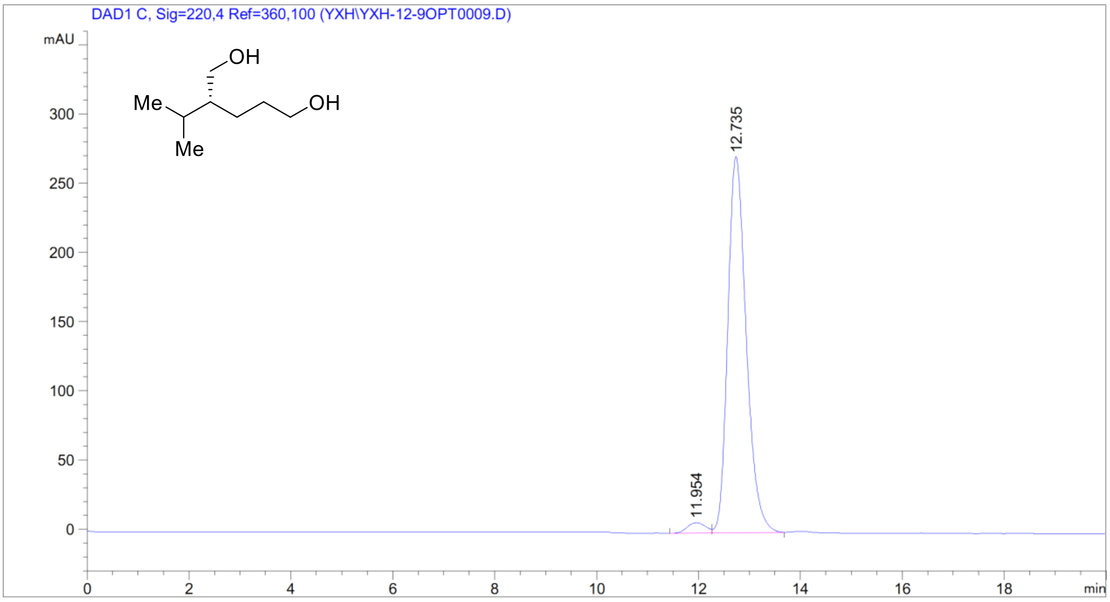

Peak #	RetTime [min]	Type	Width [min]	Area [mAU*s]	Height [mAU]	Area %
1	11.954	BV	0.4079	188.23749	7.37308	2.6820
2	12.735	VB	0.3873	6830.41455	271.68933	97.3180

(*S*)-2-(But-3-en-1-yl)pentane-1,5-diol (3p)

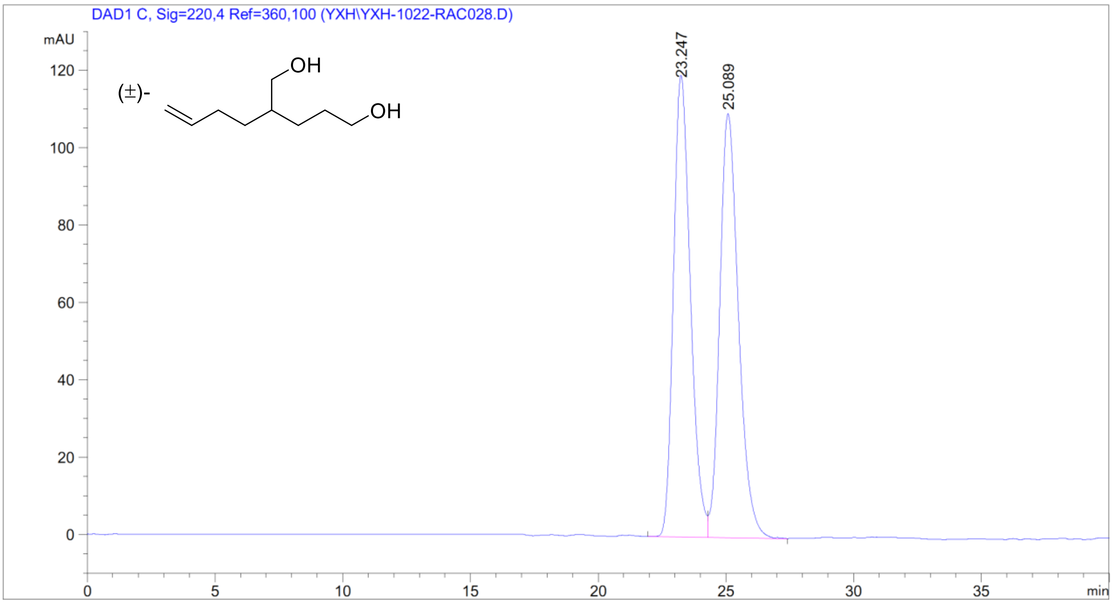

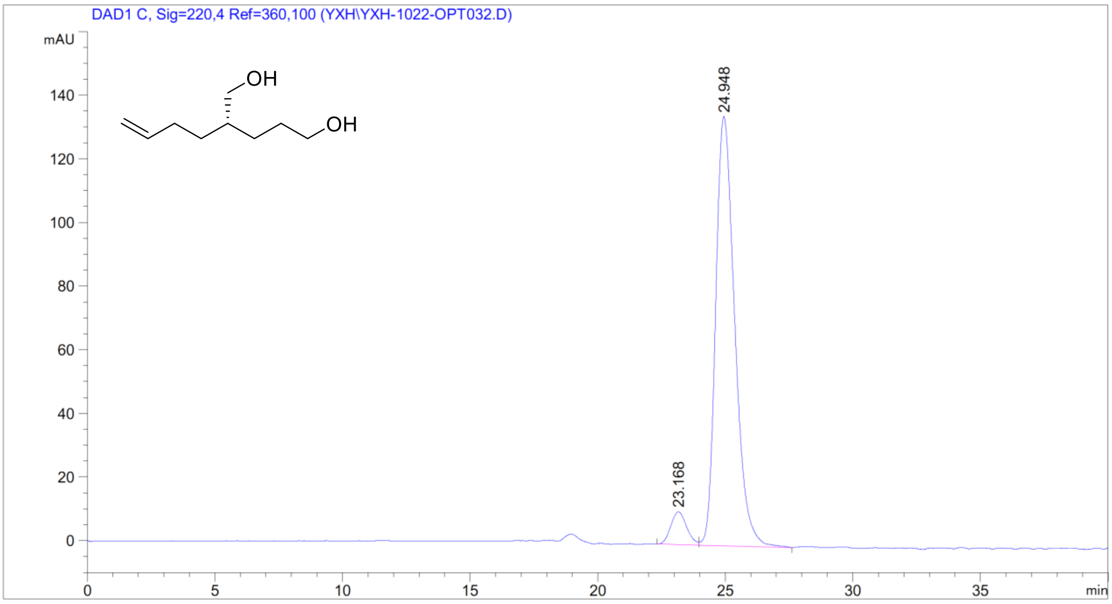

Peak #	RetTime [min]	Type	Width [min]	Area [mAU*s]	Height [mAU]	Area %
1	23.168	BV	0.6807	445.70706	10.30365	6.0002
2	24.948	VB	0.8042	6982.53613	134.82855	93.9998

(*R*)-2-Phenylbutane-1,4-diol (3q)

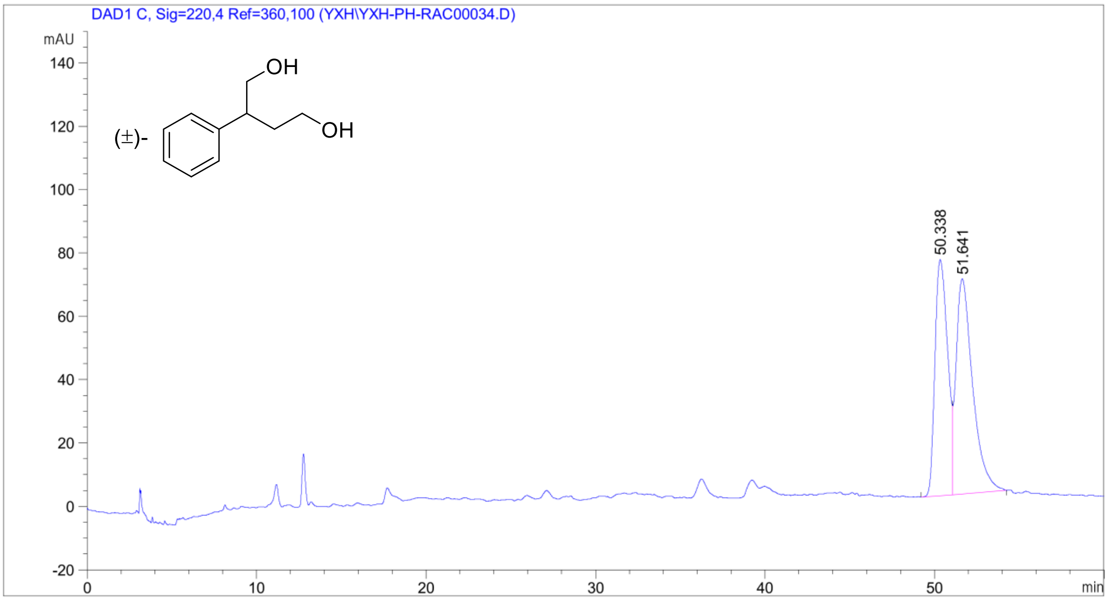

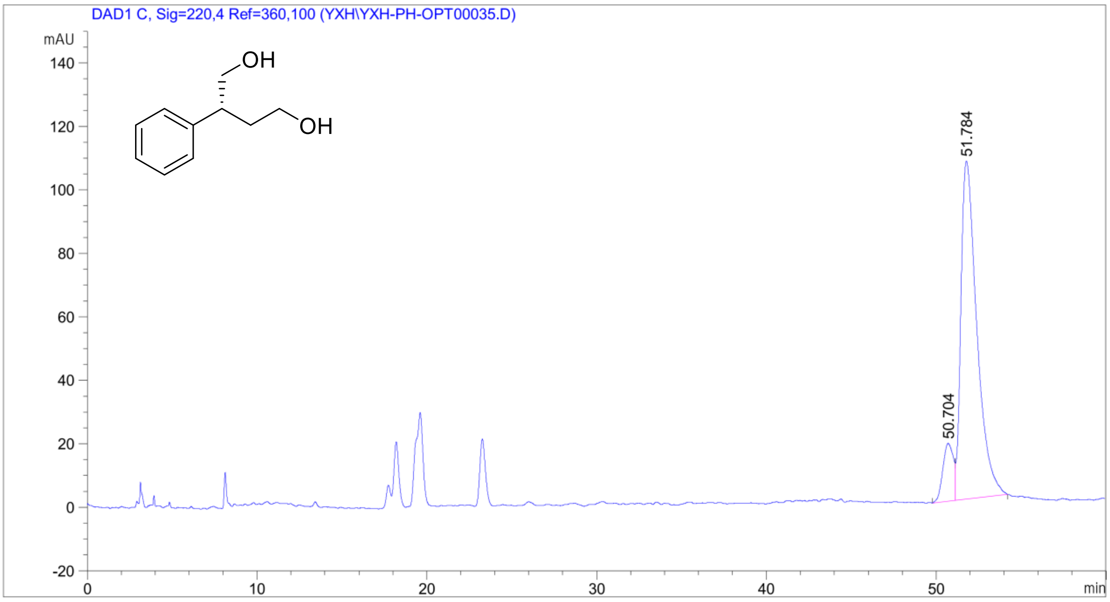

Peak #	RetTime [min]	Type	Width [min]	Area [mAU*s]	Height [mAU]	Area %
1	50.704	BV	0.6413	786.82959	18.23841	10.1960
2	51.784	VB	0.9732	6930.21094	106.49490	89.8040

(*S*)-2-Methylbutane-1,4-diol (3r)

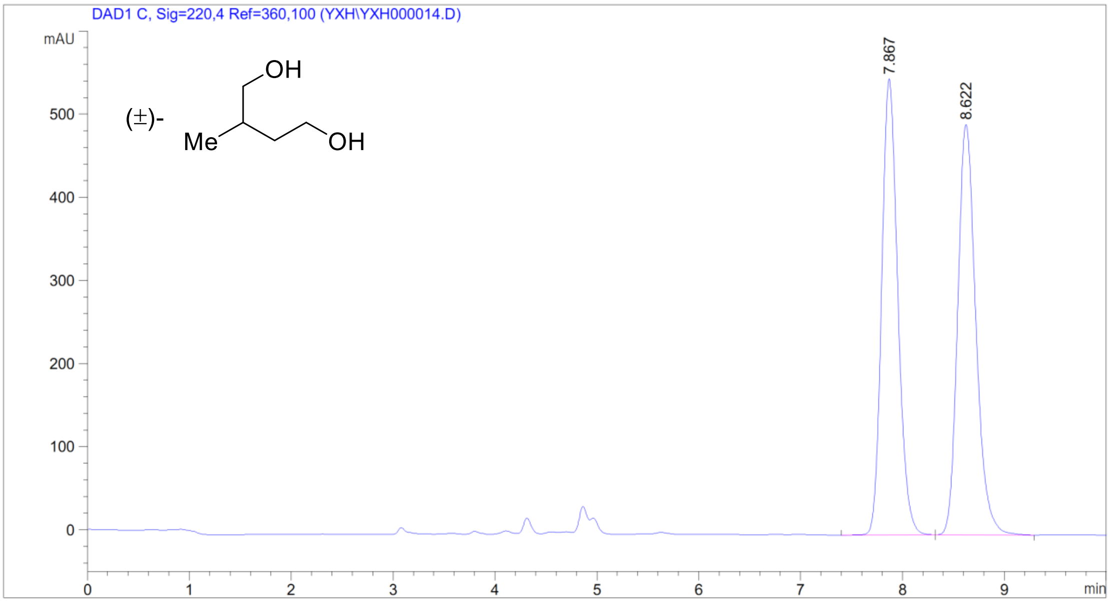

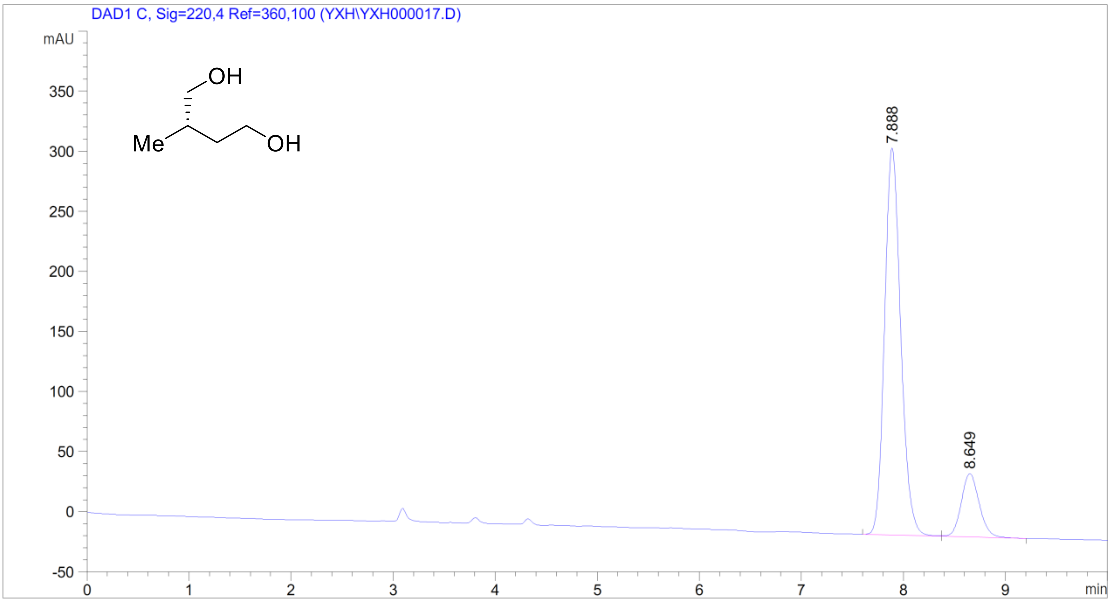

Peak #	RetTime [min]	Type	Width [min]	Area [mAU*s]	Height [mAU]	Area %
1	7.888	BV	0.1700	3533.73608	322.14230	84.5679
2	8.649	VB	0.1865	644.84155	52.79661	15.4321

The HPLC of asymmetric hydrogenation of hydroxyl ester 4 to (*R*)-2-pPhenylpentane-1,5-diol (3a)

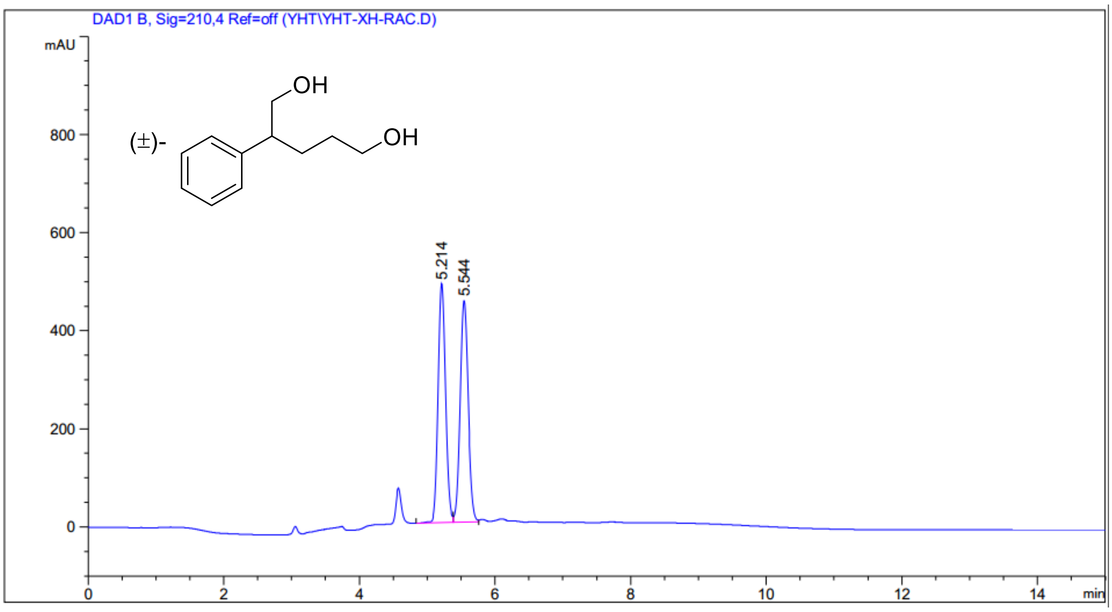

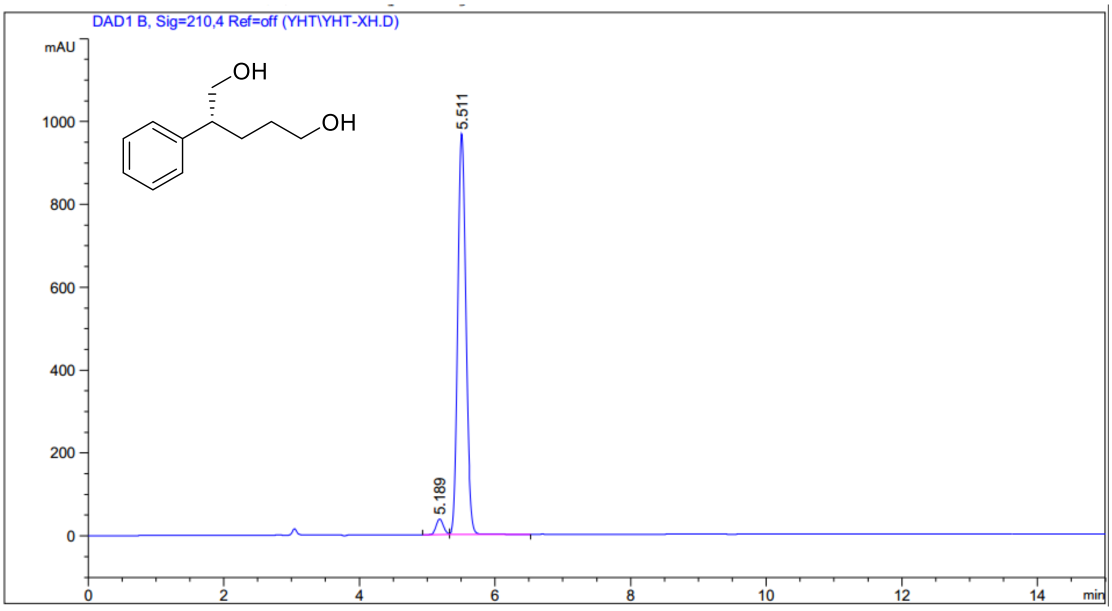

Peak #	RetTime [min]	Type	Width [min]	Area [mAU*s]	Height [mAU]	Area %
1	5.189	BV	0.1168	278.99084	37.49646	3.3630
2	5.511	VB	0.1287	8016.83057	967.50275	96.6370
